# Supplementary material for: The Theory of Planned Behaviour doesn’t reveal ’attitude-behaviour’ gap? Contrasting the effects of moral norms vs. idealism and relativism in predicting pro-environmental behaviours
Source: PLoS One. 2023 Nov 27;18(11):e0290818. doi: 10.1371/journal.pone.0290818 (PMC10681191; doi:10.1371/journal.pone.0290818)
Supplement: S1 Fig — (PDF) [file pone.0290818.s001.pdf]

Model fit:
χ2/df = 1.50
p = .008
CFI = .975
RMSEA = .053
SRMR = .0390
TLI = .966

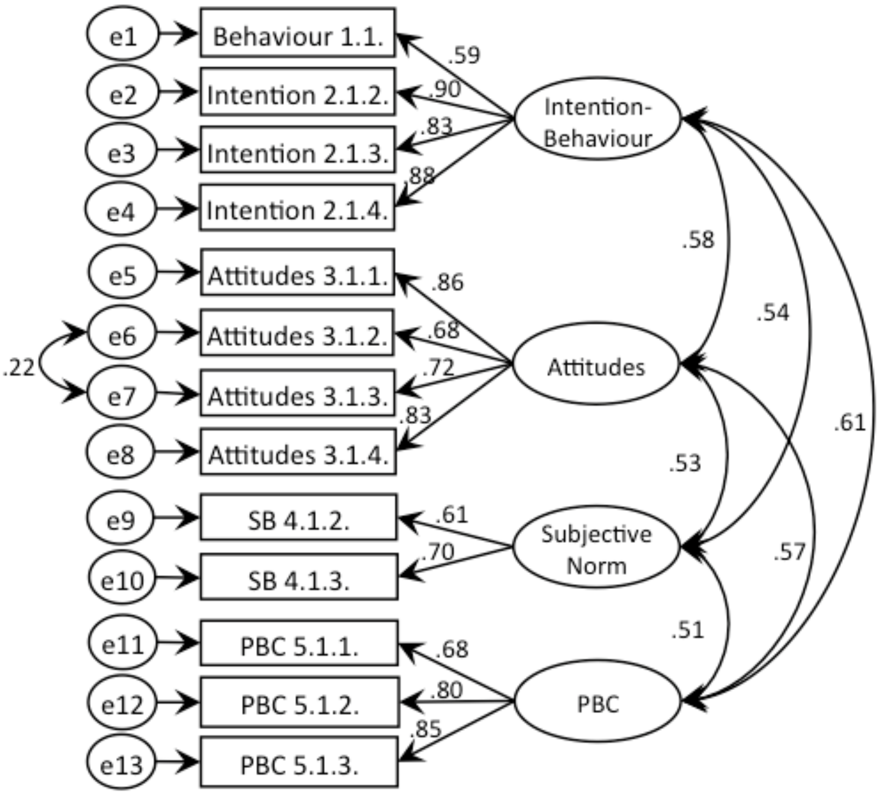

S1 Fig A. CFA, behaviour 1 (recycling): original TPB (Model 1).

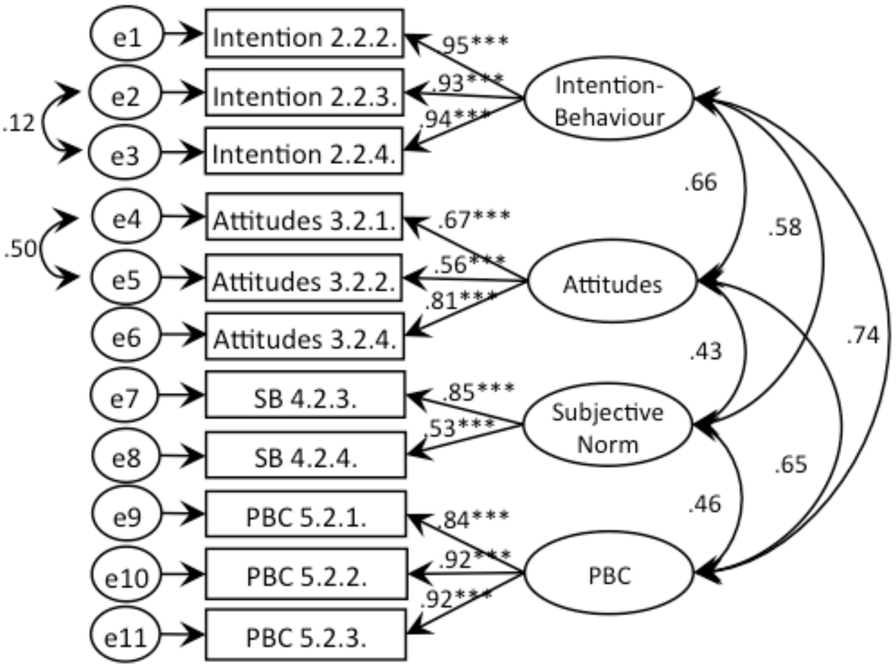

Model fit:
χ2/df = 1.37
p = .068
CFI = .991
RMSEA = .045
SRMR = .0326
TLI = .986

S1 Fig B. CFA, behaviour 2 (composting): original TPB (Model 1).

Model fit:
χ2/df = 1.34
p = .057
CFI = .986
RMSEA = .044
SRMR = .0485
TLI = .981

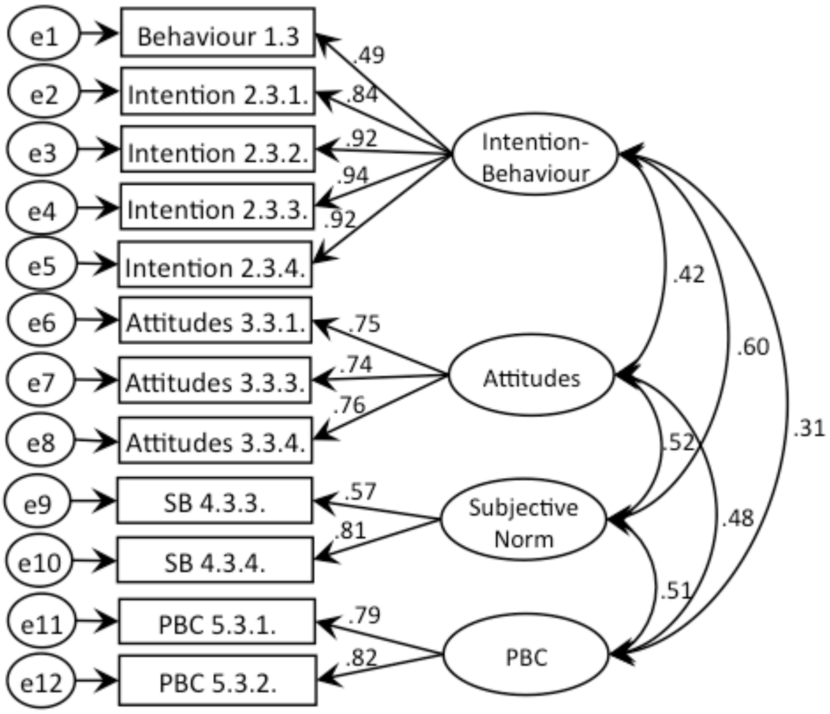

S1 Fig C. CFA, behaviour 3 (el. devices): original TPB (Model 1).

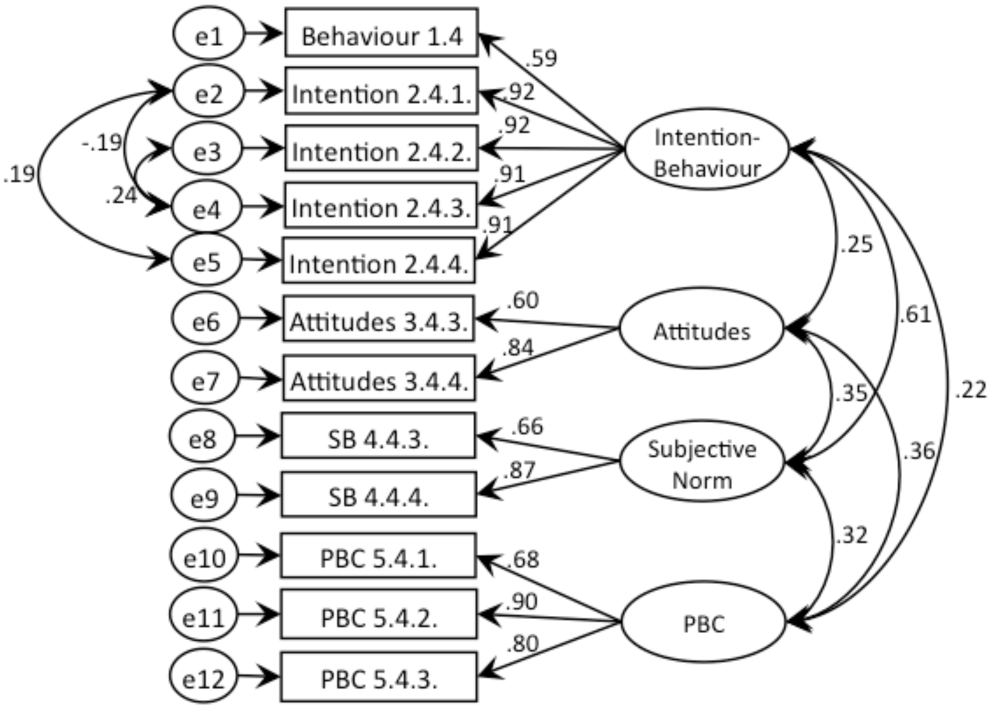

Model fit:
χ2/df =
p = .008
CFI = .980
RMSEA = .057
SRMR = .0638
TLI = .971

S1 Fig D. CFA, behaviour 4 (air cond.): original TPB (Model 1).

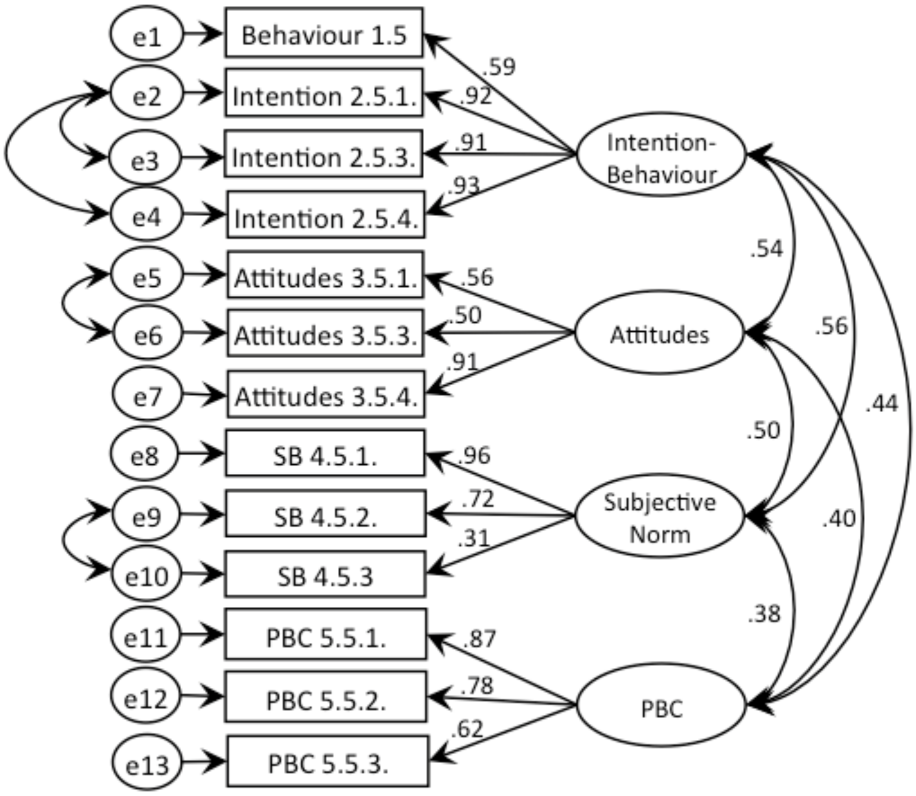

Model fit:
χ2/df = 1.66
p = .001
CFI = .971
RMSEA = .061
SRMR = .0493
TLI = .960

S1 Fig E. CFA, behaviour 5 (transport use): original TPB (Model 1).

Model fit:
χ2/df = 1.88
p = .009
CFI = .971
RMSEA = .070
SRMR = .0359
TLI = .950

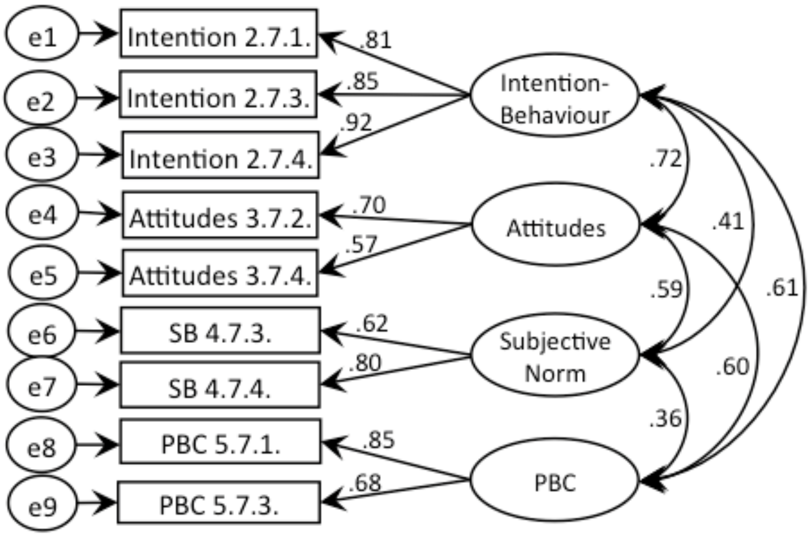

S1 Fig F. CFA, behaviour 7 (local products): original TPB (Model 1).

Model fit:
$\chi 2/df$ = 1.65
p = .007
CFI = .979
RMSEA = .060
SRMR = .0422
TLI = .970

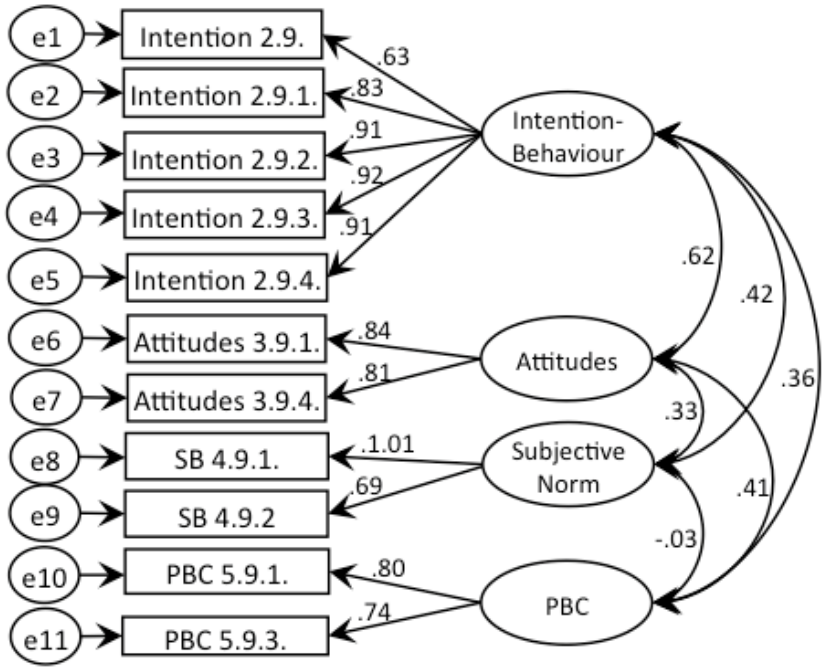

S1 Fig G. CFA, behaviour 9 (plastic bags): original TPB (Model 1).
