# Supplementary material for: The Theory of Planned Behaviour doesn’t reveal ’attitude-behaviour’ gap? Contrasting the effects of moral norms vs. idealism and relativism in predicting pro-environmental behaviours
Source: PLoS One. 2023 Nov 27;18(11):e0290818. doi: 10.1371/journal.pone.0290818 (PMC10681191; doi:10.1371/journal.pone.0290818)
Supplement: S2 Fig — (PDF) [file pone.0290818.s002.pdf]

Model fit:
$\chi 2/df$ = 1.83
p = .001
CFI = .969
RMSEA = .068 [.043, .092]
SRMR = .0376
TLI = .958

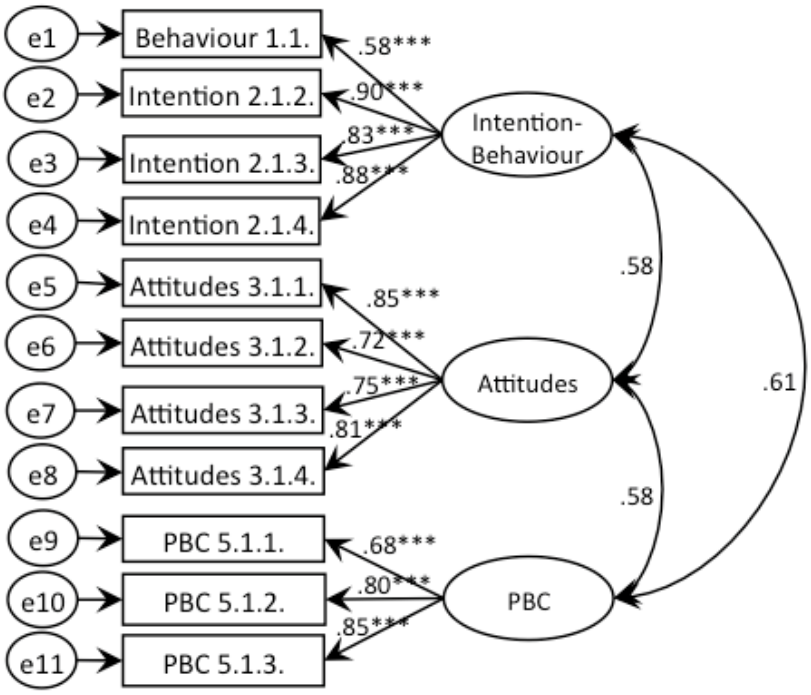

S2 Fig A. CFA, behaviour 1 (recycling): TPB without subjective norms (adjusted Model 1).

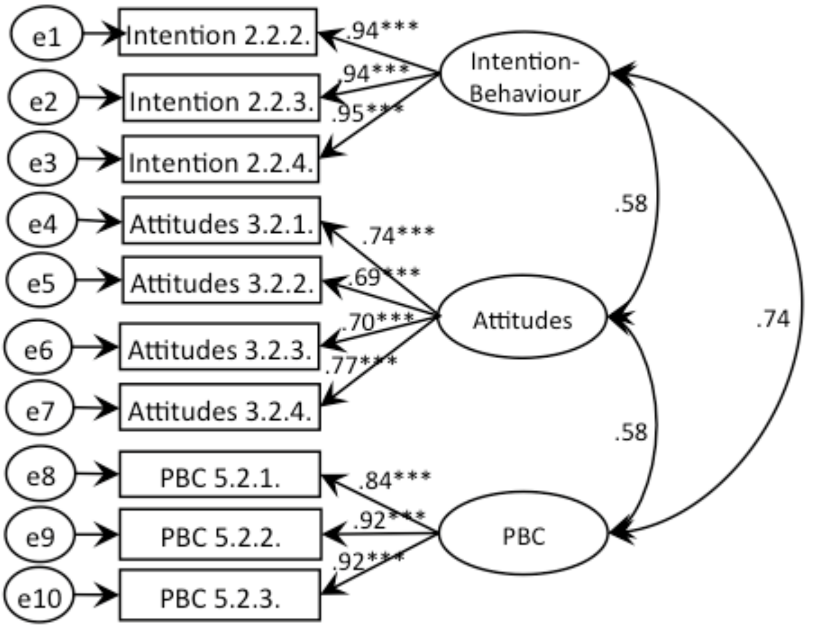

Model fit:
χ2/df = 1.94
p = .001
CFI = .981
RMSEA = .072 [.044, .099]
SRMR = .0529
TLI = .972

S2 Fig B. CFA, behaviour 2 (composting): TPB without subjective norms (adjusted Model 1).

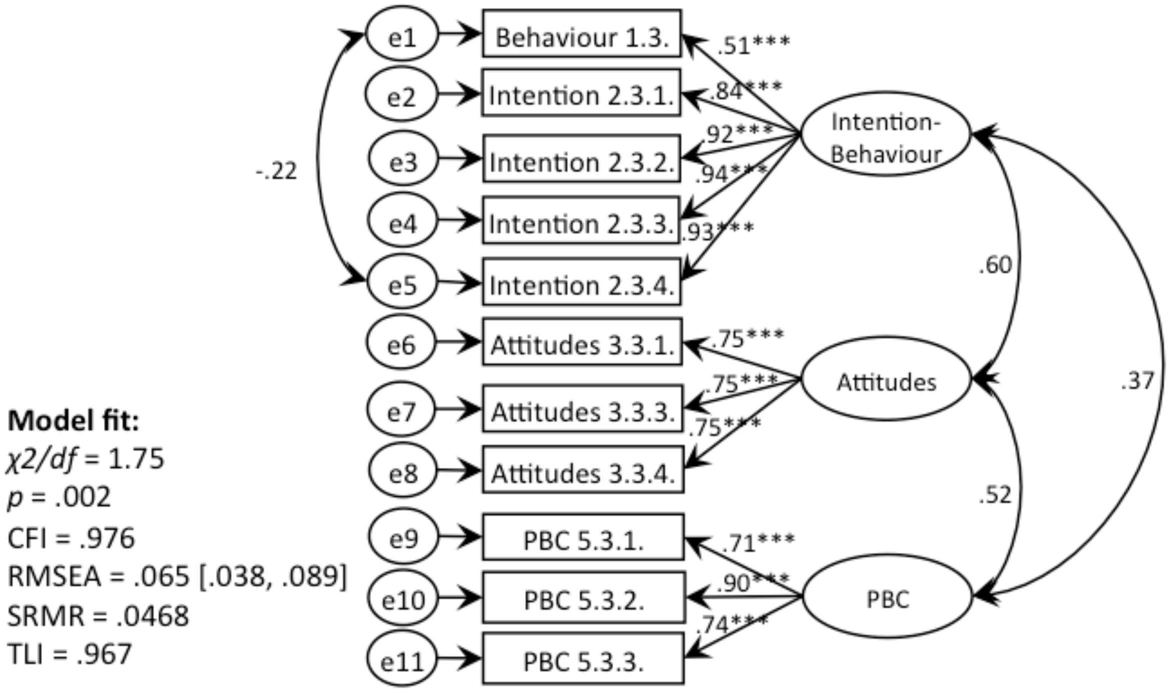

S2 Fig C. CFA, behaviour 3 (el. devices): TPB without subjective norms (adjusted Model 1).

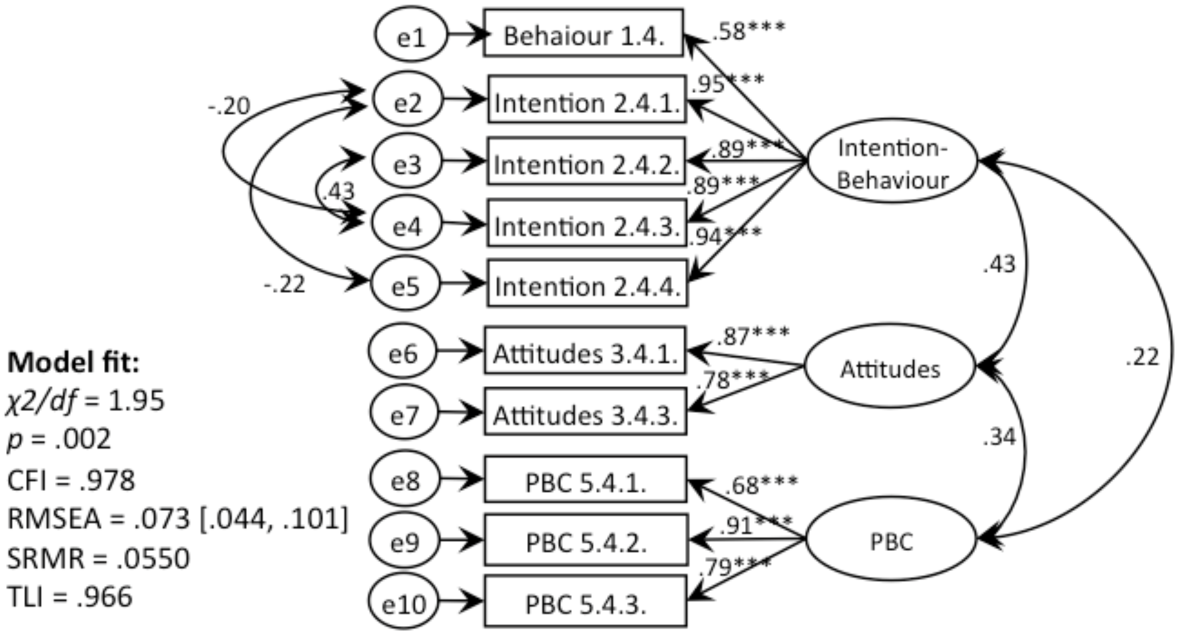

S2 Fig D. CFA, behaviour 4 (air cond.): TPB without subjective norms (adjusted Model 1).

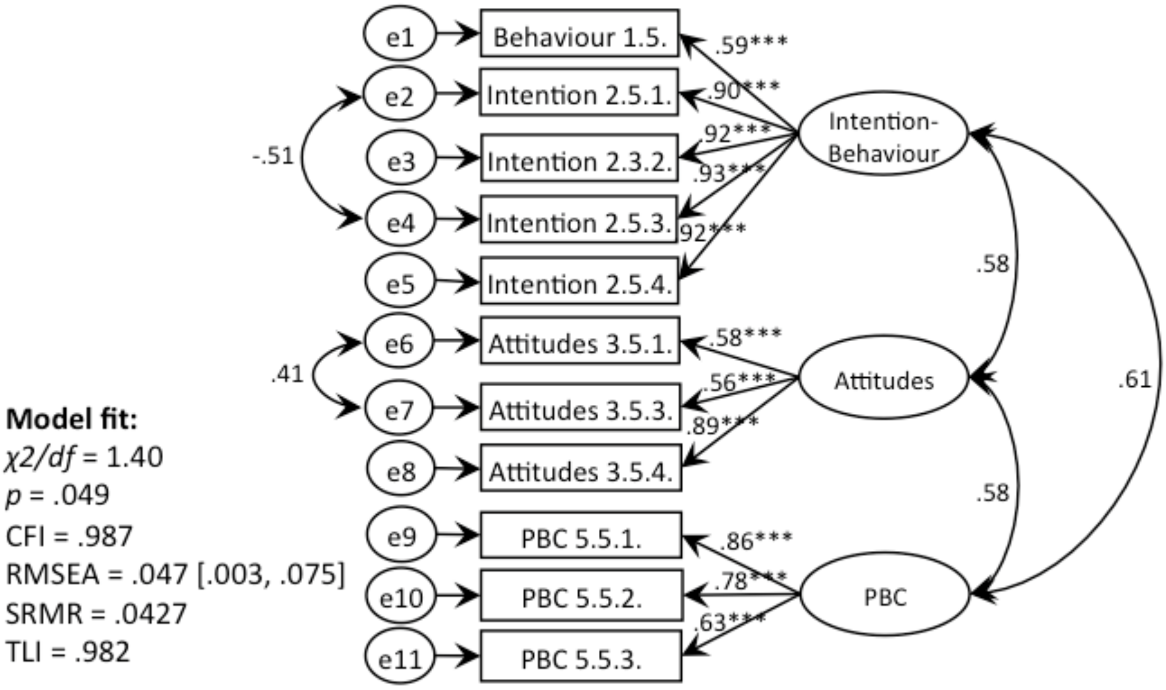

S2 Fig E. CFA, behaviour 5 (transport use): TPB without subjective norms (adjusted Model 1).

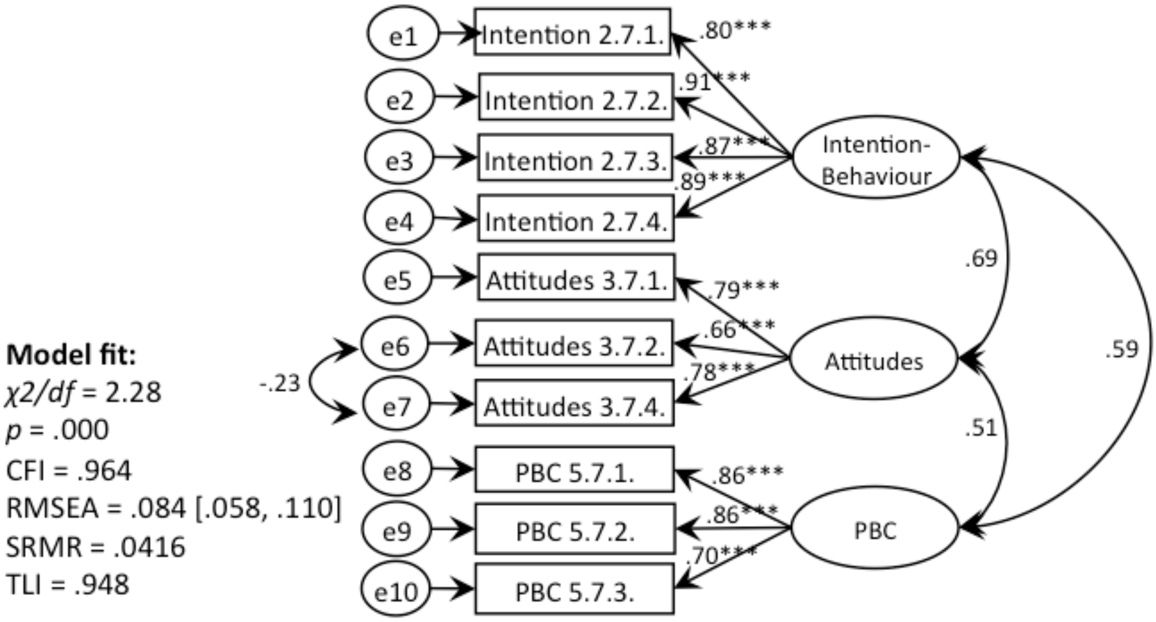

S2 Fig F. CFA, behaviour 7 (local products): TPB without subjective norms (adjusted Model 1).

Model fit:
$\chi2/df$ = 1.75
p = .002
CFI = .975
RMSEA = .065 [.039, .089]
SRMR = .0405
TLI = .967

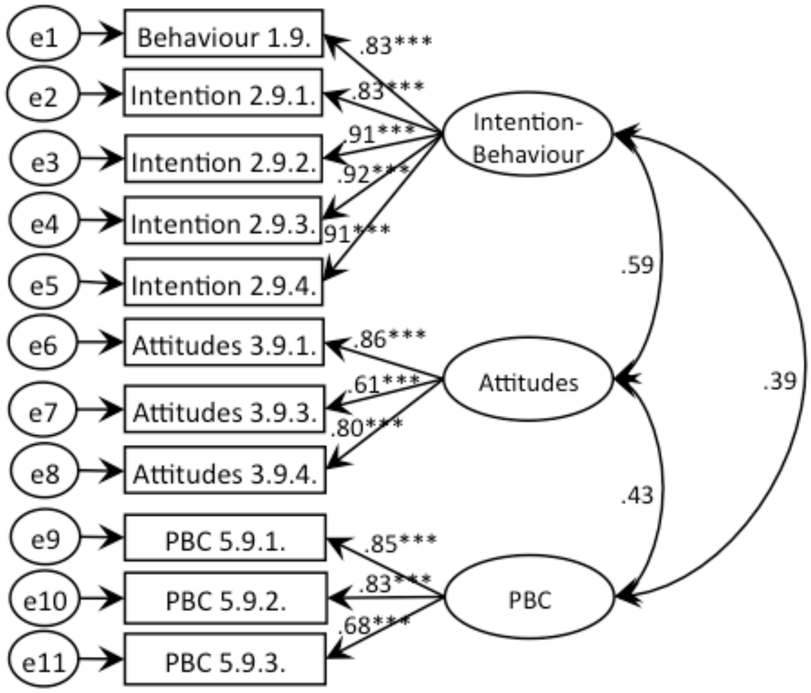

S2 Fig G. CFA, behaviour 9 (plastic bags): TPB without subjective norms (adjusted Model 1).
