# Supplementary material for: The Theory of Planned Behaviour doesn’t reveal ’attitude-behaviour’ gap? Contrasting the effects of moral norms vs. idealism and relativism in predicting pro-environmental behaviours
Source: PLoS One. 2023 Nov 27;18(11):e0290818. doi: 10.1371/journal.pone.0290818 (PMC10681191; doi:10.1371/journal.pone.0290818)
Supplement: S3 Fig — (PDF) [file pone.0290818.s003.pdf]

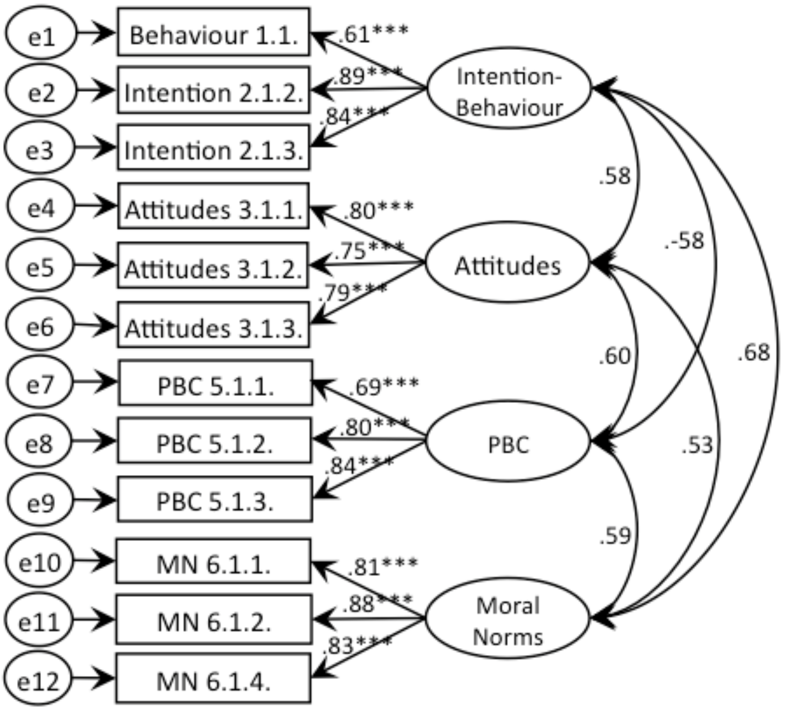

Model fit:
χ2/df = 1.51
p = .013
CFI = .978
RMSEA = .053 [.025, .077]
SRMR = .0389
TLI = .970

S3 Fig A. CFA, behaviour 1 (recycling): TPB with moral norms (adjusted Models 2, 4).

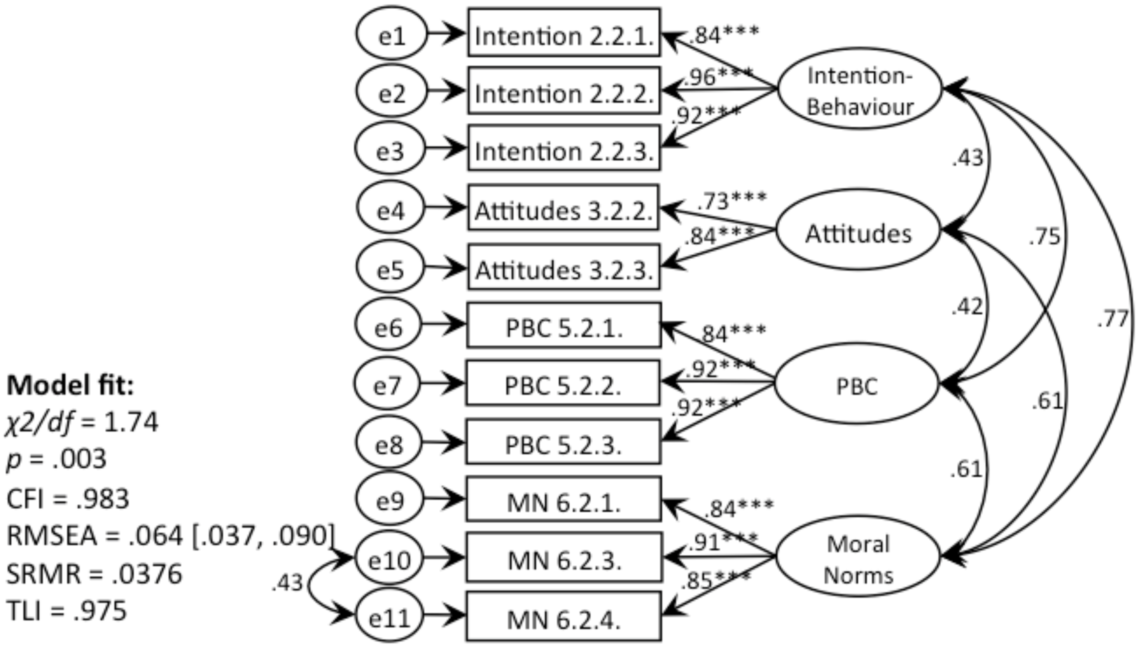

S3 Fig B. CFA, behaviour 2 (composting): TPB with moral norms (adjusted Models 2, 4).

Model fit:
$\chi 2/df$ = 1.56
p = .008
CFI = .982
RMSEA = .056 [.029, .079]
SRMR = .0342
TLI = .975

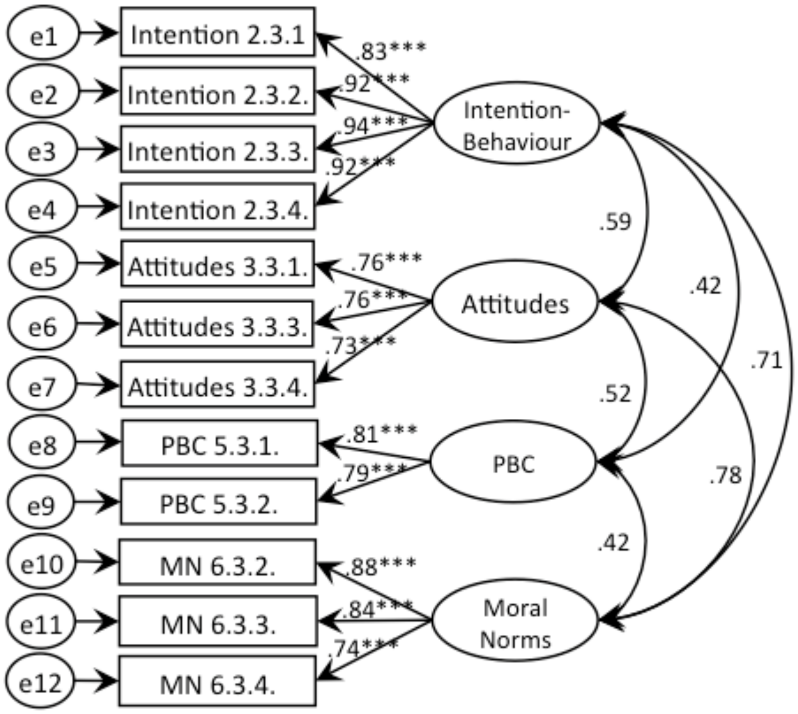

S3 Fig C. CFA, behaviour 3 (el. devices): TPB with moral norms (adjusted Models 2, 4).

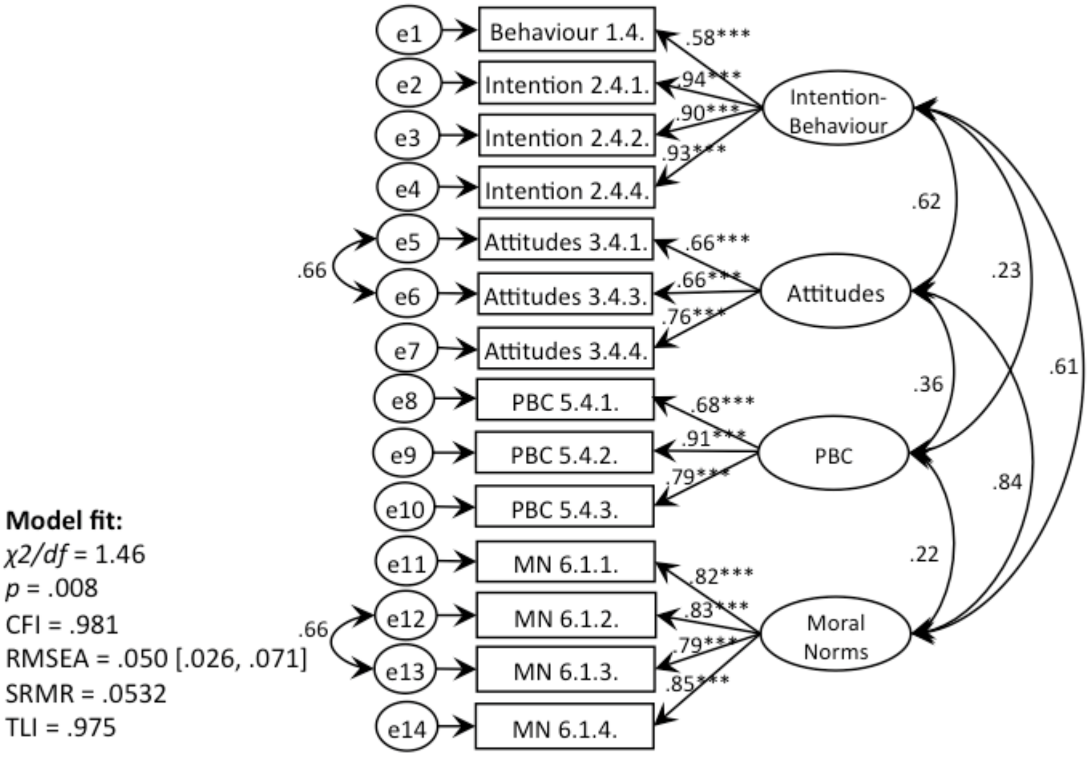

S3 Fig D. CFA, behaviour 4 (air cond.): TPB with moral norms (adjusted Models 2, 4).

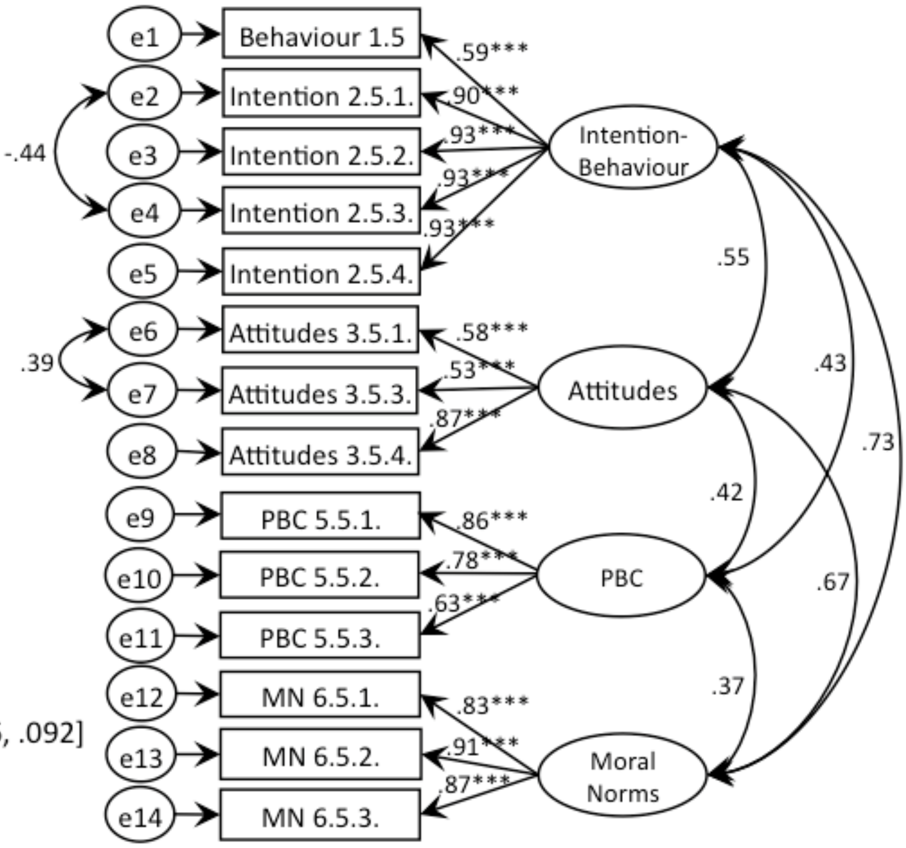

Model fit:
χ2/df = 1.99
p = .000
CFI = .960
RMSEA = .074 [.056, .092]
SRMR = .0578
TLI = .947

S3 Fig E. CFA, behaviour 5 (transport use): TPB with moral norms (adjusted Models 2, 4).

Model fit:
$\chi 2/df$ = 1.58
p = .012
CFI = .980
RMSEA = .057 [.027, .083]
SRMR = .0420
TLI = .971

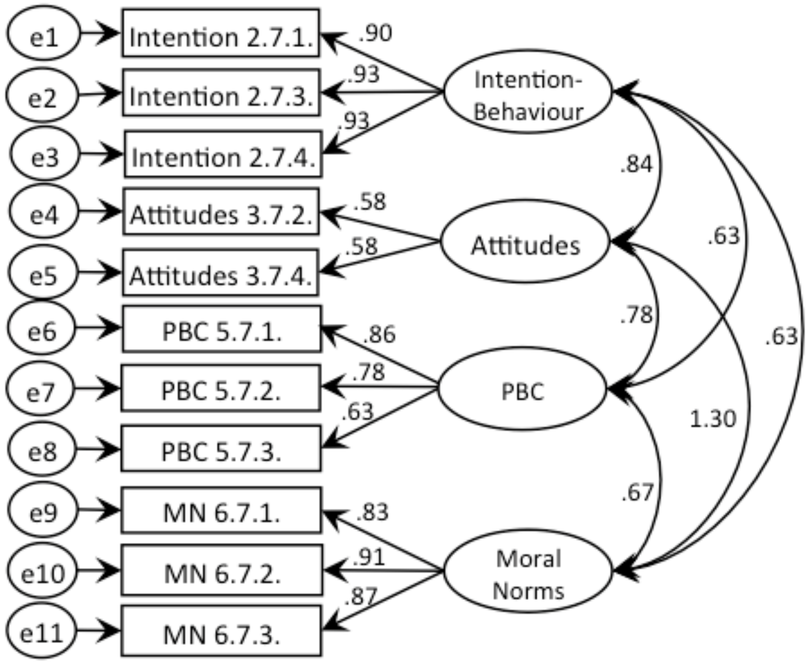

S3 Fig F. CFA, behaviour 7 (local products): TPB with moral norms (adjusted Models 2, 4).

Model fit:
$\chi 2/df$ = 1.42
p = .045
CFI = .985
RMSEA = .048 [.008, .076]
SRMR = .0352
TLI = .979

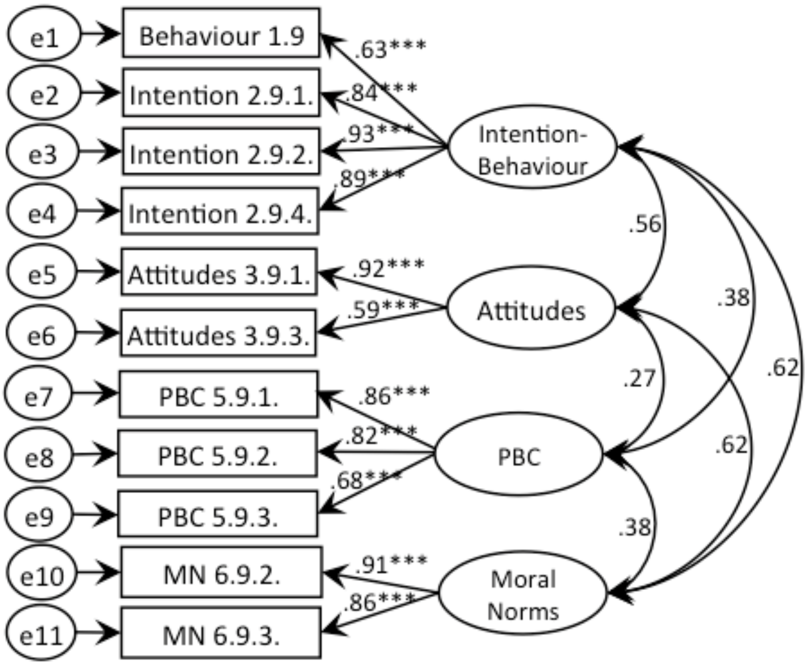

S3 Fig G. CFA, behaviour 9 (plastic bags): TPB with moral norms (adjusted Models 2, 4).
