# Supplementary material for: The Theory of Planned Behaviour doesn’t reveal ’attitude-behaviour’ gap? Contrasting the effects of moral norms vs. idealism and relativism in predicting pro-environmental behaviours
Source: PLoS One. 2023 Nov 27;18(11):e0290818. doi: 10.1371/journal.pone.0290818 (PMC10681191; doi:10.1371/journal.pone.0290818)
Supplement: S4 Fig — (PDF) [file pone.0290818.s004.pdf]

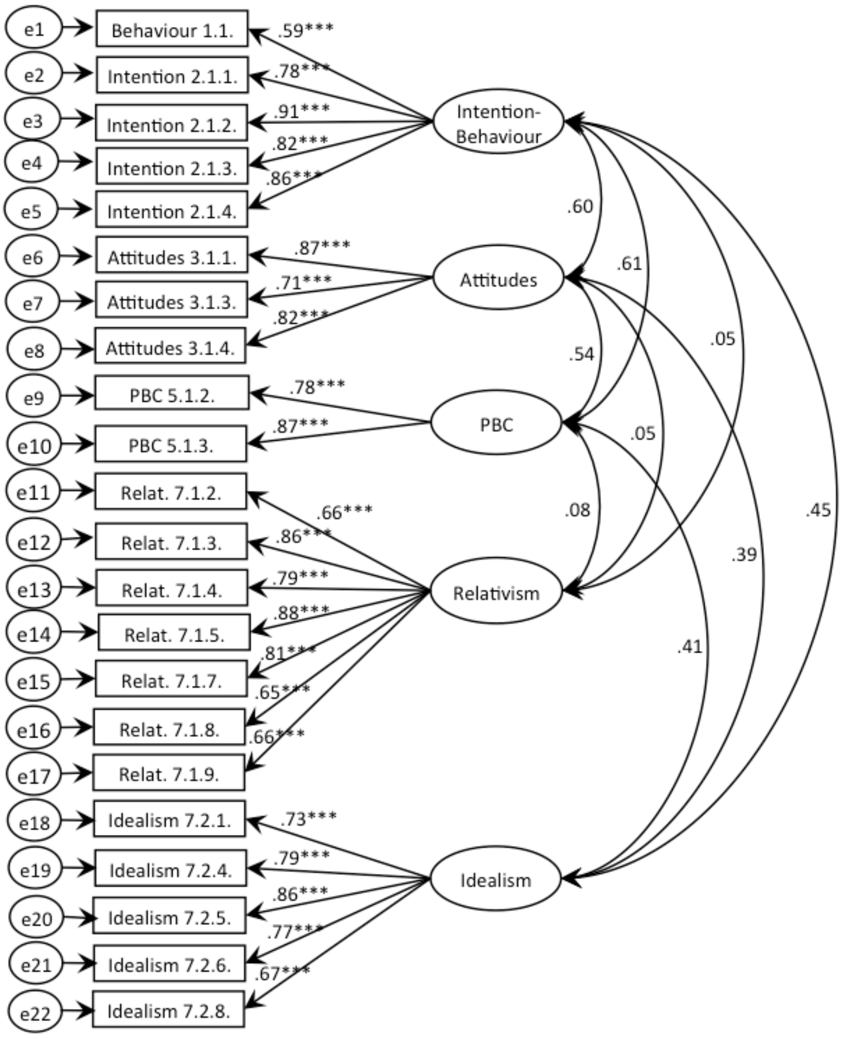

Model fit:
X2/df = 1.82
P = .000
CFI = .937
RMSEA = .064 [.052, .075]
SRMR = .0673
TLI= .928

S4 Fig A. CFA, behaviour 1 (recycling): TPB with EPQ (adjusted Models 3, 5).

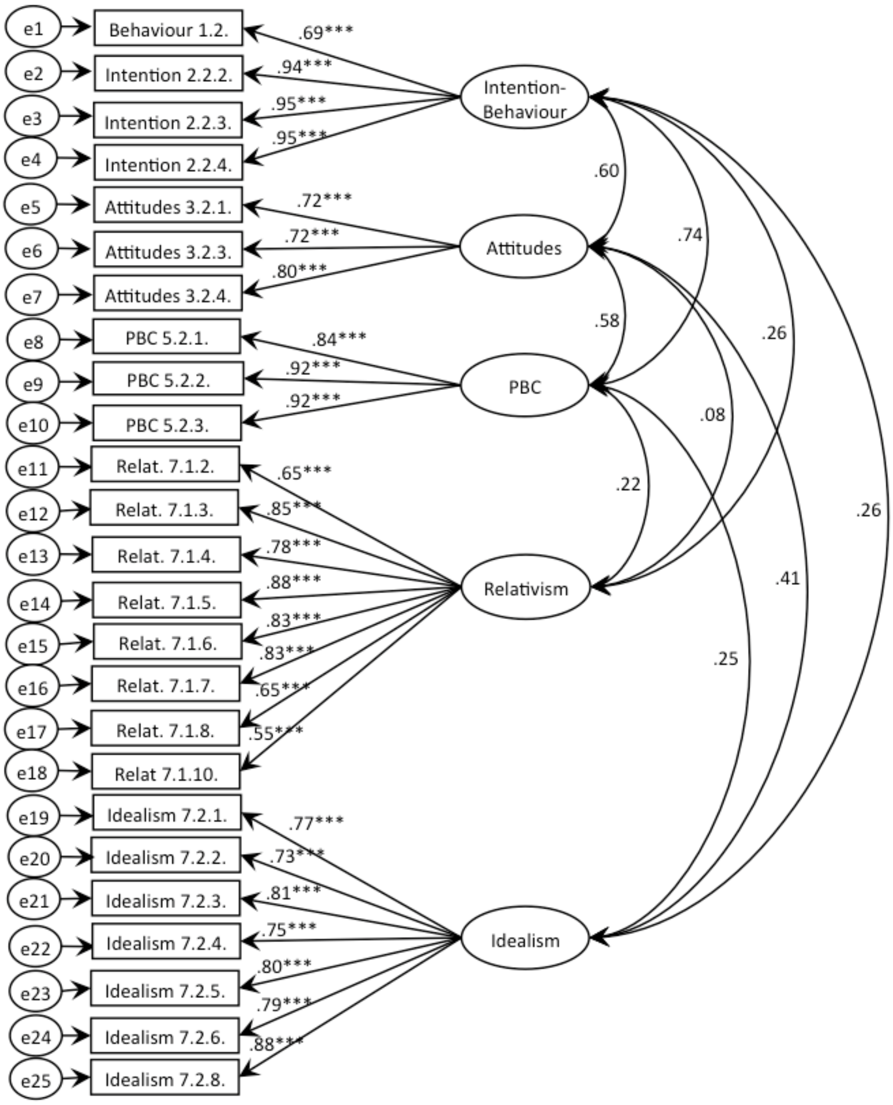

Model fit:
X2/df = 1.44
P = .000
CFI = .963
RMSEA = .050 [.030, .060]
SRMR = .0574
TLI= .958

S4 Fig B. CFA, behaviour 2 (composting): TPB with EPQ (adjusted Models 3, 5).

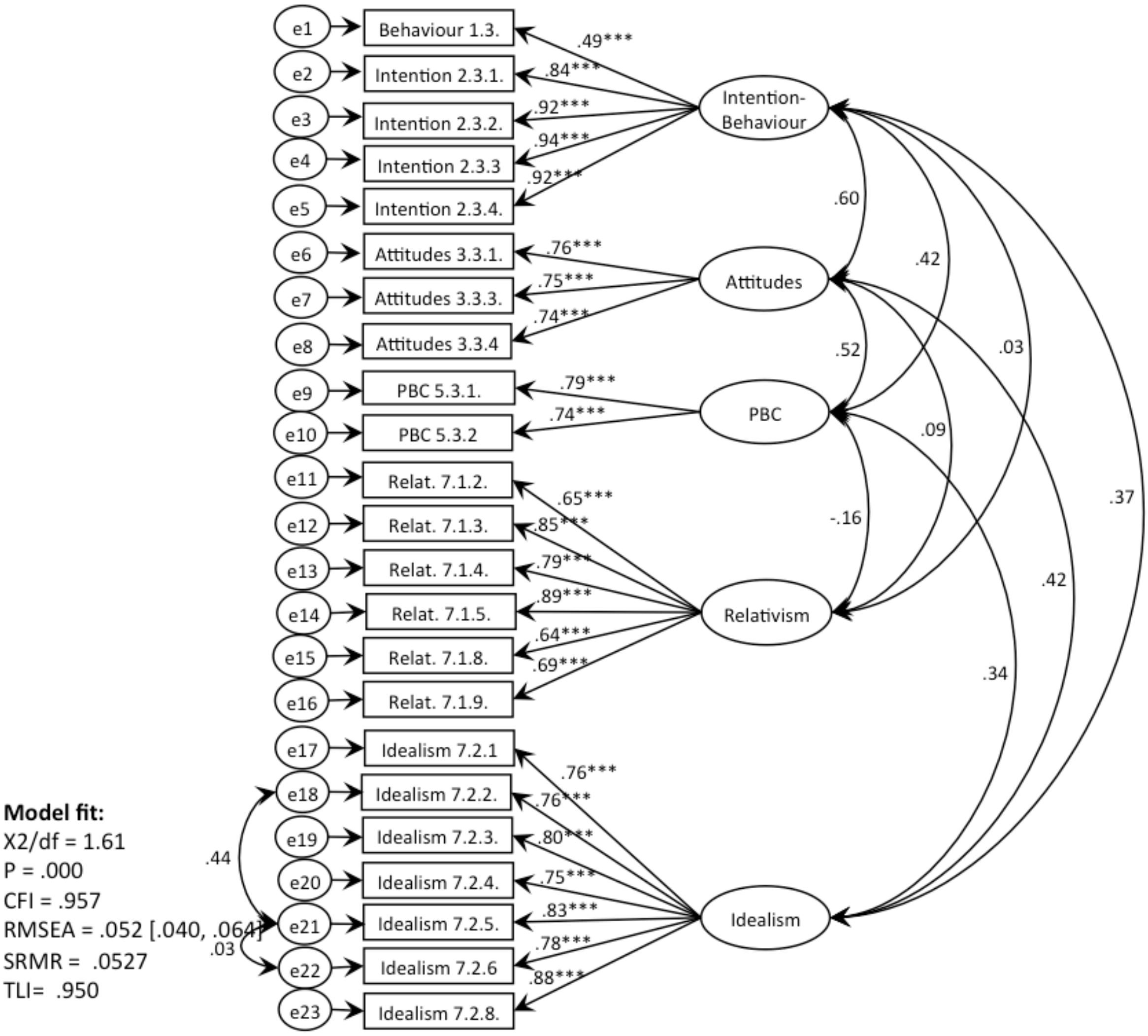

S4 Fig C. CFA, behaviour 3 (e. devices): TPB with EPQ (adjusted Models 3, 5).

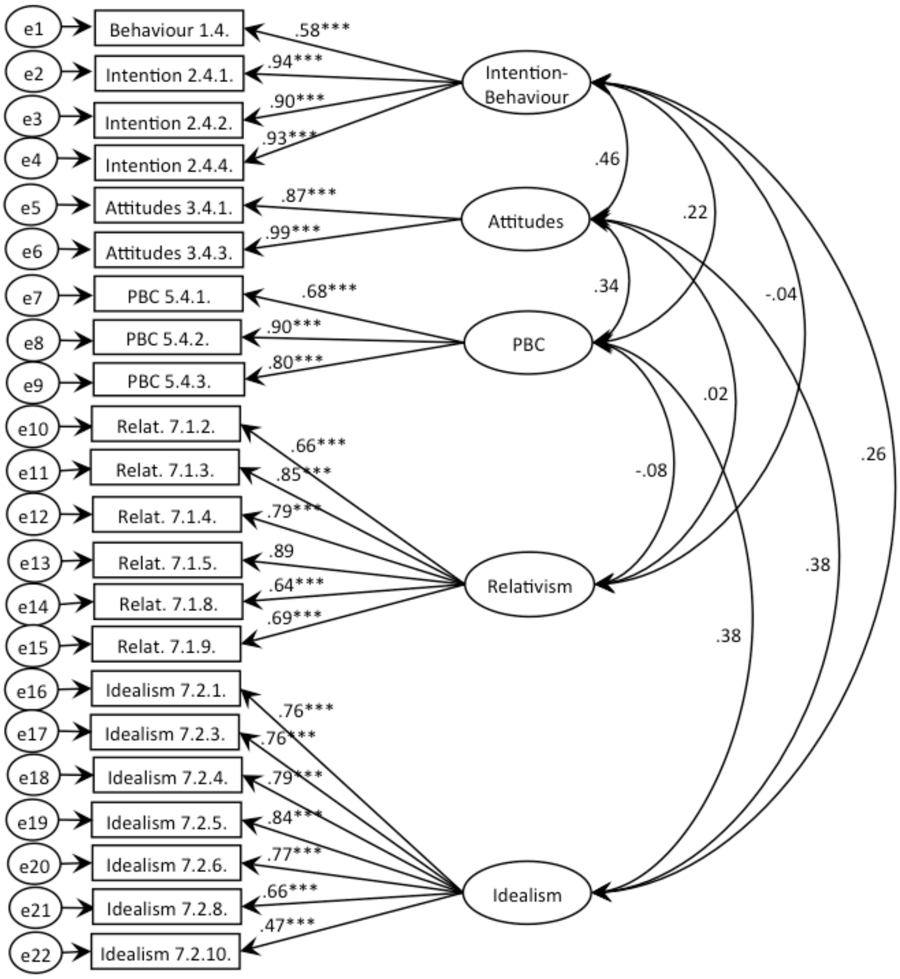

Model fit:
X2/df = 1.50
P = .000
CFI = .955
RMSEA = .53 [.40, .65]
SRMR = .0544
TLI= .948

S4 Fig D. CFA, behaviour 4 (air cond.): TPB with EPQ (adjusted Models 3, 5).

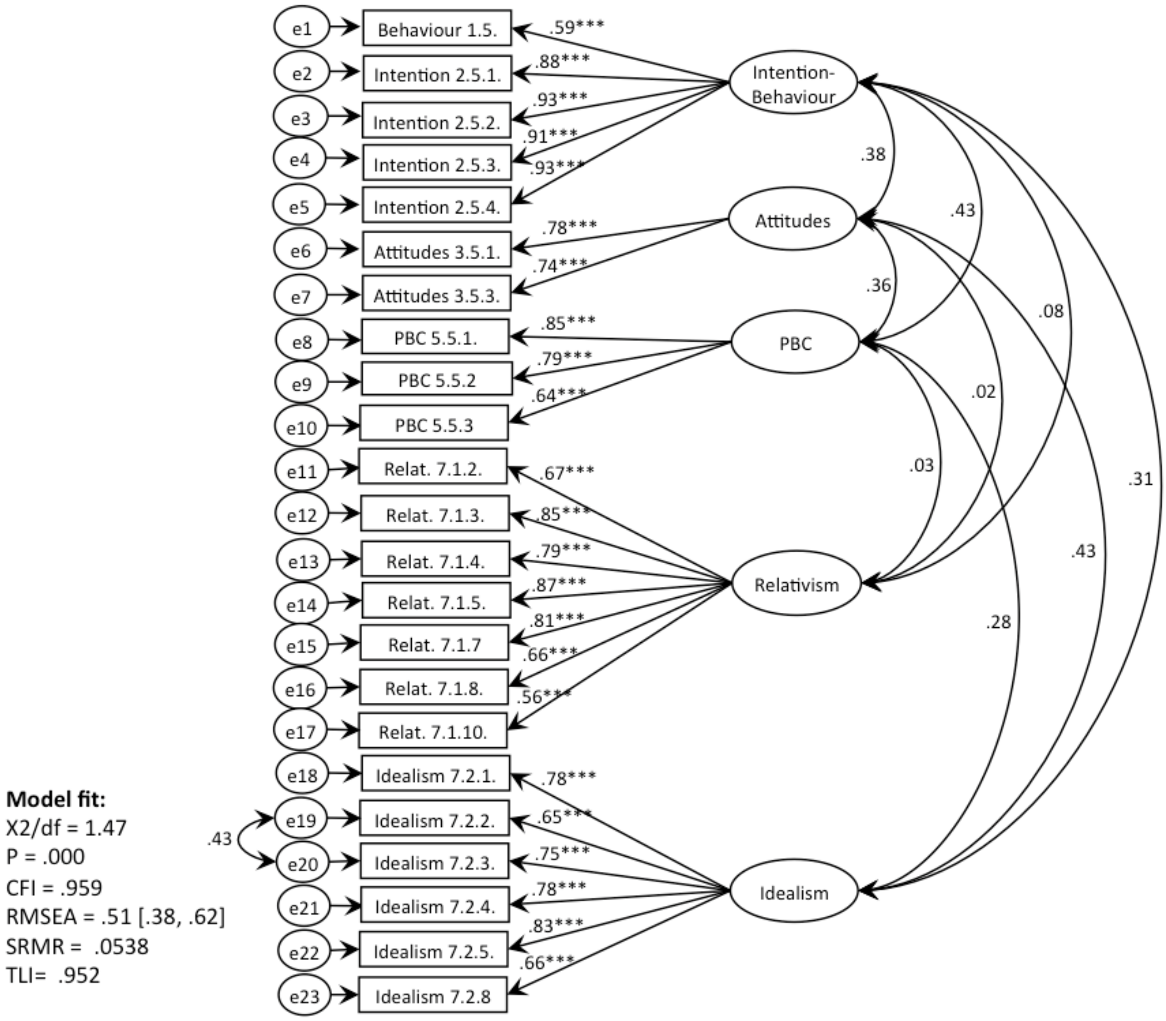

S4 Fig E. CFA, behaviour 5 (transport use): TPB with EPQ (adjusted Models 3, 5).

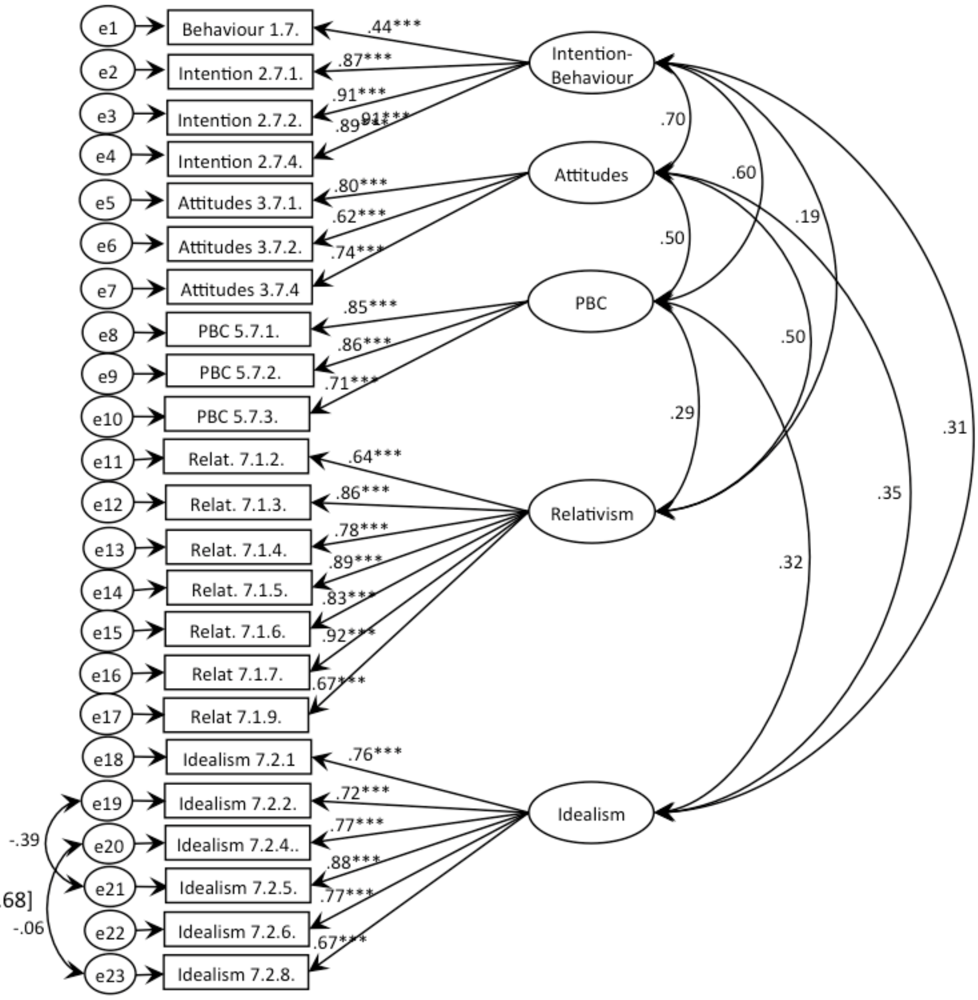

Model fit:
X2/df = 1.47
P = .000
CFI = .946
RMSEA = .57 [.46, .68]
SRMR = .0621
TLI= .937

S4 Fig F. CFA, behaviour 7 (local products): TPB with EPQ (adjusted Models 3, 5).

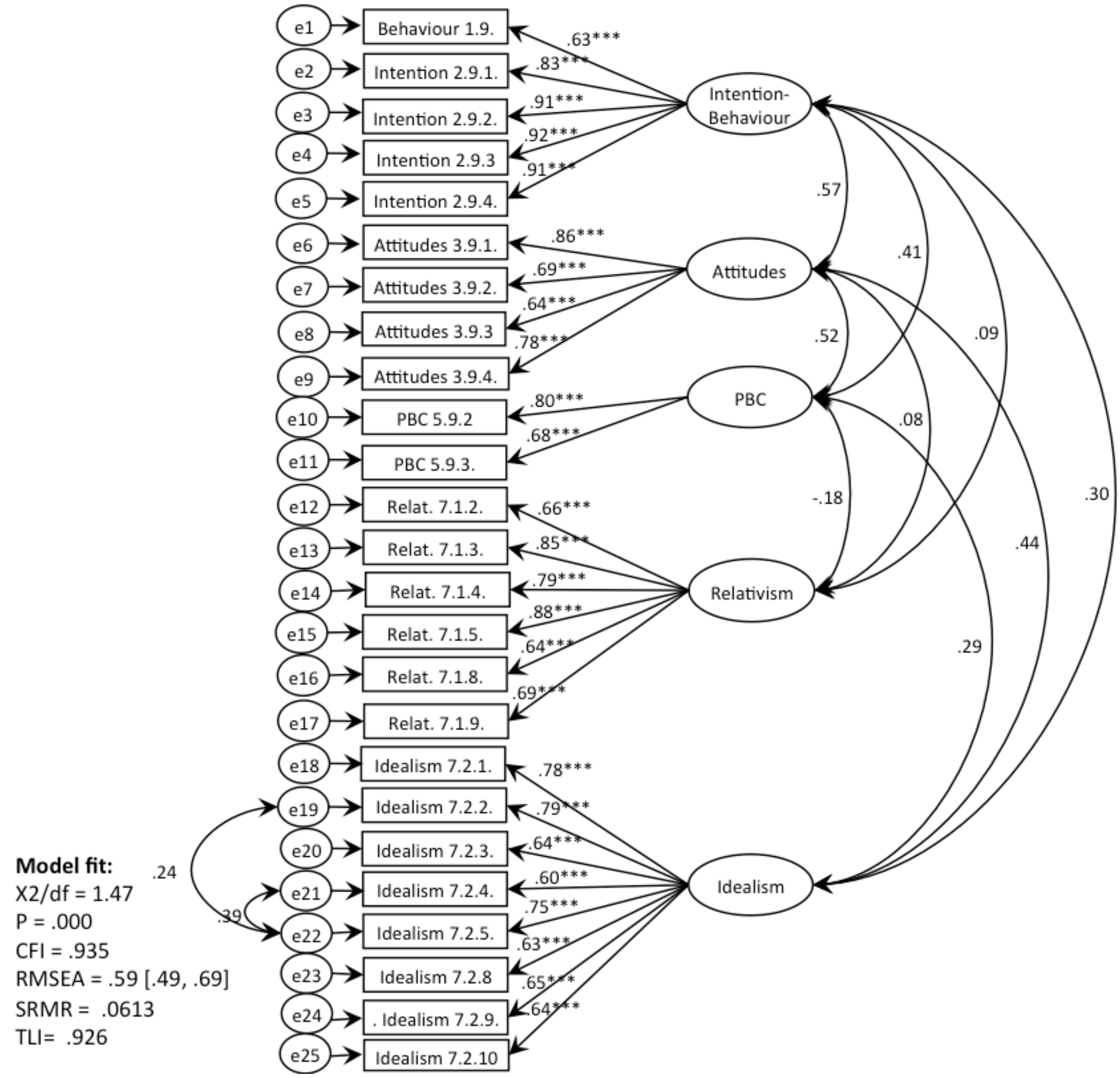

S4 Fig G. CFA, behaviour 9 (plastic bags): TPB with EPQ (adjusted Models 3, 5).
