# Supplementary material for: The Theory of Planned Behaviour doesn’t reveal ’attitude-behaviour’ gap? Contrasting the effects of moral norms vs. idealism and relativism in predicting pro-environmental behaviours
Source: PLoS One. 2023 Nov 27;18(11):e0290818. doi: 10.1371/journal.pone.0290818 (PMC10681191; doi:10.1371/journal.pone.0290818)
Supplement: S5 Fig — (PDF) [file pone.0290818.s005.pdf]

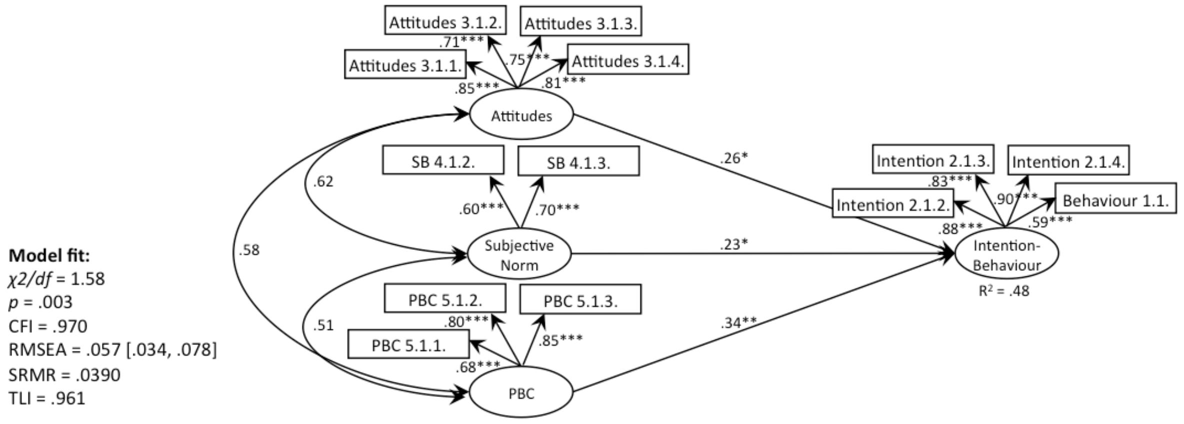

S5 Fig A. SEM, behaviour 1 (recycling): original TPB (Model 1).

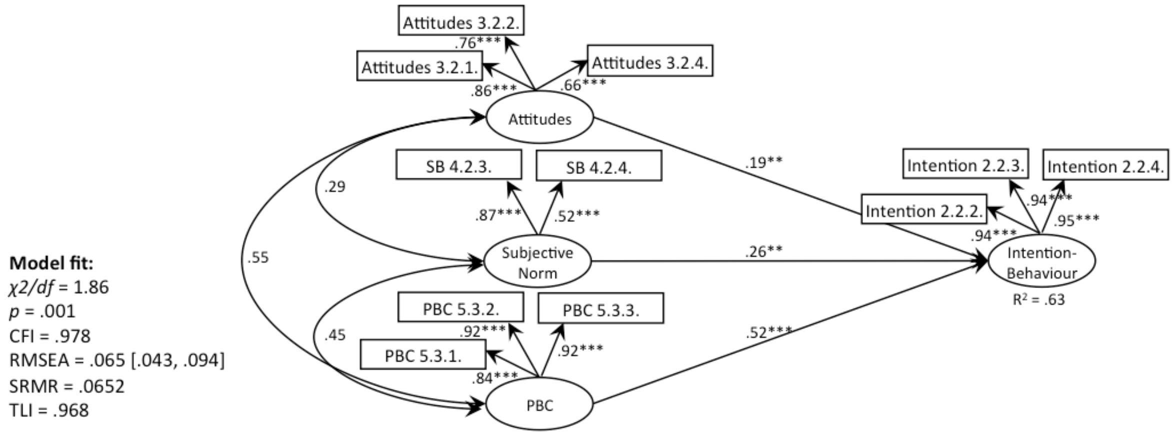

S5 Fig B. SEM, behaviour 2 (composting): original TPB (Model 1).

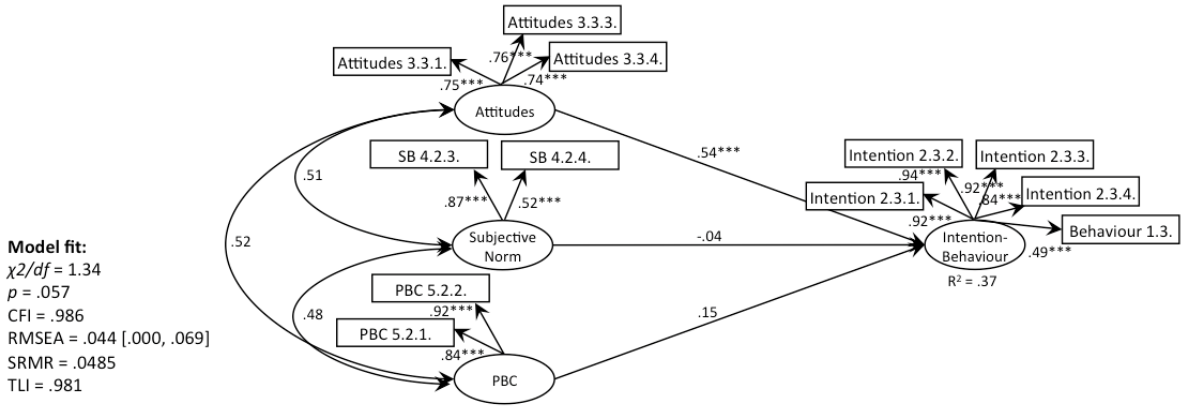

S5 Fig C. SEM, behaviour 3 (el. devices): original TPB (Model 1).

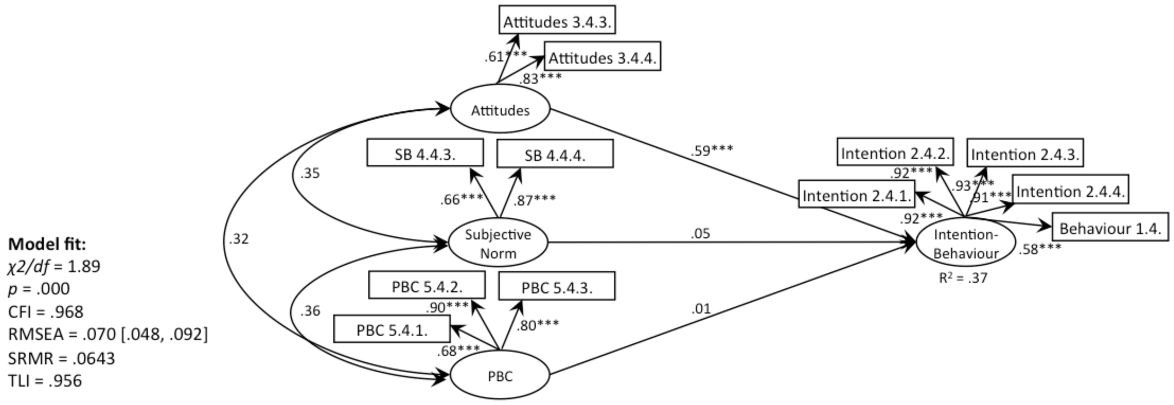

S5 Fig D. SEM, behaviour 4 (air cond.): original TPB (Model 1).

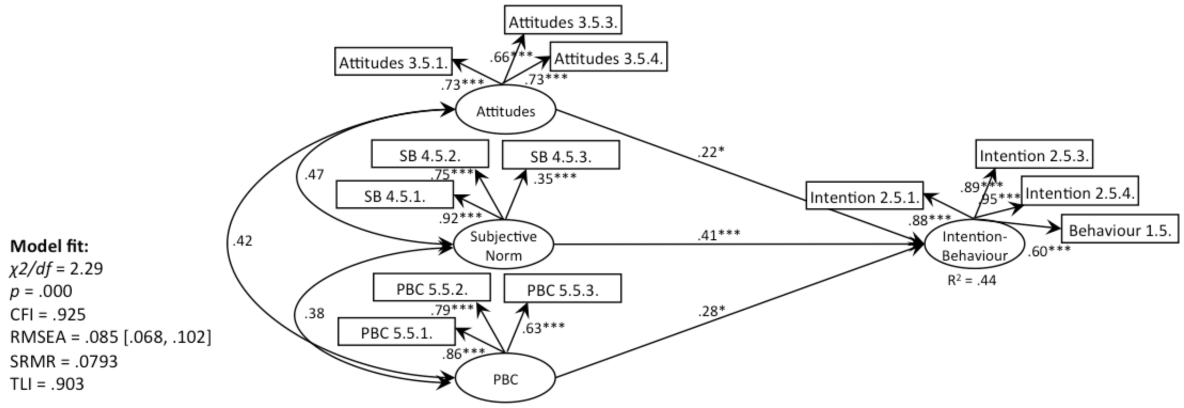

S5 Fig E. SEM, behaviour 5 (transport use): original TPB (Model 1).

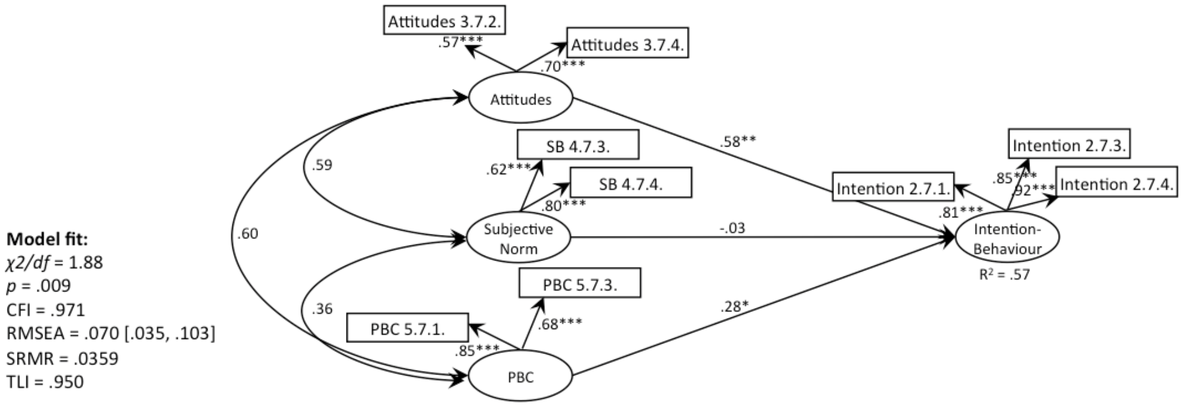

S5 Fig F. SEM, behaviour 7 (local products): original TPB (Model 1).

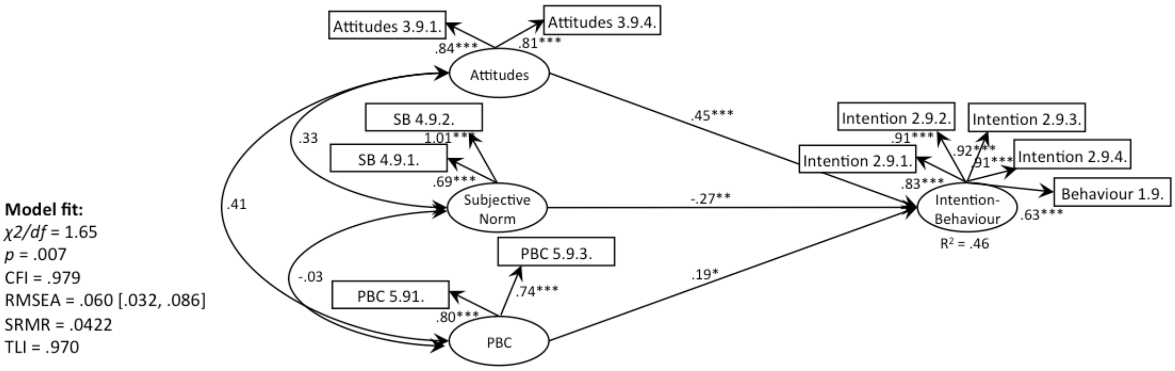

S5 Fig G. SEM, behaviour 7 (plastic bags): original TPB (Model 1).
