# Supplementary material for: The Theory of Planned Behaviour doesn’t reveal ’attitude-behaviour’ gap? Contrasting the effects of moral norms vs. idealism and relativism in predicting pro-environmental behaviours
Source: PLoS One. 2023 Nov 27;18(11):e0290818. doi: 10.1371/journal.pone.0290818 (PMC10681191; doi:10.1371/journal.pone.0290818)
Supplement: S7 Fig — (PDF) [file pone.0290818.s007.pdf]

Model fit:
χ2/df = 1.50
p = .013
CFI = .978
RMSEA = .053 [.025, .077]
SRMR = .0389
TLI = .970

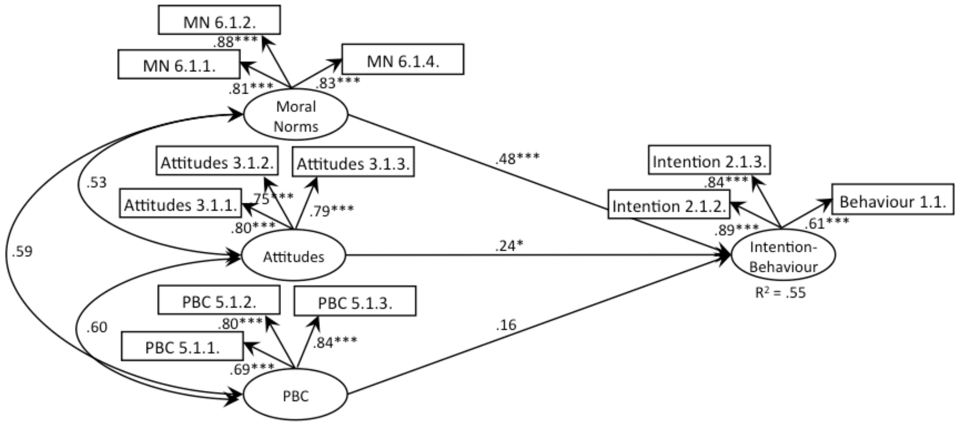

S7 Fig A. SEM, behaviour 1 (recycling): TPB with moral norms as additional predictor (adjusted Model 2).

Model fit:
χ2/df = 1.96
p = .000
CFI = .977
RMSEA = .073 [.048, .097]
SRMR = .0397
TLI = .967

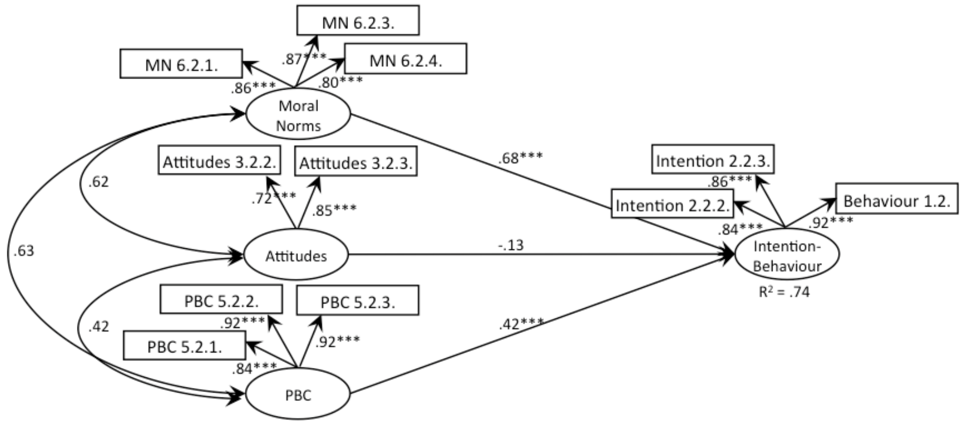

S7 Fig B. SEM, behaviour 2 (composting): TPB with moral norms as additional predictor (adjusted Model 2).

Model fit:
χ2/df = 1.56
p = .008
CFI = .982
RMSEA = .056 [.029, .079]
SRMR = .0342
TLI = .975

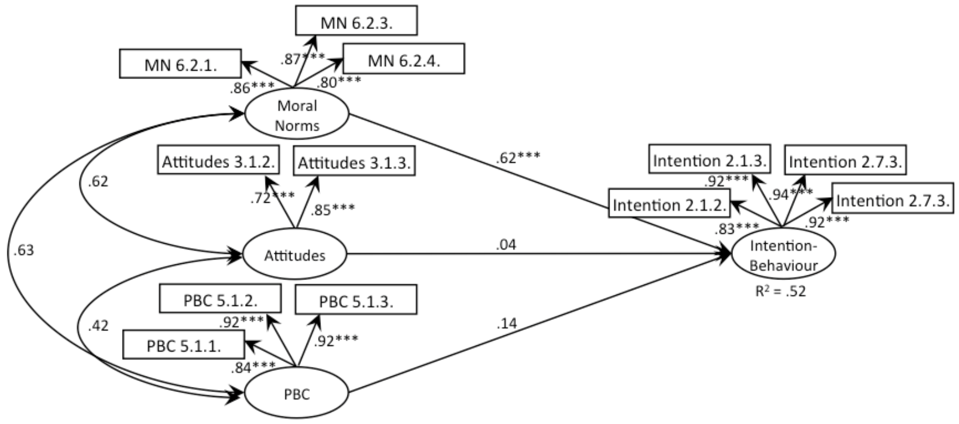

S7 Fig C. SEM, behaviour 3 (el. devices): TPB with moral norms as additional predictor (adjusted Model 2).

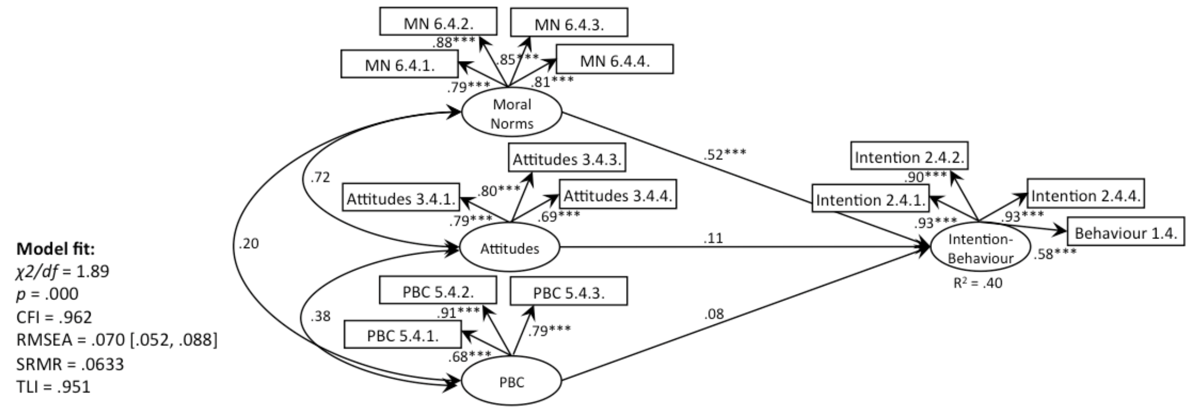

S7 Fig D. SEM, behaviour 4 (air cond.): TPB with moral norms as additional predictor (adjusted Model 2).

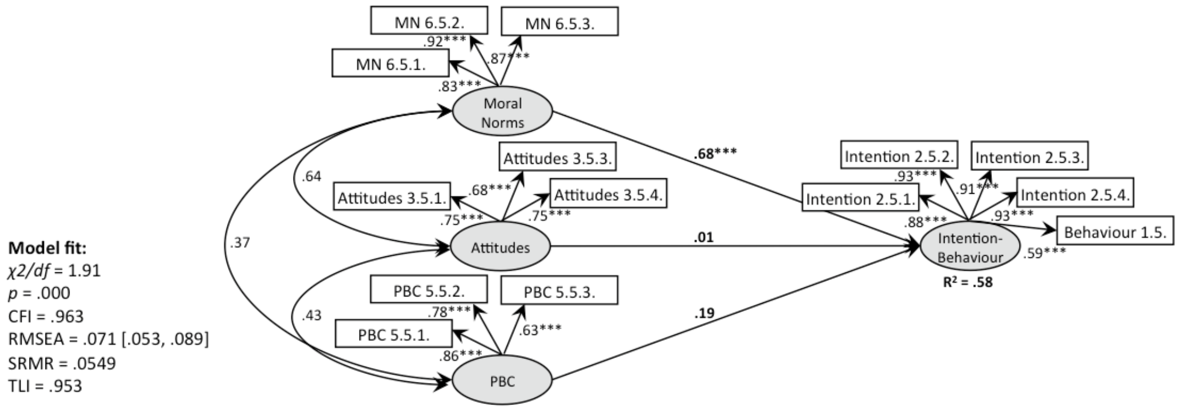

S7 Fig E. SEM, behaviour 5 (transport use): TPB with moral norms as additional predictor (adjusted Model 2).

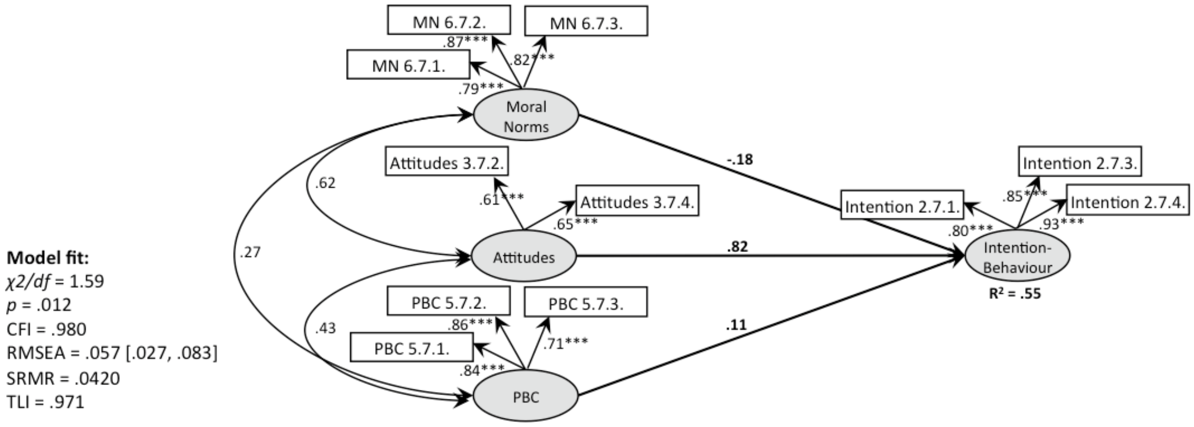

S7 Fig F. SEM, behaviour 7 (local products): TPB with moral norms as additional predictor (adjusted Model 2).

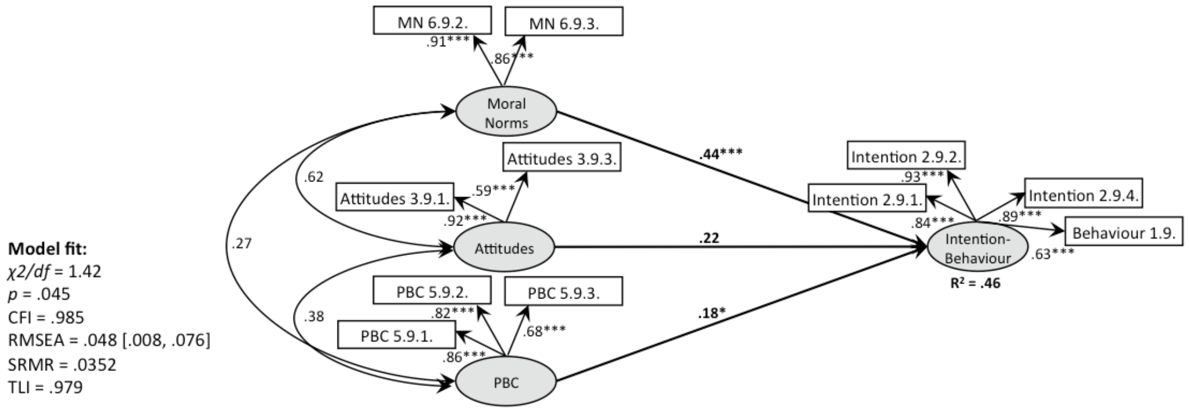

S7 Fig G. SEM, behaviour 9 (plastic bags): TPB with moral norms as additional predictor (adjusted Model 2).
