# Supplementary material for: The Theory of Planned Behaviour doesn’t reveal ’attitude-behaviour’ gap? Contrasting the effects of moral norms vs. idealism and relativism in predicting pro-environmental behaviours
Source: PLoS One. 2023 Nov 27;18(11):e0290818. doi: 10.1371/journal.pone.0290818 (PMC10681191; doi:10.1371/journal.pone.0290818)
Supplement: S9 Fig — (PDF) [file pone.0290818.s009.pdf]

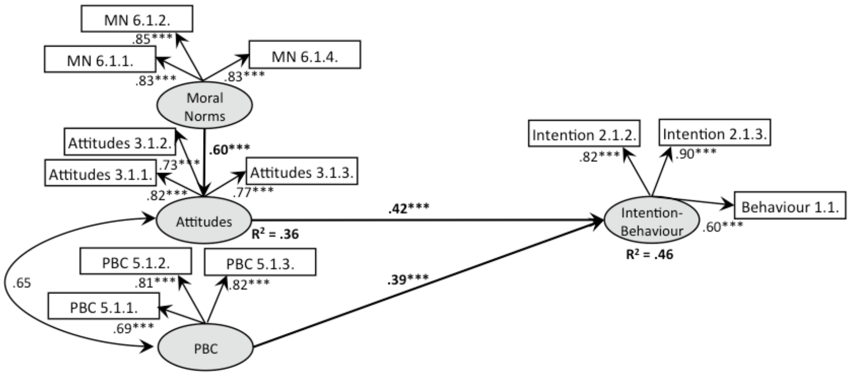

Model fit:
χ2/df = 2.19
p = .000
CFI = .947
RMSEA = .081 [.061, .102]
SRMR = .0750
TLI = .929

S9 Fig A. SEM, behaviour 1 (recycling): TPB with moral norms as predictor of attitudes (adjusted Model 4).

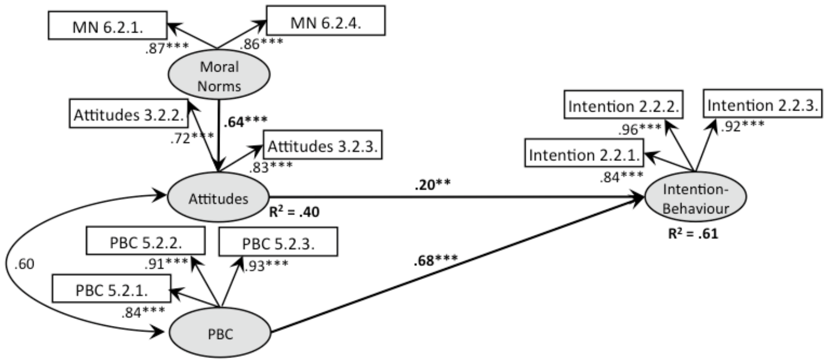

Model fit:
χ2/df = 2.34
p = .000
CFI = .962
RMSEA = .098 [.074, .124]
SRMR = .0638
TLI = .944

S9 Fig B. SEM, behaviour 2 (composting): TPB with moral norms as predictor of attitudes (adjusted Model 4).

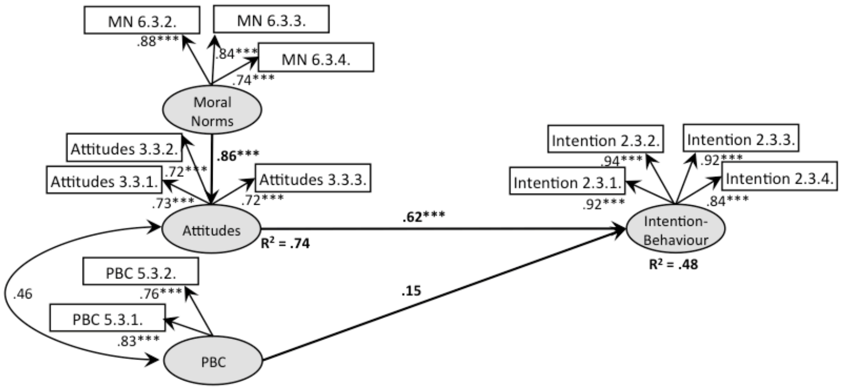

Model fit:
χ2/df = 2.06
p = .000
CFI = .965
RMSEA = .077 [.055, .098]
SRMR = .0544
TLI = .953

S9 Fig C. SEM, behaviour 3 (el. devices): TPB with moral norms as predictor of attitudes (adjusted Model 4).

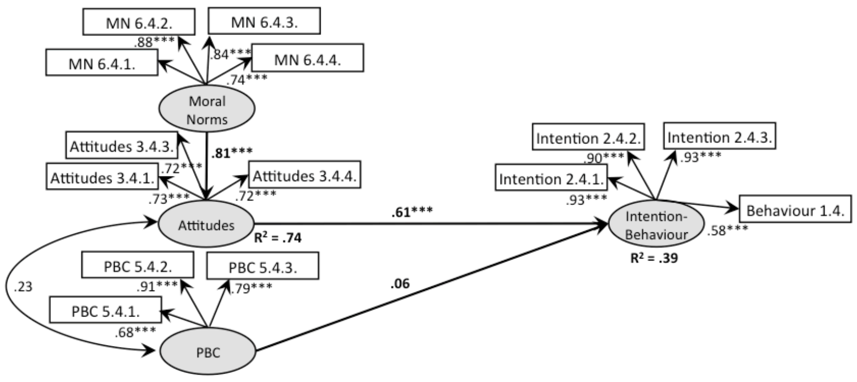

Model fit:
χ2/df = 2.20
p = .000
CFI = .947
RMSEA = .082 [.065, .099]
SRMR = .0761
TLI = .934

S9 Fig D. SEM, behaviour 4 (air cond.): TPB with moral norms as predictor of attitudes (adjusted Model 4).

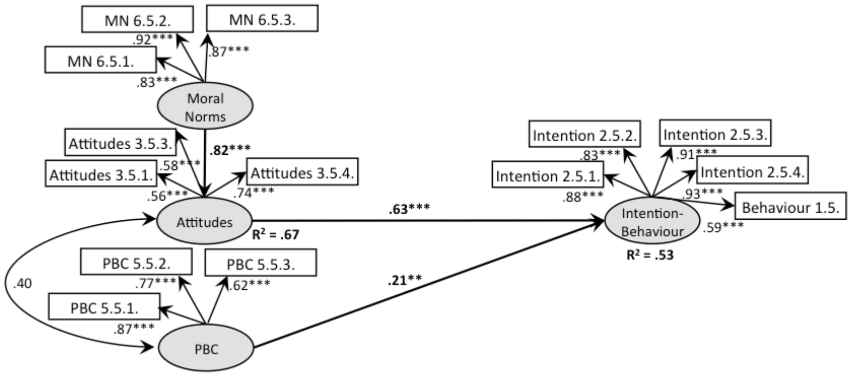

Model fit:
χ2/df = 2.55
p = .000
CFI = .936
RMSEA = .093 [.076, .109]
SRMR = .0770
TLI = .920

S9 Fig E. SEM, behaviour 5 (transport use): TPB with moral norms as predictor of attitudes (adjusted Model 4).

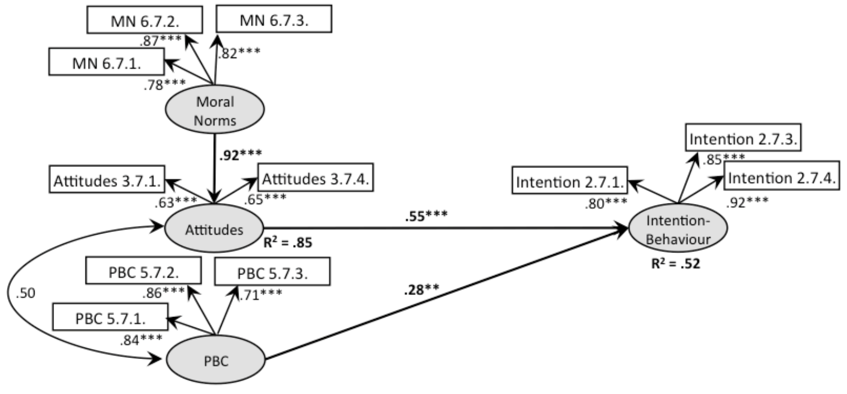

Model fit:
χ2/df = 1.69
p = .004
CFI = .975
RMSEA = .062 [.035, .087]
SRMR = .0502
TLI = .966

S9 Fig F. SEM, behaviour 7 (local products): TPB with moral norms as predictor of attitudes (adjusted Model 4).

Model fit:
χ2/df = 2.04
p = .000
CFI = .962
RMSEA = .076 [.052, .099]
SRMR = .0663
TLI = .948

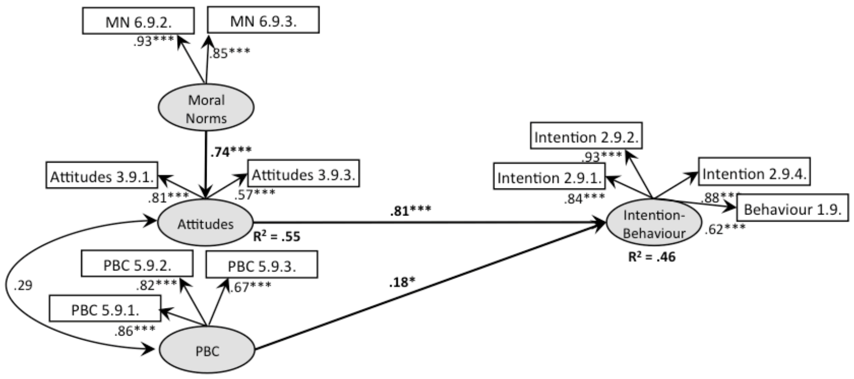

S9 Fig G. SEM, behaviour 9 (plastic bags): TPB with moral norms as predictor of attitudes (adjusted Model 4).
