# Supplementary material for: The Theory of Planned Behaviour doesn’t reveal ’attitude-behaviour’ gap? Contrasting the effects of moral norms vs. idealism and relativism in predicting pro-environmental behaviours
Source: PLoS One. 2023 Nov 27;18(11):e0290818. doi: 10.1371/journal.pone.0290818 (PMC10681191; doi:10.1371/journal.pone.0290818)
Supplement: S1 Table — (PDF) [file pone.0290818.s011.pdf]

S1 Table A. Behaviour 1 – Recycling: Descriptives and Shapiro-Wilk test results for normality assumptions.

Variable	*M*	*SD*	Statistic	df	*p*	*Skewness*	*Kurtosis*
Behaviour 1: Recycling							
Behaviour_1_1	4.80	1.59	.914	181	<.001	-.575	-.522
Intention_2_1_1	5.91	1.09	.818	181	<.001	-1.305	2.032
Intention_2_1_2	5.81	1.28	.798	181	<.001	-1.452	2.001
Intention_2_1_3	5.81	1.30	.786	181	<.001	-1.584	2.565
Intention_2_1_4	5.78	1.23	.814	181	<.001	-1.317	1.568
Attitudes_3_1_1	5.86	1.08	.806	181	<.001	-1.439	2.998
Attitudes_3_1_2	6.07	1.04	.800	181	<.001	-1.328	2.095
Attitudes_3_1_3	5.75	1.26	.813	181	<.001	-1.392	1.915
Attitudes_3_1_4	5.76	1.17	.838	181	<.001	-1.252	1.917
Subjective_Norm_4_1_1	4.78	1.57	.908	181	<.001	-.658	-.325
Subjective_Norm_4_1_2	4.90	1.54	.894	181	<.001	-.716	-.356
Subjective_Norm_4_1_3	5.56	1.28	.837	181	<.001	-1.299	1.912
Subjective_Norm_4_1_4	5.96	1.38	.737	181	<.001	-1.763	2.901
PBC_5_1_1	5.56	0.99	.868	181	<.001	-.816	.934
PBC_5_1_2	5.63	1.38	.811	181	<.001	-1.465	2.129
PBC_5_1_3	5.95	0.97	.838	181	<.001	-1.033	1.420
PBC_5_1_4	5.20	1.54	.892	181	<.001	-.772	-.134
Moral_Norms_6_1_1	5.52	1.44	.820	181	<.001	-1.349	1.469
Moral_Norms_6_1_2	5.38	1.55	.844	181	<.001	-1.077	.385
Moral_Norms_6_1_3	5.31	1.56	.820	181	<.001	-1.259	.909
Moral_Norms_6_1_4	5.78	1.22	.822	181	<.001	-1.374	2.198
Relativism_7_1_1	3.54	1.79	.170	181	<.001	.369	-.967
Relativism_7_1_2	4.81	1.41	.240	181	<.001	-.559	-.293
Relativism_7_1_3	4.51	1.53	.172	181	<.001	-.287	-.590
Relativism_7_1_4	4.64	1.57	.178	181	<.001	-.438	-.492
Relativism_7_1_5	4.39	1.54	.189	181	<.001	-.313	-.656
Relativism_7_1_6	4.36	1.55	.202	181	<.001	-.319	-.838
Relativism_7_1_7	4.55	1.51	.196	181	<.001	-.393	-.491
Relativism_7_1_8	4.84	1.39	.170	181	<.001	-.498	-.084
Relativism_7_1_9	4.59	1.60	.199	181	<.001	-.461	-.569
Relativism_7_1_10	4.88	1.48	.255	181	<.001	-.901	.376
Idealism_7_2_1	5.47	1.27	.220	181	<.001	-1.136	1.531
Idealism_7_2_2	4.85	1.49	.210	181	<.001	-.506	-.513
Idealism_7_2_3	4.97	1.45	.204	181	<.001	-.597	-.247
Idealism_7_2_4	5.66	1.23	.233	181	<.001	-1.088	1.488
Idealism_7_2_5	5.54	1.26	.211	181	<.001	-1.144	1.799
Idealism_7_2_6	5.46	1.28	.194	181	<.001	-.975	1.153
Idealism_7_2_7	4.30	1.64	.146	181	<.001	-.116	-.892
Idealism_7_2_8	5.56	1.22	.261	181	<.001	-1.155	1.658
Idealism_7_2_9	4.87	1.61	.168	181	<.001	-.569	-.391
Idealism_7_2_10	4.95	1.26	.179	181	<.001	-.516	.380

S1 Table B. Behaviour 2 – Composting: Descriptives and Shapiro-Wilk test results for normality assumptions.

Variable	*M*	*SD*	Statistic	df	*p*	*Skewness*	*Kurtosis*
Behaviour 2: Composting							
Behaviour_1_2_Composting	3.15	2.15	.843	181	<.001	.484	-1.217
Intention_2_2_1	4.70	1.83	.908	181	<.001	-.454	-.902
Intention_2_2_2	4.42	2.04	.892	181	<.001	-.265	-.1.317
Intention_2_2_3	4.44	2.11	.879	181	<.001	-.190	-1.448
Intention_2_2_4	4.28	2.12	.887	181	<.001	-.156	-1.428
Attitudes_3_2_1	5.25	1.46	.860	181	<.001	-1.104	.860
Attitudes_3_2_2	5.51	1.49	.825	181	<.001	-1.220	.849
Attitudes_3_2_3	5.14	1.63	.878	181	<.001	-.806	-.278
Attitudes_3_2_4	5.10	1.51	.909	181	<.001	-.523	-.437
Subjective_Norm_4_2_1	3.83	2.06	.895	181	<.001	.121	-1.402
Subjective_Norm_4_2_2	3.87	1.91	.912	181	<.001	.073	-1.294
Subjective_Norm_4_2_3	4.50	1.83	.917	181	<.001	-.303	-1.073
Subjective_Norm_4_2_4	5.62	1.53	.807	181	<.001	-1.294	1.004
PBC_5_2_1	4.92	1.60	.900	181	<.001	-.633	-.526
PBC_5_2_2	4.67	1.90	.896	181	<.001	-.462	-1.029
PBC_5_2_3	4.68	1.92	.891	181	<.001	-.544	-.924
PBC_5_2_4	4.56	1.81	.917	181	<.001	-.341	-1.018
Moral_Norms_6_2_1	4.57	1.67	.922	181	<.001	-.386	-.887
Moral_Norms_6_2_2	4.18	1.97	.907	181	<.001	-.166	-1.310
Moral_Norms_6_2_3	4.08	1.93	.909	181	<.001	-.166	-1.301
Moral_Norms_6_2_4	4.96	1.61	.909	181	<.001	-.692	-.151

S1 Table C. Behaviour 3 – Electronic Devices: Descriptives and Shapiro-Wilk test results for normality assumptions.

Variable	*M*	*SD*	Statistic	df	*p*	*Skewness*	*Kurtosis*
Behaviour 3: El. Devices							
Behaviour_1_3_El. Devices	4.31	1.53	.948	181	<.001	-.148	-.689
Intention_2_3_1	5.54	1.52	.837	181	<.001	-1.182	.890
Intention_2_3_2	5.41	1.61	.843	181	<.001	-1.073	.451
Intention_2_3_3	5.40	1.53	.849	181	<.001	-1.132	.826
Intention_2_3_4	5.31	1.67	.854	181	<.001	-.981	.148
Attitudes_3_3_1	5.56	1.35	.854	181	<.001	-1.178	1.513
Attitudes_3_3_2	3.88	2.03	.902	181	<.001	.123	1.369
Attitudes_3_3_3	5.35	1.47	.867	181	<.001	-1.034	.598
Attitudes_3_3_4	5.49	1.34	.856	181	<.001	-1.176	1.591
Subjective_Norm_4_3_1	4.40	1.81	.920	181	<.001	-.341	-.999
Subjective_Norm_4_3_2	4.44	1.83	.913	181	<.001	-.351	-1.046
Subjective_Norm_4_3_3	5.09	1.59	.880	181	<.001	-.932	.281
Subjective_Norm_4_3_4	5.87	1.39	.779	181	<.001	-1.502	2.004
PBC_5_3_1	5.88	1.20	.820	181	<.001	-1.303	1.820
PBC_5_3_2	5.95	1.19	.806	181	<.001	-1.346	1.921
PBC_5_3_3	6.16	1.19	.711	181	<.001	-1.960	4.328
PBC_5_3_4	5.11	1.55	.894	181	<.001	-.799	-.069
Moral_Norms_6_3_1	5.05	1.58	.897	181	<.001	-.777	-.111
Moral_Norms_6_3_2	4.86	1.69	.905	181	<.001	-.581	-.653
Moral_Norms_6_3_3	4.93	1.70	.892	181	<.001	-.733	-.406
Moral_Norms_6_3_4	5.53	1.39	.853	181	<.001	-1.187	1.419

S1 Table D. Behaviour 4 – Air Conditioning: Descriptives and Shapiro-Wilk test results for normality assumptions.

Variable	*M*	*SD*	Statistic	df	*p*	*Skewness*	*Kurtosis*
Behaviour 4: Air Cond.							
Behaviour_1_4_Air Cond.	4.35	1.54	.149	181	<.001	-.334	-.554
Intention_2_4_1	5.28	1.61	.204	181	<.001	-.927	.109
Intention_2_4_2	5.13	1.71	.193	181	<.001	-.859	-.123
Intention_2_4_3	5.10	1.70	.195	181	<.001	-.905	.070
Intention_2_4_4	4.98	1.73	.219	181	<.001	-.864	-.167
Attitudes_3_4_1	5.59	1.35	.254	181	<.001	-1.317	1.731
Attitudes_3_4_2	3.77	2.01	.170	181	<.001	.123	-1.376
Attitudes_3_4_3	5.31	1.47	.216	181	<.001	-1.058	.701
Attitudes_3_4_4	5.46	1.27	.217	181	<.001	-1.046	1.346
Subjective_Norm_4_4_1	4.14	1.74	.148	181	<.001	-1.27	-1.148
Subjective_Norm_4_4_2	4.13	1.82	.165	181	<.001	-.180	-1.171
Subjective_Norm_4_4_3	5.07	1.55	.211	181	<.001	-.833	.015
Subjective_Norm_4_4_4	5.84	1.37	.226	181	<.001	-1.422	1.876
PBC_5_4_1	5.80	1.07	.239	181	<.001	-.827	.452
PBC_5_4_2	5.98	1.09	.243	181	<.001	-1.127	.994
PBC_5_4_3	6.04	1.10	.246	181	<.001	-1.373	2.064
PBC_5_4_4	4.98	1.62	.213	181	<.001	-.803	-.061
Moral_Norms_6_4_1	4.94	1.51	.201	181	<.001	-.562	-.418
Moral_Norms_6_4_2	4.85	1.69	.220	181	<.001	-.730	-.417
Moral_Norms_6_4_3	4.81	1.74	.207	181	<.001	-.668	-.556
Moral_Norms_6_4_4	5.31	1.47	.233	181	<.001	-1.038	.757

S1 Table E. Behaviour 5 – Transport Use: Descriptives and Shapiro-Wilk test results for normality assumptions.

Variable	*M*	*SD*	Statistic	df	*p*	*Skewness*	*Kurtosis*
Behaviour 5: Transp. Use							
Behaviour_1_5_Transp. Use	3.65	1.76	.125	181	<.001	.073	-.946
Intention_2_5_1	4.85	1.67	.183	181	<.001	-.523	-.684
Intention_2_5_2	4.67	1.83	.191	181	<.001	-.551	-.691
Intention_2_5_3	4.61	1.84	.185	181	<.001	-.479	-.804
Intention_2_5_4	4.62	1.82	.185	181	<.001	-.507	-.834
Attitudes_3_5_1	5.72	1.31	.274	181	<.001	-1.416	2.028
Attitudes_3_5_2	3.92	1.97	.166	181	<.001	.003	-1.272
Attitudes_3_5_3	5.50	1.48	.245	181	<.001	-1.195	1.023
Attitudes_3_5_4	5.33	1.39	.199	181	<.001	-.787	.203
Subjective_Norm_4_5_1	3.88	1.97	.184	181	<.001	.185	-1.331
Subjective_Norm_4_5_2	3.92	1.87	.147	181	<.001	-.011	-1.220
Subjective_Norm_4_5_3	5.18	1.63	.213	181	<.001	-.803	-.126
Subjective_Norm_4_5_4	5.62	1.62	.250	181	<.001	-1.215	.579
PBC_5_5_1	5.34	1.42	.236	181	<.001	-1.056	.700
PBC_5_5_2	5.44	1.37	.233	181	<.001	-1.164	1.336
PBC_5_5_3	5.72	1.29	.237	181	<.001	-1.084	.750
PBC_5_5_4	4.73	1.69	.182	181	<.001	-.563	-.643
Moral_Norms_6_5_1	4.60	1.68	.181	181	<.001	-.426	-.842
Moral_Norms_6_5_2	4.34	1.84	.165	181	<.001	-.210	-1.163
Moral_Norms_6_5_3	4.34	1.88	.195	181	<.001	-.318	-1.139
Moral_Norms_6_5_4	5.15	1.50	.201	181	<.001	-.932	.553

S1 Table F. Behaviour 7 – Local Products: Descriptives and Shapiro-Wilk test results for normality assumptions.

Variable	*M*	*SD*	Statistic	df	*p*	*Skewness*	*Kurtosis*
Behaviour 7: Local Prod.							
Behaviour_1_7_Local Prod.	3.94	1.53	.151	181	<.001	.168	-.576
Intention_2_7_1	5.39	1.30	.205	181	<.001	-1.021	1.155
Intention_2_7_2	5.17	1.42	.214	181	<.001	-.847	.598
Intention_2_7_3	5.23	1.46	.216	181	<.001	-.912	.574
Intention_2_7_4	5.23	1.33	.193	181	<.001	-.768	.527
Attitudes_3_7_1	5.17	1.49	.233	181	<.001	-1.047	.899
Attitudes_3_7_2	4.34	1.81	.172	181	<.001	-.425	-.884
Attitudes_3_7_3	4.99	1.63	.204	181	<.001	-.893	.139
Attitudes_3_7_4	5.39	1.44	.227	181	<.001	-1.170	1.342
Subjective_Norm_4_7_1	4.49	1.69	.182	181	<.001	-.462	-.681
Subjective_Norm_4_7_2	4.43	1.66	.181	181	<.001	-.340	-.865
Subjective_Norm_4_7_3	5.07	1.55	.222	181	<.001	-.846	.280
Subjective_Norm_4_7_4	5.69	1.40	.245	181	<.001	-1.400	1.929
PBC_5_7_1	5.38	1.33	.228	181	<.001	-1.080	1.120
PBC_5_7_2	5.43	1.45	.208	181	<.001	-1.047	.620
PBC_5_7_3	5.75	1.16	.205	181	<.001	-.871	.401
PBC_5_7_4	5.24	1.26	.197	181	<.001	-.642	.040
Moral_Norms_6_7_1	4.80	1.63	.173	181	<.001	-.599	-.467
Moral_Norms_6_7_2	4.48	1.75	.192	181	<.001	-.340	-.920
Moral_Norms_6_7_3	4.65	1.74	.166	181	<.001	-.461	-.772
Moral_Norms_6_7_4	5.22	1.46	.212	181	<.001	-.846	.353

S1 Table G. Behaviour 9 – Plastic Bags: Descriptives and Shapiro-Wilk test results for normality assumptions.

Variable	*M*	*SD*	Statistic	df	*p*	*Skewness*	*Kurtosis*
Behaviour 9: Local Prod.							
Behaviour_1_9	4.27	1.82	.142	181	<.001	-.325	-.878
Intention_2_9_1	5.49	1.40	.217	181	<.001	-1.028	.702
Intention_2_9_2	5.35	1.56	.231	181	<.001	-1.041	.397
Intention_2_9_3	5.23	1.64	.206	181	<.001	-.894	.007
Intention_2_9_4	5.19	1.67	.222	181	<.001	-.969	.135
Attitudes_3_9_1	5.76	1.28	.581	181	<.001	-1.361	1.831
Attitudes_3_9_2	5.93	1.29	.251	181	<.001	-1.603	2.718
Attitudes_3_9_3	5.55	1.42	.243	181	<.001	-1.177	1.114
Attitudes_3_9_4	5.57	1.38	.241	181	<.001	-1.240	1.583
Subjective_Norm_4_9_1	4.66	1.71	.181	181	<.001	-.472	-.796
Subjective_Norm_4_9_2	4.50	1.80	.184	181	<.001	-.434	-.955
Subjective_Norm_4_9_3	5.24	1.50	.198	181	<.001	-.921	.423
Subjective_Norm_4_9_4	5.85	1.28	.248	181	<.001	-1.372	1.913
PBC_5_9_1	6.02	1.10	.251	181	<.001	-1.460	2.831
PBC_5_9_2	5.90	1.09	.238	181	<.001	-1.222	2.283
PBC_5_9_3	6.05	1.19	.284	181	<.001	-1.861	4.124
PBC_5_9_4	5.07	1.60	.211	181	<.001	-.824	-.067
Moral_Norms_6_9_1	5.21	1.55	.215	181	<.001	-.932	.172
Moral_Norms_6_9_2	5.10	1.72	.208	181	<.001	-.896	-.084
Moral_Norms_6_9_3	4.98	1.78	.226	181	<.001	-.868	-.303
Moral_Norms_6_9_4	5.48	1.52	.260	181	<.001	-1.299	1.304
