# Supplementary material for: The Theory of Planned Behaviour doesn’t reveal ’attitude-behaviour’ gap? Contrasting the effects of moral norms vs. idealism and relativism in predicting pro-environmental behaviours
Source: PLoS One. 2023 Nov 27;18(11):e0290818. doi: 10.1371/journal.pone.0290818 (PMC10681191; doi:10.1371/journal.pone.0290818)
Supplement: S2 Table — (PDF) [file pone.0290818.s012.pdf]

S2 Table A. The results of exploratory factor analysis (Model 1).

Factors and items	Factor loadings	Communalities
**Behaviour 1 - Recycling**		
Factor 1: Behaviour-Intention. *Cronbach's α = .88, Eigenvalue = 6.625, Variance = 44.17%*		
1.1. Recycle newspapers, plastics, cans and glass	.685	.526
2.1.1. I am willing to recycle newspapers, plastics, cans and glass	.672	.704
2.1.2. I intend to recycle newspapers, plastics, cans and glass	.945	.855
2.1.3. I plan to recycle newspapers, plastics, cans and glass	.891	.760
2.1.4. I will recycle newspapers, plastics, cans and glass	.849	.797
Factor 2: Attitudes. *Cronbach's α = .86, Eigenvalue = 1.602, Variance = 10.68%*		
3.1.1. I believe that my recycling behavior will help reduce pollution	.869	.768
3.1.2. I believe that my recycling behavior will help reduce wasteful use of landfills)	.798	.688
3.1.3. I believe that my recycling behavior will help conserve natural resources	.838	.688
3.1.4. I feel good about myself when I recycle	.793	.728
Factor 3: Subjective Norm. *Cronbach's α = .74, Eigenvalue = 1.291, Variance = 8.61%*		
4.1.1. My friends expect me to recycle recyclables	.806	.766
4.1.2. My classmates/colleagues expect me to recycle recyclables	.918	.805
4.1.3. Media influences me to recycle recyclables	.551	.432
Factor 4: Perceived Behavioural Control. *Cronbach's α = .81, Eigenvalue = 1.214, Variance = 8.10%*		
5.1.1. I know what items can be recycled	.815	.858
5.1.2. I know where I can recycle newspapers, plastics, cans and glass	.877	.806
5.1.3. I know how to recycle my recyclables	.803	.704
Total variance = 71.55%		
KMO = .875		
χ^2 = 1546.706		
df = 105		
Sig. < .001		

S2 Table B. The results of exploratory factor analysis for original TPB (Model 1).

Factors and items		Factor loadings	Communalities
**Behaviour 2 - Composting**			
Factor 1: Behaviour-Intention.	*Cronbach's α = .95, Eigenvalue = 6.784, Variance = 52.18%*		
2.2.1. I am willing to compost kitchen waste		.714	.782
2.2.2. I intend to compost kitchen waste		.965	.921
2.2.3. I plan to compost kitchen waste		.948	.889
2.2.4. I will compost kitchen waste		.969	.909
Factor 2: Attitudes.	*Cronbach's α = .85, Eigenvalue = 1.674, Variance = 12.88%*		
3.2.1. I believe that my composting kitchen waste items will help reduce pollution		.812	.751
3.2.2. I believe that my composting kitchen waste items will help reduce wasteful use of landfills		.926	.773
3.2.3. I believe that my composting kitchen waste items will help conserve natural resources		.902	.762
3.2.4. I feel good about myself when I compost kitchen waste items		.580	.635
Factor 3: Subjective Norm.	*Cronbach's α = .62, Eigenvalue = 1.166, Variance = 8.97%*		
4.2.3. Media influences me to compost compostable items		.721	.709
4.2.4. Environmental groups influence me to compost compostable items		.913	.792
Factor 4: Perceived Behavioural Control.	*Cronbach's α = .92, Eigenvalue = .901, Variance = 6.93%*		
5.2.1. I know what kitchen waste items can be composted		.969	.862
5.2.2. I know I can compost kitchen waste		.809	.860
5.2.3. I know how to compost my compostable items		.827	.880
Total variance = 80.96%			
KMO = .890			
χ^2 = 1888.201			
df = 78			
Sig. = .000			

S2 Table C. The results of exploratory factor analysis for original TPB (Model 1).

**Factors and items**		Factor loadings	Communalities
**Behaviour 3 – Electronic Devices**			
Factor 1: Behaviour-Intention.	*Cronbach's α = .91, Eigenvalue = 5.535, Variance = 42.58%*		
1.3. Turn off or unplug electronic devises when not need		.602	.414
2.3.1. I am willing to turn off or unplug electronic devises when not need		.880	.794
2.3.2. I intend to turn off or unplug electronic devises when not need		.915	.871
2.3.3. I plan to turn off or unplug electronic devises when not need		.917	.876
2.3.4. I will turn off or unplug electronic devises when not need		.882	.851
Factor 2: Attitudes.	*Cronbach's α = .79, Eigenvalue = 1.946, Variance = 14.97%*		
3.3.1. I believe that turning off or unplugging electronic devises when not need will help reduce pollution		.833	.713
3.3.3. I believe that turning off or unplugging electronic devises when not need will help conserve natural resources		.897	.755
3.3.4. I feel good about myself when I turn off or unplug electronic devises when not need		.733	.685
Factor 3: Subjective Norm.	*Cronbach's α = .62, Eigenvalue = 1.175, Variance = 9.04%*		
4.3.3. Media influences me to turn off or unplug electronic devices when not in need		.982	.809
4.3.4. Environmental groups influence me to turn off or unplug electronic devices when not in need		.741	.734
Factor 4: Perceived Behavioural Control.	*Cronbach's α = .82, Eigenvalue = 1.046, Variance = 8.05%*		
5.3.1. I know what electronic items can be turned off or unplugged when not in need		.784	.654
5.3.2. I know where I can turn off or unplug all of my electronic devices when not in need		.901	.802
5.3.3. I know how to turn off or unplug all of my electronic devices when not in need		.854	.745
Total variance = 74.64%			
KMO = .862			
χ^2 = 1376.129			
df = 78			
Sig. = .001			

S2 Table D. The results of exploratory factor analysis for original TPB (Model 1).

Factors and items		Factor loadings	Communalities
**Behaviour 4 – Air Conditioning**			
Factor 1: Behaviour-Intention.	*Cronbach's α = .93, Eigenvalue = 5.249, Variance = 40.38%*		
1.4. Reduce air conditioning		.655	.494
2.4.1. I am willing to reduce air conditioning when not need		.911	.862
2.4.2. I intend to reduce air conditioning when not need		.931	.875
2.4.3. I plan to reduce air conditioning when not need		.973	.871
2.4.4. I will reduce air conditioning when not need		.929	.867
Factor 2: Attitudes.	*Cronbach's α = .80, Eigenvalue = 1.424, Variance = 10.95%*		
3.4.1. I believe that reducing air conditioning when not in need will help reduce pollution		.909	.794
3.4.3. I believe that reducing air conditioning when not in need will help conserve natural resources		.926	.803
3.4.4. I feel good about myself when I reduce air conditioning that's not in need		.596	.596
Factor 3: Subjective Norm.	*Cronbach's α = .73, Eigenvalue = 1.217, Variance = 9.36%*		
4.4.3. Media influences me to reduce air conditioning when not in need		.879	.815
4.4.4. Environmental groups influence me to reduce air conditioning when not in need		.874	.810
Factor 4: Perceived Behavioural Control.	*Cronbach's α = .83, Eigenvalue = 2.175, Variance = 16.73%*		
5.4.1. I know what air conditioning systems can be reduced when not in need		.791	.685
5.4.2. I know where I can reduce air conditioning when not in need		.884	.813
5.4.3. I know how to reduce air conditioning when not in need		.885	.779
Total variance = 77.42%			
KMO = .834			
χ^2 = 1501.282			
df = 78			
Sig. < .001			

S2 Table E. The results of exploratory factor analysis for original TPB (Model 1).

Factors and items	Factor loadings	Communalities
**Behaviour 5 – Transport Use**		
Factor 1: Behaviour-Intention. Cronbach's α = .93, Eigenvalue = 5.809, Variance = 41.48%		
1.5. Reduce driving, and walk, bike or use public transportation	.601	.480
2.5.1. I am willing to reduce driving, and instead walk, bike or use public transportation	.919	.820
2.5.2. I intend to reduce driving, and instead walk, bike or use public transportation	.976	.884
2.5.3. I plan to reduce driving, and instead walk, bike or use public	.961	.850
2.5.4. I will reduce driving, and instead walk, bike or use public transportation	.966	.879
Factor 2: Attitudes. Cronbach's α = .76, Eigenvalue = 1.411, Variance = 10.08%		
3.5.1. I believe that reducing driving, and instead walking, biking or using public transportation will help reduce pollution	.918	.791
3.5.3. I believe that reducing driving, and instead walking, biking or using public transportation will help conserve natural resources	.844	.703
3.5.4. I feel good about myself when I reduce driving, and instead walk, bike or use public transportation	.567	.644
Factor 3: Subjective Norm. Cronbach's α = .71, Eigenvalue = 1.215, Variance = 8.68%		
4.5.1. My friends expect me to reduce driving, and walk, bike or use public transportation	.666	.704
4.5.2. My classmates/colleagues expect me to reduce driving, and walk, bike or use public transportation	.844	.729
4.5.3. Media influences me to reduce driving, and walk, bike or use public transportation	.816	.533
Factor 4: Perceived Behavioural Control. Cronbach's α = .80, Eigenvalue =1.800, Variance = 12.86%		
5.5.1. I know what route I can take in an attempt to reduce driving and instead walk, bike, or take public transportation	.823	.775
5.5.2. I know where I can reasonably travel to if I choose to reduce driving and instead walk, bike, or take public transportation	.873	.758
5.5.3. I know how to reduce driving and instead walk, bike, or take public transportation	.836	.684
Total variance = 73.10%		
KMO = .842		
χ^2 = 1528.105		
df = 91		
Sig. < .001		

S2 Table F. The results of exploratory factor analysis for original TPB (Model 1).

Factors and items		Factor loadings	Communalities
**Behaviour 7 – Local products**			
Factor 1: Behaviour-Intention.	*Cronbach's α = .92, Eigenvalue = 5.830, Variance = 44.85%*		
2.7.1. I am willing to buy local products or locally produced foods		.875	.776
2.7.2. I intend to buy local products or locally produced foods		.871	.857
2.7.3. I plan to buy local products or locally produced foods		.915	.816
2.7.4. I will buy local products or locally produced foods		.928	.845
Factor 2: Attitudes.	*Cronbach's α = .83, Eigenvalue = 1.725, Variance = 13.27%*		
3.7.1. I believe that buying local products or locally produced foods will help reduce pollution		.713	.726
3.7.2. I believe that buying local products or locally produced foods will help reduce wasteful use of landfills		.954	.668
3.7.3. I believe that buying local products or locally produced foods will help conserve natural resources		.842	.805
3.7.4. I feel good about myself when I buy local products or locally produced foods		.564	.578
Factor 3: Subjective Norm.	*Cronbach's α = .66, Eigenvalue = 1.037, Variance = 7.98%*		
4.7.3. Media influences me to buy local products or locally produced foods		.881	.758
4.7.4. Environmental groups influence me to buy local products or locally produced foods		.819	.737
Factor 4: Perceived Behavioural Control.	*Cronbach's α = .85, Eigenvalue = 1.322, Variance = 10.17%*		
5.7.1. I know what I can do to be able to buy local products or locally produced foods		.827	.787
5.7.2. I know where I can buy local products or locally produced foods		.929	.844
5.7.3. I know how to buy local products or locally produced foods		.834	.717
Total variance = 76.26%			
KMO = .845			
χ^2 = 1409.387			
df = 78			
Sig. < .001			

S2 Table G. The results of exploratory factor analysis for original TPB (Model 1).

Factors and items		Factor loadings	Communalities
**Behaviour 7 – Plastic Bags**			
Factor 1: Behaviour-Intention.	*Cronbach's α = .92, Eigenvalue = 5.761, Variance = 41.15%*		
1.9. Reduce using plastic bags, or use own bag when shopping		.696	.573
2.9.1. I am willing to reduce using plastic bags, or use own bag when shopping		.858	.770
2.9.2. I intend to reduce using plastic bags, or use own bag when shopping		.908	.851
2.9.3. I plan to reduce using plastic bags, or use own bag when shopping		.906	.863
2.9.4. I will reduce using plastic bags, or use own bag when shopping		.935	.847
Factor 2: Attitudes.	*Cronbach's α = .81, Eigenvalue = 2.207, Variance = 15.77%*		
3.9.1. I believe that reducing the use of plastic bags, or using own bag when shopping will help reduce pollution		.837	.777
3.9.2. I believe that reducing the use plastic bags, or using own bag when shopping will help reduce wasteful use of landfills		.752	.639
3.9.3. I believe that reducing the use of plastic bags, or using own bag when shopping will help conserve natural resources		.814	.683
3.9.4. I feel good about myself when I reduce the use of plastic bags, or use own bag when shopping		.715	.659
Factor 3: Subjective Norm.	*Cronbach's α = .82, Eigenvalue = 1.072, Variance = 7.66%*		
4.9.1. My friends expect me to reduce using plastic bags, or use own bag when shopping		.819	.806
4.9.2. My classmates/colleagues expect me to reduce using plastic bags, or use own bag when shopping		.944	.866
Factor 4: Perceived Behavioural Control.	*Cronbach's α = .82, Eigenvalue = 1.534, Variance = 10.95%*		
5.9.1. I know what I can do to reduce using plastic bags, or use own bag when shopping		.909	.814
5.9.2. I know where I can reduce using plastic bags, or use own bag when shopping		.839	.753
5.9.3. I know how to reduce using plastic bags, or use own bag when shopping		.797	.670
Total variance = 75.53%			
KMO = .841			
$\chi^2 = 1556.725$			
df = 91			
Sig. < .000			
