# Supplementary material for: The Theory of Planned Behaviour doesn’t reveal ’attitude-behaviour’ gap? Contrasting the effects of moral norms vs. idealism and relativism in predicting pro-environmental behaviours
Source: PLoS One. 2023 Nov 27;18(11):e0290818. doi: 10.1371/journal.pone.0290818 (PMC10681191; doi:10.1371/journal.pone.0290818)
Supplement: S3 Table — (PDF) [file pone.0290818.s013.pdf]

S3 Table A. The results of exploratory factor analysis for original TPB without subjective norms (adjusted Model 1).

Factors and items	Factor loadings	Communalities
**Behaviour 1 - Recycling**		
Factor 1: Behaviour-Intention. *Cronbach's α = .88, Eigenvalue = 5.965, Variance = 49.71%*		
1.1. Recycle newspapers, plastics, cans and glass	.772	.510
2.1.1. I am willing to recycle newspapers, plastics, cans and glass	.597	.665
2.1.2. I intend to recycle newspapers, plastics, cans and glass	.923	.850
2.1.3. I plan to recycle newspapers, plastics, cans and glass	.880	.757
2.1.4. I will recycle newspapers, plastics, cans and glass	.850	.793
Factor 2: Attitudes. *Cronbach's α = .86, Eigenvalue = 1.453, Variance = 12.11%*		
3.1.1. I believe that my recycling behavior will help reduce pollution	.888	.775
3.1.2. I believe that my recycling behavior will help reduce wasteful use of landfills)	.801	.675
3.1.3. I believe that my recycling behavior will help conserve natural resources	.851	.694
3.1.4. I feel good about myself when I recycle	.814	.698
Factor 3: Perceived Behavioural Control. *Cronbach's α = .81, Eigenvalue = 1.209, Variance = 10.08%*		
5.1.1. I know what items can be recycled	.821	.682
5.1.2. I know where I can recycle newspapers, plastics, cans and glass	.895	.778
5.1.3. I know how to recycle my recyclables	.795	.749
Total variance = 71.89%		
KMO = .882		
$\chi^2 = 1295.529$		
df = 66		
Sig. < .001		

S3 Table B. The results of exploratory factor analysis for original TPB without subjective norms (adjusted Model 1).

Factors and items	Factor loadings	Communalities
**Behaviour 2 - Composting**		
Factor 1: Behaviour-Intention. Cronbach's α = .95, Eigenvalue = 6.436, Variance = 58.51%		
2.2.1. I am willing to compost kitchen waste	.739	.791
2.2.2. I intend to compost kitchen waste	.984	.921
2.2.3. I plan to compost kitchen waste	.964	.896
2.2.4. I will compost kitchen waste	.974	.907
Factor 2: Attitudes. Cronbach's α = .85, Eigenvalue = 1.652, Variance = 15.01%		
3.2.1. I believe that my composting kitchen waste items will help reduce pollution	.800	.724
3.2.2. I believe that my composting kitchen waste items will help reduce wasteful use of landfills	.917	.760
3.2.3. I believe that my composting kitchen waste items will help conserve natural resources	.909	.735
3.2.4. I feel good about myself when I compost kitchen waste items	.581	.617
Factor 3: Perceived Behavioural Control. Cronbach's α = .92, Eigenvalue = .881, Variance = 81.53%		
5.2.1. I know what kitchen waste items can be composted	.993	.861
5.2.2. I know I can compost kitchen waste	.863	.875
5.2.3. I know how to compost my compostable items	.847	.881
Total variance = 81.53%		
KMO = .894		
χ^2 = 1773.256		
df = 55		
Sig. = .000		

S3 Table C. The results of exploratory factor analysis for original TPB without subjective norms (adjusted Model 1).

Factors and items	Factor loadings	Communalities
**Behaviour 3 – Electronic Devices**		
Factor 1: Behaviour-Intention. *Cronbach's α = .91, Eigenvalue = 5.218, Variance = 47.44%*		
1.3. Turn off or unplug electronic devises when not need	.623	.393
2.3.1. I am willing to turn off or unplug electronic devises when not need	.888	.794
2.3.2. I intend to turn off or unplug electronic devises when not need	.932	.875
2.3.3. I plan to turn off or unplug electronic devises when not need	.935	.879
2.3.4. I will turn off or unplug electronic devises when not need	.888	.848
Factor 2: Attitudes. *Cronbach's α = .79, Eigenvalue = 1.123, Variance = 10.21%*		
3.3.1. I believe that turning off or unplugging electronic devises when not need will help reduce pollution	.835	.722
3.3.3. I believe that turning off or unplugging electronic devises when not need will help conserve natural resources	.903	.761
3.3.4. I feel good about myself when I turn off or unplug electronic devises when not need	.694	.662
Factor 3: Perceived Behavioural Control. *Cronbach's α = .82, Eigenvalue = 1.810, Variance = 16.45%*		
5.3.1. I know what electronic items can be turned off or unplugged when not in need	.791	.665
5.3.2. I know where I can turn off or unplug all of my electronic devices when not in need	.913	.811
5.3.3. I know how to turn off or unplug all of my electronic devices when not in need	.864	.740
Total variance = 74.10%		
KMO = .859		
χ^2 = 1274.795		
df = 55		
Sig. = .001		

S3 Table D. The results of exploratory factor analysis for original TPB without subjective norms (adjusted Model 1).

Factors and items		Factor loadings	Communalities
**Behaviour 4 – Air Conditioning**			
Factor 1: Behaviour-Intention.	*Cronbach's α = .93, Eigenvalue = 5.006, Variance = 45.51%*		
1.4. Reduce air conditioning		.638	.472
2.4.1. I am willing to reduce air conditioning when not need		.914	.862
2.4.2. I intend to reduce air conditioning when not need		.936	.875
2.4.3. I plan to reduce air conditioning when not need		.970	.871
2.4.4. I will reduce air conditioning when not need		.935	.864
Factor 2: Attitudes.	*Cronbach's α = .80, Eigenvalue = 2.085, Variance = 18.95%*		
3.4.1. I believe that reducing air conditioning when not in need will help reduce pollution		.912	.795
3.4.3. I believe that reducing air conditioning when not in need will help conserve natural resources		.925	.801
3.4.4. I feel good about myself when I reduce air conditioning that's not in need		.610	.601
Factor 3: Perceived Behavioural Control.	*Cronbach's α = .83, Eigenvalue = 1.321, Variance = 12.01%*		
5.4.1. I know what air conditioning systems can be reduced when not in need		.780	.671
5.4.2. I know where I can reduce air conditioning when not in need		.894	.816
5.4.3. I know how to reduce air conditioning when not in need		.907	.782
Total variance = 76.47%			
KMO = .839			
χ^2 = 1382.487			
df = 55			
Sig. < .001			

S3 Table E. The results of exploratory factor analysis for original TPB without subjective norms (adjusted Model 1).

Factors and items	Factor loadings	Communalities
**Behaviour 5 – Transport Use**		
Factor 1: Behaviour-Intention. *Cronbach's α = .93, Eigenvalue = 5.074, Variance = 46.13%*		
1.5. Reduce driving, and walk, bike or use public transportation	.675	.471
2.5.1. I am willing to reduce driving, and instead walk, bike or use public transportation	.889	.819
2.5.2. I intend to reduce driving, and instead walk, bike or use public transportation	.942	.873
2.5.3. I plan to reduce driving, and instead walk, bike or use public	.948	.857
2.5.4. I will reduce driving, and instead walk, bike or use public transportation	.933	.878
Factor 2: Attitudes. *Cronbach's α = .76, Eigenvalue = 1.357, Variance = 12.34%*		
3.5.1. I believe that reducing driving, and instead walking, biking or using public transportation will help reduce pollution	.911	.782
3.5.3. I believe that reducing driving, and instead walking, biking or using public transportation will help conserve natural resources	.845	.699
3.5.4. I feel good about myself when I reduce driving, and instead walk, bike or use public transportation	.625	.609
Factor 3: Perceived Behavioural Control. *Cronbach's α = .80, Eigenvalue =1.759, Variance = 16.00%*		
5.5.1. I know what route I can take in an attempt to reduce driving and instead walk, bike, or take public transportation	.821	.772
5.5.2. I know where I can reasonably travel to if I choose to reduce driving and instead walk, bike, or take public transportation	.871	.755
5.5.3. I know how to reduce driving and instead walk, bike, or take public transportation	.832	.677
Total variance = 74.59%		
KMO = .841		
χ^2 = 1273.725		
df = 55		
Sig. < .001		

S3 Table F. The results of exploratory factor analysis for original TPB without subjective norms (adjusted Model 1).

Factors and items		Factor loadings	Communalities
**Behaviour 7 – Local products**			
Factor 1: Behaviour-Intention.	*Cronbach's α = .92, Eigenvalue = 5.527, Variance = 50.25%*		
2.7.1. I am willing to buy local products or locally produced foods		.878	.757
2.7.2. I intend to buy local products or locally produced foods		.862	.845
2.7.3. I plan to buy local products or locally produced foods		.909	.811
2.7.4. I will buy local products or locally produced foods		.927	.845
Factor 2: Attitudes.	*Cronbach's α = .83, Eigenvalue = 1.674, Variance = 15.22%*		
3.7.1. I believe that buying local products or locally produced foods will help reduce pollution		.738	.730
3.7.2. I believe that buying local products or locally produced foods will help reduce wasteful use of landfills		.815	.649
3.7.3. I believe that buying local products or locally produced foods will help conserve natural resources		.967	.810
3.7.4. I feel good about myself when I buy local products or locally produced foods		.589	.583
Factor 3: Perceived Behavioural Control.	*Cronbach's α = .85, Eigenvalue = 1.167, Variance = 10.61%*		
5.7.1. I know what I can do to be able to buy local products or locally produced foods		.809	.772
5.7.2. I know where I can buy local products or locally produced foods		.935	.842
5.7.3. I know how to buy local products or locally produced foods		.848	.724
Total variance = 76.08%			
KMO = .848			
χ^2 = 1299.50			
df = 55			
Sig. < .001			

S3 Table G. The results of exploratory factor analysis for original TPB without subjective norms (adjusted Model 1).

Factors and items	Factor loadings	Communalities
**Behaviour 7 – Plastic Bags**		
Factor 1: Behaviour-Intention. Cronbach's α = .92, Eigenvalue = 5.441, Variance = 45.34%		
1.9. Reduce using plastic bags, or use own bag when shopping	.792	.547
2.9.1. I am willing to reduce using plastic bags, or use own bag when shopping	.843	.763
2.9.2. I intend to reduce using plastic bags, or use own bag when shopping	.894	.846
2.9.3. I plan to reduce using plastic bags, or use own bag when shopping	.900	.858
2.9.4. I will reduce using plastic bags, or use own bag when shopping	.923	.842
Factor 2: Attitudes. Cronbach's α = .81, Eigenvalue = 2.017, Variance = 16.81%		
3.9.1. I believe that reducing the use of plastic bags, or using own bag when shopping will help reduce pollution	.812	.754
3.9.2. I believe that reducing the use plastic bags, or using own bag when shopping will help reduce wasteful use of landfills	.719	.627
3.9.3. I believe that reducing the use of plastic bags, or using own bag when shopping will help conserve natural resources	.893	.669
3.9.4. I feel good about myself when I reduce the use of plastic bags, or use own bag when shopping	.716	.657
Factor 3: Perceived Behavioural Control. Cronbach's α = .82, Eigenvalue = 1.334, Variance = 11.11%		
5.9.1. I know what I can do to reduce using plastic bags, or use own bag when shopping	.904	.802
5.9.2. I know where I can reduce using plastic bags, or use own bag when shopping	.840	.746
5.9.3. I know how to reduce using plastic bags, or use own bag when shopping	.831	.680
Total variance = 73.26%		
KMO = .853		
χ^2 = 1368.943		
df = 66		
Sig. < .001		
