# Supplementary material for: The Theory of Planned Behaviour doesn’t reveal ’attitude-behaviour’ gap? Contrasting the effects of moral norms vs. idealism and relativism in predicting pro-environmental behaviours
Source: PLoS One. 2023 Nov 27;18(11):e0290818. doi: 10.1371/journal.pone.0290818 (PMC10681191; doi:10.1371/journal.pone.0290818)
Supplement: S4 Table — (PDF) [file pone.0290818.s014.pdf]

S4 Table A. The results of exploratory factor analysis for TPB extension with moral norms (Models 2, 4).

Factors and items		Factor loadings	Communalities
**Behaviour 1 – Recycling**			
Factor 1: Behaviour-Intention.	*Cronbach's α = .88, Eigenvalue = 7.745, Variance = 48.40%*		
1.1. Recycle newspapers, plastics, cans and glass		.775	.521
2.1.1. I am willing to recycle newspapers, plastics, cans and glass		.662	.703
2.1.2. I intend to recycle newspapers, plastics, cans and glass		.917	.851
2.1.3. I plan to recycle newspapers, plastics, cans and glass		.806	.753
2.1.4. I will recycle newspapers, plastics, cans and glass		.800	.787
Factor 2: Attitudes.	*Cronbach's α = .86, Eigenvalue = 1.618, Variance = 10.11%*		
3.1.1. I believe that my recycling behavior will help reduce pollution		.858	.771
3.1.2. I believe that my recycling behavior will help reduce wasteful use of landfills		.821	.700
3.1.3. I believe that my recycling behavior will help conserve natural resources		.861	.707
3.1.4. I feel good about myself when I recycle		.689	765
Factor 3: Perceived Behavioural Control.	*Cronbach's α = .81, Eigenvalue = 1.328, Variance = 8.30%*		
5.1.1. I know what items can be recycled		.818	.700
5.1.2. I know where I can recycle newspapers, plastics, cans and glass		.846	.754
5.1.3. I know how to recycle my recyclables		.765	.733
Factor 4: Moral Norms.	*Cronbach's α = .89, Eigenvalue = 1.191, Variance = 7.45%*		
6.1.1. It would be wrong of me **not** to recycle my recyclables		.767	.738
6.1.2. I would feel guilty if I did **not** recycle my recyclables		.806	.818
6.1.3. **Not** recycling goes against my principles		.834	.792
6.1.4. Everybody should share the responsibility to recycle recyclables		.911	.789
Total variance = 74.26%			
KMO = .902			
χ^2 = 1927.343			
df = 120			
Sig. = .000			

S4 Table B. The results of exploratory factor analysis for TPB extension with moral norms (Models 2, 4).

Factors and items		Factor loadings	Communalities
**Behaviour 2 – Composting**			
Factor 1: Behaviour-Intention.	*Cronbach's α = .95, Eigenvalue = 8.217, Variance = 58.69%*		
2.2.1. I am willing to compost kitchen waste		.743	.780
2.2.2. I intend to compost kitchen waste		.934	.924
2.2.3. I plan to compost kitchen waste		.923	.894
2.2.4. I will compost kitchen waste		.964	.914
Factor 2: Attitudes.	*Cronbach's α = .82, Eigenvalue = 1.611, Variance = 11.51%*		
3.2.1. I believe that my composting kitchen waste items will help reduce pollution		.828	.787
3.2.2. I believe that my composting kitchen waste items will help reduce wasteful use of landfills		.929	.814
3.2.3. I believe that my composting kitchen waste items will help conserve natural resources		.778	.723
Factor 3: Perceived Behavioural Control.	*Cronbach's α = .92, Eigenvalue = 1.171, Variance = 8.36%*		
5.2.1. I know what kitchen waste items can be composted		.991	.861
5.2.2. I know I can compost kitchen waste		.857	.875
5.2.3. I know how to compost my compostable items		.843	.880
Factor 4: Moral Norms.	*Cronbach's α = .92, Eigenvalue = .772, Variance = 5.52%*		
6.2.1. It would be wrong of me **not** to compost my compostable items		.894	.833
6.2.2. I would feel guilty if I did **not** compost my compostable items		.828	.885
6.2.3. **Not** composting goes against my principles		.682	.804
6.2.4. Everybody should share the responsibility to compost compostable items		.926	.798
Total variance = 84.08%			
KMO = .914			
χ^2 = 2431.095			
df = 91			
Sig. = .000			

S4 Table C. The results of exploratory factor analysis for TPB extension with moral norms (Models 2, 4).

Factors and items		Factor loadings	Communalities
**Behaviour 3 – Electronic Devices**			
Factor 1: Behaviour-Intention.	*Cronbach's α = .95, Eigenvalue = 6.440, Variance = 49.54%*		
2.3.1. I am willing to turn off or unplug electronic devises when not need		.978	.838
2.3.2. I intend to turn off or unplug electronic devises when not need		.879	.878
2.3.3. I plan to turn off or unplug electronic devises when not need		.895	.900
2.3.4. I will turn off or unplug electronic devises when not need		.897	.875
Factor 2: Attitudes.	*Cronbach's α = .79, Eigenvalue = 1.763, Variance = 13.56%*		
3.3.1. I believe that turning off or unplugging electronic devises when not need will help reduce pollution		.781	.704
3.3.3. I believe that turning off or unplugging electronic devises when not need will help conserve natural resources		.783	.700
3.3.4. I feel good about myself when I turn off or unplug electronic devises when not need		.803	.730
Factor 3: Perceived Behavioural Control.	*Cronbach's α = .82, Eigenvalue = 1.322, Variance = 10.17%*		
5.3.1. I know what electronic items can be turned off or unplugged when not in need		.826	.707
5.3.2. I know where I can turn off or unplug all of my electronic devices when not in need		.917	.826
5.3.3. I know how to turn off or unplug all of my electronic devices when not in need		.805	.747
Factor 4: Moral Norms.	*Cronbach's α = .86, Eigenvalue = .710, Variance = 5.46%*		
6.3.2. I would feel guilty if I did **not** turn off or unplug electronic devices when not in need		.807	.805
6.3.3. **Not** turning off or unplugging electronic devices when not in need go against my principles		.905	.827
6.3.4. Everybody should share the responsibility to turn off or unplug electronic devices when not in need		.740	.697
Total variance = 78.73%			
KMO = .886			
χ^2 = 1651.997			
df = 78			
Sig. < .001			

S4 Table D. The results of exploratory factor analysis for TPB extension with moral norms (Models 2, 4).

Factors and items	Factor loadings	Communalities
**Behaviour 4 – Air Conditioning**		
Factor 1: Behaviour-Intention. *Cronbach's α = .93, Eigenvalue = 6.824, Variance = 45.50%*		
1.4. Reduce air conditioning	.751	.540
2.4.1. I am willing to reduce air conditioning when not need	.900	.857
2.4.2. I intend to reduce air conditioning when not need	.935	865
2.4.3. I plan to reduce air conditioning when not need	.897	.875
2.4.4. I will reduce air conditioning when not need	.907	862
Factor 2: Attitudes. *Cronbach's α = .80, Eigenvalue = .887, Variance = 5.92%*		
3.4.1. I believe that reducing air conditioning when not in need will help reduce pollution	.907	.807
3.4.3. I believe that reducing air conditioning when not in need will help conserve natural resources	.880	.800
3.4.4. I feel good about myself when I reduce air conditioning that's not in need	.473	.584
Factor 3: Perceived Behavioural Control. *Cronbach's α = .83, Eigenvalue = 1.754, Variance = 11.96%*		
5.4.1. I know what air conditioning systems can be reduced when not in need	.776	.672
5.4.2. I know where I can reduce air conditioning when not in need	.896	.817
5.4.3. I know how to reduce air conditioning when not in need	.914	.787
Factor 4: Moral Norms. *Cronbach's α = .90, Eigenvalue = .2.120, Variance = 14.14%*		
6.4.1. It would be wrong of me **not** to reduce air conditioning when not in need	.923	.780
6.4.2. I would feel guilty if I did **not** recycle my recyclables reduce air conditioning when not in need	.905	.830
6.4.3. **Not** reducing air conditioning when not in need goes against my principles	.816	.759
6.4.4. Everybody should share the responsibility to reduce air conditioning when not in need	.808	.751
Total variance = 77.24%		
KMO = .883		
χ^2 = 2003.279		
df = 105		
Sig. = .000		

S4 Table E. The results of exploratory factor analysis for TPB extension with moral norms (Models 2, 4).

Factors and items	Factor loadings	Communalities
**Behaviour 5 – Transport Use**		
Factor 1: Behaviour-Intention. Cronbach's α = .93, Eigenvalue = 6.669, Variance = 47.64%		
1.5. Reduce driving, and walk, bike or use public transportation	.543	.461
2.5.1. I am willing to reduce driving, and instead walk, bike or use public transportation	.913	.830
2.5.2. I intend to reduce driving, and instead walk, bike or use public transportation	.935	.885
2.5.3. I plan to reduce driving, and instead walk, bike or use public	.937	.863
2.5.4. I will reduce driving, and instead walk, bike or use public transportation	.953	.889
Factor 2: Attitudes. Cronbach's α = .76, Eigenvalue = .847, Variance = 6.05%		
3.1.1. I believe that my recycling behavior will help reduce pollution	.946	.816
3.1.3. I believe that my recycling behavior will help conserve natural resources	.814	.700
3.1.4. I feel good about myself when I recycle	.510	.615
Factor 3: Perceived Behavioural Control. Cronbach's α = .80, Eigenvalue = 1.426, Variance = 10.19%		
5.5.1. I know what route I can take in an attempt to reduce driving and instead walk, bike, or take public transportation	.832	.774
5.5.2. I know where I can reasonably travel to if I choose to reduce driving and instead walk, bike, or take public transportation	.882	.761
5.5.3. I know how to reduce driving and instead walk, bike, or take public transportation	.824	.680
Factor 4: Moral Norms. Cronbach's α = .90, Eigenvalue = 1.831, Variance = 13.08%		
6.5.1. It would be wrong of me **not** to reduce driving, and instead walk, bike or use public transportation	.955	.834
6.5.2. I would feel guilty if I did **not** reduce driving, and instead walk, bike or use public transportation	.886	.855
6.5.3. **Not** reducing driving, and instead walk, bike or use public transportation goes against my principles	.808	.809

Total variance = 76.95%

KMO = .874

χ^2 = 1792.180

df = 91

Sig. = .000

S4 Table F. The results of exploratory factor analysis for TPB extension with moral norms (Models 2, 4).

Factors and items	Factor loadings	Communalities
**Behaviour 7 – Local Products**		
Factor 1: Behaviour-Intention. *Cronbach's α = .92, Eigenvalue = 7.342, Variance = 48.95%*		
2.7.1. I am willing to buy local products or locally produced foods	.894	.764
2.7.2. I intend to buy local products or locally produced foods	.853	.844
2.7.3. I plan to buy local products or locally produced foods	.893	.812
2.7.4. I will buy local products or locally produced foods	.916	.844
Factor 2: Attitudes. *Cronbach's α = .83, Eigenvalue = 1.259, Variance = 8.39%*		
3.7.1. I believe that buying local products or locally produced foods will help reduce pollution	.803	.761
3.7.2. I believe that buying local products or locally produced foods will help reduce wasteful use of landfills	.761	.642
3.7.3. I believe that buying local products or locally produced foods will help conserve natural resources	.927	.800
3.7.4. I feel good about myself when I buy local products or locally produced foods	.532	.575
Factor 3: Perceived Behavioural Control. *Cronbach's α = .85, Eigenvalue = .982, Variance = 6.55%*		
5.7.1. I know what I can do to be able to buy local products or locally produced foods	.843	.798
5.7.2. I know where I can buy local products or locally produced foods	.940	.839
5.7.3. I know how to buy local products or locally produced foods	.827	.737
Factor 4: Moral Norms. *Cronbach's α = .87, Eigenvalue = 1.854, Variance = 12.36%*		
6.7.1. It would be wrong of me **not** to buy local products or locally produced foods	.915	.774
6.7.2. I would feel guilty if I did **not** buy local products or locally produced foods	.751	.764
6.7.3. **Not** buying local products or locally produced foods goes against my principles	.861	.776
6.7.4. Everybody should share the responsibility to buy local products or locally produced foods	.751	.708
Total variance = 76.25%		
KMO = .892		
χ^2 = 1861.088		
df = 105		
Sig. = .000		

S4 Table G. The results of exploratory factor analysis for TPB extension with moral norms (Models 2, 4).

Factors and items	Factor loadings	Communalities
**Behaviour 9 – Plastic Bags**		
Factor 1: Behaviour-Intention. Cronbach's α = .92, Eigenvalue = 6.603, Variance = 44.02%		
1.9. Reduce using plastic bags, or use own bag when shopping	.787	.552
2.9.1. I am willing to reduce using plastic bags, or use own bag when shopping	.869	.774
2.9.2. I intend to reduce using plastic bags, or use own bag when shopping	.876	.848
2.9.3. I plan to reduce using plastic bags, or use own bag when shopping	.872	.855
2.9.4. I will reduce using plastic bags, or use own bag when shopping	.932	.842
Factor 2: Attitudes. Cronbach's α = .76, Eigenvalue = 2.090, Variance = 13.94%		
3.9.1. I believe that reducing the use of plastic bags, or using own bag when shopping will help reduce pollution	.803	.702
3.9.2. I believe that reducing the use plastic bags, or using own bag when shopping will help reduce wasteful use of landfills	.761	.725
3.9.3. I believe that reducing the use of plastic bags, or using own bag when shopping will help conserve natural resources	.927	.733
Factor 3: Perceived Behavioural Control. Cronbach's α = .82, Eigenvalue = 1.658, Variance = 11.05%		
5.9.1. I know what I can do to reduce using plastic bags, or use own bag when shopping	.843	.796
5.9.2. I know where I can reduce using plastic bags, or use own bag when shopping	.940	.749
5.9.3. I know how to reduce using plastic bags, or use own bag when shopping	.827	.690
Factor 4: Moral Norms. Cronbach's α = .89, Eigenvalue = 2.091, Variance = 13.94%		
6.9.1 It would be wrong of me **not** to reduce using plastic bags, or **not** to use own bag when shopping	.915	.719
6.9.2. I would feel guilty if I did **not** reduce using plastic bags, or use own bag when shopping	.751	.850
6.9.3. **Not** reducing the use of plastic bags, or **not** using own bag when shopping goes against my principles	.861	.774
6.9.4. Everybody should share the responsibility to reduce using plastic bags, or use own bag when shopping	.751	.805
Total variance = 76.10%		
KMO = .864		
χ^2 = 1841.492		
df = 105		
Sig. = .000		
