# Supplementary material for: The Theory of Planned Behaviour doesn’t reveal ’attitude-behaviour’ gap? Contrasting the effects of moral norms vs. idealism and relativism in predicting pro-environmental behaviours
Source: PLoS One. 2023 Nov 27;18(11):e0290818. doi: 10.1371/journal.pone.0290818 (PMC10681191; doi:10.1371/journal.pone.0290818)
Supplement: S5 Table — (PDF) [file pone.0290818.s015.pdf]

S5 Table A. The results of exploratory factor analysis for TPB extension with EPQ (Models 3, 5).

Factors and items	Factor loadings	Communalities
**Behaviour 1 – Recycling**		
Factor 1: Behaviour-Intention. Cronbach's α = .88, Eigenvalue = 3.410, Variance = 11.00%		
1.1. Recycle newspapers, plastics, cans and glass	.783	.529
2.1.1. I am willing to recycle newspapers, plastics, cans and glass	.591	.677
2.1.2. I intend to recycle newspapers, plastics, cans and glass	.878	.843
2.1.3. I plan to recycle newspapers, plastics, cans and glass	.831	.737
2.1.4. I will recycle newspapers, plastics, cans and glass	.786	.763
Factor 2: Attitudes. Cronbach's α = .86, Eigenvalue = 1.557, Variance = 5.02%		
3.1.1. I believe that my recycling behavior will help reduce pollution	.841	.767
3.1.2. I believe that my recycling behavior will help reduce wasteful use of landfills	.767	.689
3.1.3. I believe that my recycling behavior will help conserve natural resources	.827	.676
3.1.4. I feel good about myself when I recycle	.742	.684
Factor 3: Perceived Behavioural Control. Cronbach's α = .81, Eigenvalue = 1.315, Variance = 4.24%		
5.1.1. I know what items can be recycled	.695	.660
5.1.2. I know where I can recycle newspapers, plastics, cans and glass	.824	.753
5.1.3. I know how to recycle my recyclables	.782	.758
Factor 4: EPQ: Relativism. Cronbach's α = .92, Eigenvalue = 7.961, Variance = 25.68%		
7.1.1.There are no ethical principles that are so important that they should be a part of any code of ethics	.565	.523
7.1.2.What is ethical varies from one situation and society to another	.689	.502
7.1.3.Moral standards should be seen as being individualistic; what one person considers to be moral may be judged to be immoral by another person	.875	.734
7.1.4.Different types of moralities cannot be compared as to "rightness"	.828	.692
7.1.5.Questions of what is ethical for everyone can never be resolved since what is moral or immoral is up to the individual	.892	.782
7.1.6.Moral standards are simply personal rules that indicate how a person should behave, which should not be applied in making judgments of others	.798	.716
7.1.7.Ethical considerations in interpersonal relations are so complex that individuals should be allowed to formulate their own individual codes	.818	.717
7.1.8.Rigidly codifying an ethical position that prevents certain types of actions could stand in the way of better human relations and adjustment	.747	.560
7.1.9.No rule concerning lying can be formulated; whether a lie is permissible or not permissible totally depends upon the situation	.746	.585
7.1.10.Whether a lie is judged to be moral or immoral depends upon the circumstances surrounding the action	.602	.522

Factor 5: EPQ: Idealism.	*Cronbach's α = .90, Eigenvalue = 6.064, Variance = 19.56%*	
7.2.1. A person should make certain that their actions never intentionally harm another even to a small degree	.779	.687
7.2.2. Risks to another should never be tolerated, irrespective of how small the risks might be	.820	.646
7.2.3. The existence of potential harm to others is always wrong, irrespective of the benefits to be gained	.865	.769
7.2.4. One should never psychologically or physically harm another person	.745	.593
7.2.5. One should not perform an action which might in any way threaten the dignity and welfare of another individual	.759	.665
7.2.6. If an action could harm an innocent other, then it should not be done	.747	.630
7.2.8. The dignity and welfare of the people should be the most important concern in any society	.595	.506
7.2.9. It is never necessary to sacrifice the welfare of others	.768	.580
7.2.10 Moral actions are those which closely match ideals of the most "perfect" action	.587	.359
Total variance = 65.51%		
KMO = .865		
χ^2 = 3747.310		
df = 465		
Sig. = .000		

S5 Table B. The results of exploratory factor analysis for TPB extension with EPQ (Models 3, 5).

Factors and items		Factor loadings	Communalities
**Behaviour 2 – Composting**			
Factor 1: Behaviour-Intention.	*Cronbach's α = .94, Eigenvalue = 3.495, Variance = 12.48%*		
1.2. Compost kitchen waste		.958	.699
2.2.1. I am willing to compost kitchen waste		.652	.771
2.2.2. I intend to compost kitchen waste		.860	.879
2.2.3. I plan to compost kitchen waste		.869	.878
2.2.4. I will compost kitchen waste		.849	.861
Factor 2: Attitudes.	*Cronbach's α = .85, Eigenvalue = 1.693, Variance = 6.05%*		
3.2.1. I believe that my composting kitchen waste items will help reduce pollution		.806	.725
3.2.2. I believe that my composting kitchen waste items will help reduce wasteful use of landfills		.940	.778
3.2.3. I believe that my composting kitchen waste items will help conserve natural resources		.888	.738
3.2.4. I feel good about myself when I compost kitchen waste items		.559	.612
Factor 3: Perceived Behavioural Control.	*Cronbach's α = .87, Eigenvalue = 1.061, Variance = 3.789%*		
5.2.1. I know what kitchen waste items can be composted		.889	.822
5.2.2. I know I can compost kitchen waste		.803	.863
5.2.3. I know how to compost my compostable items		.812	.876
Factor 4: EPQ: Relativism.	*Cronbach's α = .92, Eigenvalue = 8.319, Variance = 29.71%*		
7.1.2.What is ethical varies from one situation and society to another		.735	.550
7.1.3.Moral standards should be seen as being individualistic; what one person considers to be moral may be judged to be immoral by another person		.833	.733
7.1.4.Different types of moralities cannot be compared as to "rightness"		.820	.690
7.1.5.Questions of what is ethical for everyone can never be resolved since what is moral or immoral is up to the individual		.876	.780
7.1.6.Moral standards are simply personal rules that indicate how a person should behave, which should not be applied in making judgments of others		.815	.701
7.1.7.Ethical considerations in interpersonal relations are so complex that individuals should be allowed to formulate their own individual codes		.827	.704
7.1.8.Rigidly codifying an ethical position that prevents certain types of actions could stand in the way of better human relations and adjustment		.670	.507
7.1.9.No rule concerning lying can be formulated; whether a lie is permissible or not permissible totally depends upon the situation		.750	.587
7.1.10.Whether a lie is judged to be moral or immoral depends upon the circumstances surrounding the action		.689	.490

Factor 5: EPQ: Idealism. *Cronbach's α = .90, Eigenvalue = 5.265, Variance = 18.80%*		
7.2.1. A person should make certain that their actions never intentionally harm another even to a small degree	.801	.654
7.2.2. Risks to another should never be tolerated, irrespective of how small the risks might be	.786	.634
7.2.3. The existence of potential harm to others is always wrong, irrespective of the benefits to be gained	.788	.725
7.2.4. One should never psychologically or physically harm another person	.812	.665
7.2.5. One should not perform an action which might in any way threaten the dignity and welfare of another individual	.835	.694
7.2.6. If an action could harm an innocent other, then it should not be done	.824	.679
7.2.8. The dignity and welfare of the people should be the most important concern in any society	.745	.536
Total variance = 70.83%		
KMO = .874		
χ^2 = 3904.223		
df = 378		
Sig. = .000		

S5 Table C. The results of exploratory factor analysis for TPB extension with EPQ (Models 3, 5).

Factors and items	Factor loadings	Communalities
**Behaviour 3 – Electronic Devices**		
Factor 1: Behaviour-Intention. Cronbach's α = .91, Eigenvalue = 2.972, Variance = 10.25%		
1.3. Turn off or unplug electronic devises when not need	.659	.401
2.3.1. I am willing to turn off or unplug electronic devises when not need	.869	.784
2.3.2. I intend to turn off or unplug electronic devises when not need	.911	.868
2.3.3. I plan to turn off or unplug electronic devises when not need	.900	.867
2.3.4. I will turn off or unplug electronic devises when not need	.848	.831
Factor 2: Attitudes. Cronbach's α = .70, Eigenvalue = 1.318, Variance = 4.54%		
3.3.1. I believe that turning off or unplugging electronic devises when not need will help reduce pollution	.712	.655
3.3.2. I believe that turning off or unplugging electronic devises when not need will help reduce wasteful use of landfills	.559	.612
3.3.3. I believe that turning off or unplugging electronic devises when not need will help conserve natural resources	.842	.713
3.3.4. I feel good about myself when I turn off or unplug electronic devises when not need	.578	.590
Factor 3: Perceived Behavioural Control. Cronbach's α = .82, Eigenvalue = 2.132, Variance = 7.35%		
5.3.1. I know what electronic items can be turned off or unplugged when not in need	.681	.567
5.3.2. I know where I can turn off or unplug all of my electronic devices when not in need	.830	.758
5.3.3. I know how to turn off or unplug all of my electronic devices when not in need	.839	.730
Factor 4: EPQ: Relativism. Cronbach's α = .92, Eigenvalue = 7.318, Variance = 25.23%		
7.1.2.What is ethical varies from one situation and society to another	.704	.544
7.1.3.Moral standards should be seen as being individualistic; what one person considers to be moral may be judged to be immoral by another person	.834	.729
7.1.4.Different types of moralities cannot be compared as to "rightness"	.824	.698
7.1.5.Questions of what is ethical for everyone can never be resolved since what is moral or immoral is up to the individual	.851	.783
7.1.6.Moral standards are simply personal rules that indicate how a person should behave, which should not be applied in making judgments of others	.809	.705
7.1.7.Ethical considerations in interpersonal relations are so complex that individuals should be allowed to formulate their own individual codes	.826	.711
7.1.8.Rigidly codifying an ethical position that prevents certain types of actions could stand in the way of better human relations and adjustment	.639	.523
7.1.9.No rule concerning lying can be formulated; whether a lie is permissible or not permissible totally depends upon the situation	.808	.646
7.1.10.Whether a lie is judged to be moral or immoral depends upon the circumstances surrounding the action	.764	.592

Factor 5: EPQ: Idealism. *Cronbach's α = .89, Eigenvalue = 5.754, Variance = 19.84%*		
7.2.1. A person should make certain that their actions never intentionally harm another even to a small degree	.762	.651
7.2.2. Risks to another should never be tolerated, irrespective of how small the risks might be	.815	.648
7.2.3. The existence of potential harm to others is always wrong, irrespective of the benefits to be gained	.802	.752
7.2.4. One should never psychologically or physically harm another person	.740	.643
7.2.5. One should not perform an action which might in any way threaten the dignity and welfare of another individual	.830	.684
7.2.6. If an action could harm an innocent other, then it should not be done	.826	.678
7.2.8. The dignity and welfare of the people should be the most important concern in any society	.661	.523
7.2.9. It is never necessary to sacrifice the welfare of others	.768	.600
Total variance = 67.22%		
KMO = .859		
$\chi^2 = 3529.823$		
df = 406		
Sig. = .000		

S5 Table D. The results of exploratory factor analysis for TPB extension with EPQ (Models 3, 5).

Factors and items	Factor loadings	Communalities
**Behaviour 4 – Air Conditioning**		
Factor 1: Behaviour-Intention. *Cronbach's α = .93, Eigenvalue = 3.615, Variance = 11.66%*		
1.4. Reduce air conditioning	.655	.471
2.4.1. I am willing to reduce air conditioning when not need	.913	.814
2.4.2. I intend to reduce air conditioning when not need	.936	.882
2.4.3. I plan to reduce air conditioning when not need	.966	.853
2.4.4. I will reduce air conditioning when not need	.929	.874
Factor 2: Attitudes. *Cronbach's α = .80, Eigenvalue = 1.434, Variance = 4.63%*		
3.4.1. I believe that reducing air conditioning when not in need will help reduce pollution	.878	.751
3.4.3. I believe that reducing air conditioning when not in need will help conserve natural resources	.859	.696
3.4.4. I feel good about myself when I reduce air conditioning that's not in need	.863	.595
Factor 3: Perceived Behavioural Control. *Cronbach's α = .83, Eigenvalue = 2.250, Variance = 7.26%*		
5.4.1. I know what air conditioning systems can be reduced when not in need	.736	.768
5.4.2. I know where I can reduce air conditioning when not in need	.859	.749
5.4.3. I know how to reduce air conditioning when not in need	.864	.696
Factor 4: EPQ: Relativism. *Cronbach's α = .92, Eigenvalue = 6.942, Variance = 22.39%*		
7.1.2.What is ethical varies from one situation and society to another	.718	.501
7.1.3.Moral standards should be seen as being individualistic; what one person considers to be moral may be judged to be immoral by another person	.848	.735
7.1.4.Different types of moralities cannot be compared as to "rightness"	.819	.702
7.1.5.Questions of what is ethical for everyone can never be resolved since what is moral or immoral is up to the individual	.879	.777
7.1.6.Moral standards are simply personal rules that indicate how a person should behave, which should not be applied in making judgments of others	.822	.715
7.1.7.Ethical considerations in interpersonal relations are so complex that individuals should be allowed to formulate their own individual codes	.833	.713
7.1.8.Rigidly codifying an ethical position that prevents certain types of actions could stand in the way of better human relations and adjustment	.686	.521
7.1.9.No rule concerning lying can be formulated; whether a lie is permissible or not permissible totally depends upon the situation	.773	.585
7.1.10.Whether a lie is judged to be moral or immoral depends upon the circumstances surrounding the action	.696	.446
Factor 5: EPQ: Idealism. *Cronbach's α = .90, Eigenvalue = 6.038, Variance = 19.48%*		

7.2.1. A person should make certain that their actions never intentionally harm another even to a small degree	.780	.656
7.2.2. Risks to another should never be tolerated, irrespective of how small the risks might be	.831	.628
7.2.3. The existence of potential harm to others is always wrong, irrespective of the benefits to be gained	.839	.736
7.2.4. One should never psychologically or physically harm another person	.712	.716
7.2.5. One should not perform an action which might in any way threaten the dignity and welfare of another individual	.763	.613
7.2.6. If an action could harm an innocent other, then it should not be done	.776	.671
7.2.8. The dignity and welfare of the people should be the most important concern in any society	.668	.682
7.2.9. It is never necessary to sacrifice the welfare of others	.775	.506
7.2.10 Moral actions are those which closely match ideals of the most "perfect" action	.519	.588

Total variance = 65.42%

KMO = .846

χ^2 = 3390.106

df = 378

Sig. = .000

S5 Table E. The results of exploratory factor analysis for TPB extension with EPQ (Models 3, 5).

Factors and items	Factor loadings	Communalities
**Behaviour 5 – Transport Use**		
Factor 1: Behaviour-Intention. Cronbach's α = .93, Eigenvalue = 3.369, Variance = 12.03%		
1.5. Reduce driving, and walk, bike or use public transportation	.656	.471
2.5.1. I am willing to reduce driving, and instead walk, bike or use public transportation	.872	.814
2.5.2. I intend to reduce driving, and instead walk, bike or use public transportation	.953	.882
2.5.3. I plan to reduce driving, and instead walk, bike or use public	.935	.853
2.5.4. I will reduce driving, and instead walk, bike or use public transportation	.926	.874
Factor 2: Attitudes. Cronbach's α = .76, Eigenvalue = 1.358, Variance = 4.85%		
3.5.1. I believe that reducing driving, and instead walking, biking or using public transportation will help reduce pollution	.861	.751
3.5.3. I believe that reducing driving, and instead walking, biking or using public transportation will help conserve natural resources	.837	.696
3.5.4. I feel good about myself when I reduce driving, and instead walk, bike or use public transportation	.603	.595
Factor 3: Perceived Behavioural Control. Cronbach's α = .80, Eigenvalue = 1.792, Variance = 6.40%		
5.5.1. I know what route I can take in an attempt to reduce driving and instead walk, bike, or take public transportation	.819	.768
5.5.2. I know where I can reasonably travel to if I choose to reduce driving and instead walk, bike, or take public transportation	.860	.749
5.5.3. I know how to reduce driving and instead walk, bike, or take public transportation	.822	.696
Factor 4: EPQ: Relativism. Cronbach's α = .92, Eigenvalue = 6.880, Variance = 24.57%		
7.1.2.What is ethical varies from one situation and society to another	.706	.501
7.1.3.Moral standards should be seen as being individualistic; what one person considers to be moral may be judged to be immoral by another person	.860	.735
7.1.4.Different types of moralities cannot be compared as to "rightness"	.817	.702
7.1.5.Questions of what is ethical for everyone can never be resolved since what is moral or immoral is up to the individual	.877	.777
7.1.6.Moral standards are simply personal rules that indicate how a person should behave, which should not be applied in making judgments of others	.830	.715
7.1.7.Ethical considerations in interpersonal relations are so complex that individuals should be allowed to formulate their own individual codes	.835	.713
7.1.8.Rigidly codifying an ethical position that prevents certain types of actions could stand in the way of better human relations and adjustment	.688	.521
7.1.9.No rule concerning lying can be formulated; whether a lie is permissible or not permissible totally depends upon the situation	.754	.585
7.1.10.Whether a lie is judged to be moral or immoral depends upon the circumstances surrounding the action	.670	.446

Factor 5: EPQ: Idealism.	*Cronbach's α = .89, Eigenvalue = 5.504, Variance = 19.66%*	
7.2.1. A person should make certain that their actions never intentionally harm another even to a small degree	.777	.656
7.2.2. Risks to another should never be tolerated, irrespective of how small the risks might be	.791	.628
7.2.3. The existence of potential harm to others is always wrong, irrespective of the benefits to be gained	.816	.716
7.2.4. One should never psychologically or physically harm another person	.735	.613
7.2.5. One should not perform an action which might in any way threaten the dignity and welfare of another individual	.815	.671
7.2.6. If an action could harm an innocent other, then it should not be done	.826	.682
7.2.8. The dignity and welfare of the people should be the most important concern in any society	.650	.506
7.2.9. It is never necessary to sacrifice the welfare of others	.761	.588

Total variance = 67.51%

KMO = .846

χ^2 = 3390.106

df = 378

Sig. = .000

S5 Table F. The results of exploratory factor analysis for TPB extension with EPQ (Models 3, 5).

Factors and items	Factor loadings	Communalities
**Behaviour 7 – Local Products**		
Factor 1: Behaviour-Intention. Cronbach's α = .88, Eigenvalue = 3.287, Variance = 10.96%		
1.7. Buy local products or locally produced foods	.785	.357
2.7.1. I am willing to buy local products or locally produced foods	.853	.698
2.7.2. I intend to buy local products or locally produced foods	.874	.848
2.7.3. I plan to buy local products or locally produced foods	.879	.809
2.7.4. I will buy local products or locally produced foods	.899	.777
Factor 2: Attitudes. Cronbach's α = .83, Eigenvalue = 1.772, Variance = 5.91%		
3.7.1. I believe that buying local products or locally produced foods will help reduce pollution	.726	.739
3.7.2. I believe that buying local products or locally produced foods will help reduce wasteful use of landfills	.670	.617
3.7.3. I believe that buying local products or locally produced foods will help conserve natural resources	.871	.814
3.7.4. I feel good about myself when I buy local products or locally produced foods	.648	.602
Factor 3: Perceived Behavioural Control. Cronbach's α = .85, Eigenvalue = 1.264, Variance = 4.21%		
5.7.1. I know what I can do to be able to buy local products or locally produced foods	.820	.694
5.7.2. I know where I can buy local products or locally produced foods	.777	.714
5.7.3. I know how to buy local products or locally produced foods	.825	.704
Factor 4: EPQ: Relativism. Cronbach's α = .92, Eigenvalue = 8.028, Variance = 26.76%		
7.1.1.There are no ethical principles that are so important that they should be a part of any code of ethics	.684	.544
7.1.2.What is ethical varies from one situation and society to another	.700	.553
7.1.3.Moral standards should be seen as being individualistic; what one person considers to be moral may be judged to be immoral by another person	.822	.722
7.1.4.Different types of moralities cannot be compared as to "rightness"	.840	.708
7.1.5.Questions of what is ethical for everyone can never be resolved since what is moral or immoral is up to the individual	.878	.773
7.1.6.Moral standards are simply personal rules that indicate how a person should behave, which should not be applied in making judgments of others	.804	.696
7.1.7.Ethical considerations in interpersonal relations are so complex that individuals should be allowed to formulate their own individual codes	.793	.693
7.1.9.No rule concerning lying can be formulated; whether a lie is permissible or not permissible totally depends upon the situation	.752	.585
7.1.10.Whether a lie is judged to be moral or immoral depends upon the circumstances surrounding the action	.650	.429

Factor 5: EPQ: Idealism. *Cronbach's α = .89, Eigenvalue = 5.507, Variance = 18.36%*		
7.2.1. A person should make certain that their actions never intentionally harm another even to a small degree	.764	.663
7.2.2. Risks to another should never be tolerated, irrespective of how small the risks might be	.766	.639
7.2.3. The existence of potential harm to others is always wrong, irrespective of the benefits to be gained	.792	.736
7.2.4. One should never psychologically or physically harm another person	.773	.648
7.2.5. One should not perform an action which might in any way threaten the dignity and welfare of another individual	.840	.702
7.2.6. If an action could harm an innocent other, then it should not be done	.848	.686
7.2.8. The dignity and welfare of the people should be the most important concern in any society	.694	.543
7.2.9. It is never necessary to sacrifice the welfare of others	.710	.671
Total variance = 66.07%		
KMO = .856		
χ^2 = 3667.026		
df = 435		
Sig. = .000		

S5 Table G. The results of exploratory factor analysis for TPB extension with EPQ (Models 3, 5).

Factors and items		Factor loadings	Communalities
**Behaviour 9 – Plastic Bags**			
Factor 1: Behaviour-Intention.	*Cronbach's α = .92, Eigenvalue = 3.510, Variance = 11.32%*		
1.9. Reduce using plastic bags, or use own bag when shopping		.684	.576
2.9.1. I am willing to reduce using plastic bags, or use own bag when shopping		.865	.776
2.9.2. I intend to reduce using plastic bags, or use own bag when shopping		.886	.841
2.9.3. I plan to reduce using plastic bags, or use own bag when shopping		.893	.858
2.9.4. I will reduce using plastic bags, or use own bag when shopping		.920	.850
Factor 2: Attitudes.	*Cronbach's α = .81, Eigenvalue = 1.414, Variance = 4.56%*		
3.9.1. I believe that reducing the use of plastic bags, or using own bag when shopping will help reduce pollution		.775	.766
3.9.2. I believe that reducing the use plastic bags, or using own bag when shopping will help reduce wasteful use of landfills		.725	.628
3.9.3. I believe that reducing the use of plastic bags, or using own bag when shopping will help conserve natural resources		.822	.714
3.9.4. I feel good about myself when I reduce the use of plastic bags, or use own bag when shopping		.666	.635
Factor 3: Perceived Behavioural Control.	*Cronbach's α = .82, Eigenvalue = 2.124, Variance = 6.85%*		
5.9.1. I know what I can do to reduce using plastic bags, or use own bag when shopping		.852	.799
5.9.2. I know where I can reduce using plastic bags, or use own bag when shopping		.819	.844
5.9.3. I know how to reduce using plastic bags, or use own bag when shopping		.800	.790
Factor 4: EPQ: Relativism.	*Cronbach's α = .92, Eigenvalue = 7.461, Variance = 24.07%*		
7.1.1.There are no ethical principles that are so important that they should be a part of any code of ethics		.555	.512
7.1.2.What is ethical varies from one situation and society to another		.745	
7.1.3.Moral standards should be seen as being individualistic; what one person considers to be moral may be judged to be immoral by another person		.834	.746
7.1.4.Different types of moralities cannot be compared as to "rightness"		.821	.703
7.1.5.Questions of what is ethical for everyone can never be resolved since what is moral or immoral is up to the individual		.859	.780
7.1.6.Moral standards are simply personal rules that indicate how a person should behave, which should not be applied in making judgments of others		.837	.726
7.1.7.Ethical considerations in interpersonal relations are so complex that individuals should be allowed to formulate their own individual codes		.831	.719
7.1.8.Rigidly codifying an ethical position that prevents certain types of actions could stand in the way of better human relations and adjustment		.647	.532

7.1.9.No rule concerning lying can be formulated; whether a lie is permissible or not permissible totally depends upon the situation	.754	.612
7.1.10.Whether a lie is judged to be moral or immoral depends upon the circumstances surrounding the action	.726	.537
Factor 5: EPQ: Idealism. *Cronbach's α = .90, Eigenvalue = 5.972, Variance = 19.27%*		
7.2.1. A person should make certain that their actions never intentionally harm another even to a small degree	.776	.658
7.2.2. Risks to another should never be tolerated, irrespective of how small the risks might be	.814	.664
7.2.3. The existence of potential harm to others is always wrong, irrespective of the benefits to be gained	.810	.736
7.2.4. One should never psychologically or physically harm another person	.727	.626
7.2.5. One should not perform an action which might in any way threaten the dignity and welfare of another individual	.799	.688
7.2.6. If an action could harm an innocent other, then it should not be done	.785	.684
7.2.8. The dignity and welfare of the people should be the most important concern in any society	.656	.511
7.2.9. It is never necessary to sacrifice the welfare of others	.753	.548
7.2.10 Moral actions are those which closely match ideals of the most "perfect" action	.549	.373
Total variance = 66.09%		
KMO = .850		
χ^2 = 3789.313		
df = 465		
Sig. = .000		
