# Supplementary material for: The Theory of Planned Behaviour doesn’t reveal ’attitude-behaviour’ gap? Contrasting the effects of moral norms vs. idealism and relativism in predicting pro-environmental behaviours
Source: PLoS One. 2023 Nov 27;18(11):e0290818. doi: 10.1371/journal.pone.0290818 (PMC10681191; doi:10.1371/journal.pone.0290818)
Supplement: S1 Text — (PDF) [file pone.0290818.s016.pdf]

S1 Text. Experimental Protocol

Predicting Pro-Environmental Behaviour: the Effectiveness of Moral Norms vs. EPQ in Assessing 'Attitude – Behaviour' Gap

Aims of the experiment:

(i) To find out whether 'attitude – behaviour' gap occurs between attitudes and intentions or between intentions and behaviours

(ii) To find out the effects of moral component in predicting 'attitude-behaviour' gap

	Construct	Items	Reference
**Part 1**			
1.	Behaviour	10 items	Huang, (2016)
**Part 2**			
2.	Intention	4 items per behaviour x 10	Yazdanpanah & Forouzani (2015)
a.	Attention check 1		
**Part 3**			
3.	Attitudes	4 items per behaviour x 10	Ramayah, Lee, & Lim, (2012)
b.	Attention check 2		
**Part 4**			
4.	Subjective Norm	4 items per behaviour x 10	Wan, Cheung, & Shen, (2012)
c.	Attention check 3		
**Part 5**			
5.	Perceived Behaviour Control	4 items per behaviour x 10	Wan, Cheung, & Shen, (2012)
d.	Attention check 4		
**Part 6**			
6.	Moral Norms	4 items per behaviour x 10	Wan, Cheung, & Shen, (2012)
7.	Ethics Positions	10 x idealism, 10 x relativism	Forsyth, D. R. (1980)
e.	Attention check 5		

8.	Demographics	8 questions	

Note: Part 1 was launched 1 month away from other parts. After 1 month, each of the following parts became available after completion of the part before.

**Study: Predicting Pro-Environmental Behaviour: the Effectiveness of Moral Norms vs. EPQ in Assessing 'Attitude – Behaviour' Gap**	
**1. Scale: Behaviour**	**Item**
Likert: 1 (Never), 2 (Rarely), 3 (Occasionally), 4 (Sometimes), 5 (Frequently), 6 (Usually), 7 (Every time)	Please indicate the extent to which you are performing the following behaviors in your daily life:<br><br>1.1. Recycle newspapers, plastics, cans and glass<br>1.2. Compost kitchen waste<br>1.3. Turn off or unplug electronic devises when not need<br>1.4. Reduce air conditioning<br>1.5. Reduce driving, and walk, bike or use public transportation<br>1.6. Eat less meat and more vegetables<br>1.7. Buy local products or locally produced foods<br>1.8. Buy energy efficient appliances<br>1.9. Reduce using plastic bags, or use own bag when shopping<br>1.10. Bring own utensils when eating out
Huang, H. (2016). Media use, environmental beliefs, self-efficacy, and pro-environmental behavior. Journal of Business Research, 69(6), 2206-2212	
**2. Scale: Intention**	**Item**
Likert: 1 (SD) – 7 (SA)	Please indicate the extent to which you agree with the following statements:<br><br>2.1.1. I am willing to recycle newspapers, plastics, cans and glass<br>2.1.2. I intend to recycle newspapers, plastics, cans and glass<br>2.1.3. I plan to recycle newspapers, plastics, cans and glass<br>2.1.4. I will recycle newspapers, plastics, cans and glass<br><br>2.2.1. I am willing to compost kitchen waste<br>2.2.2. I intend to compost kitchen waste<br>2.2.3. I plan to compost kitchen waste<br>2.2.4. I will compost kitchen waste<br><br>2.3.1. I am willing to turn off or unplug electronic devises when not need<br>2.3.2. I intend to turn off or unplug electronic devises when not need<br>2.3.3. I plan to turn off or unplug electronic devises when not need<br>2.3.4. I will turn off or unplug electronic devises when not need<br><br>2.4.1. I am willing to reduce air conditioning when not need<br>2.4.2. I intend to reduce air conditioning when not need<br>2.4.3. I plan to reduce air conditioning when not need<br>2.4.4. I will reduce air conditioning when not need<br><br>2.5.1. I am willing to reduce driving, and instead walk, bike or use public transportation<br>2.5.2. I intend to reduce driving, and instead walk, bike or use public transportation

	2.5.3. I plan to reduce driving, and instead walk, bike or use public<br>2.5.4. I will reduce driving, and instead walk, bike or use public transportation<br><br>2.6.1. I am willing to eat less meat and more vegetables<br>2.6.2. I intend to eat less meat and more vegetables<br>2.6.3. I plan to eat less meat and more vegetables<br>2.6.4. I will eat less meat and more vegetables<br><br>2.7.1. I am willing to buy local products or locally produced foods<br>2.7.2. I intend to buy local products or locally produced foods<br>2.7.3. I plan to buy local products or locally produced foods<br>2.7.4. I will buy local products or locally produced foods<br><br>2.8.1. I am willing to buy energy efficient appliances<br>2.8.2. I intend to buy energy efficient appliances<br>2.8.3. I plan to buy energy efficient appliances<br>2.8.4. I will buy energy efficient appliances<br><br>2.9.1. I am willing to reduce using plastic bags, or use own bag when shopping<br>2.9.2. I intend to reduce using plastic bags, or use own bag when shopping<br>2.9.3. I plan to reduce using plastic bags, or use own bag when shopping<br>2.9.4. I will reduce using plastic bags, or use own bag when shopping<br><br>2.10.1. I am willing to bring own utensils when eating out<br>2.10.2. I intend to bring own utensils when eating out<br>2.10.3. I plan to bring own utensils when eating out<br>2.10.4. I will bring own utensils when eating out
Yazdanpanah, M., & Forouzani, M. (2015). Application of the Theory of Planned Behaviour to predict Iranian students' intention to purchase organic food. Journal of Cleaner Production, 107, 342-352.	
**a. Attention check 1**	**Item**
[Open answer]	Which day of the week was February 28? Please type the answer in three letters
**3. Scale: Attitudes**	**Item**
Likert: 1 (SD) – 7 (SA)	Please indicate the extent to which you agree with the following statements:<br><br>3.1.1. I believe that my recycling behavior will help reduce pollution<br>3.1.2. I believe that my recycling behavior will help reduce wasteful use of landfills<br>3.1.3. I believe that my recycling behavior will help conserve natural resources<br>3.1.4. I feel good about myself when I recycle<br><br>3.2.1. I believe that my composting kitchen waste items will help reduce pollution<br>3.2.2. I believe that my composting kitchen waste items will help reduce wasteful use of landfills

	3.2.3. I believe that my composting kitchen waste items will help conserve natural resources<br>3.2.4. I feel good about myself when I compost kitchen waste items<br><br>3.3.1. I believe that turning off or unplugging electronic devises when not need will help reduce pollution<br>3.3.2. I believe that turning off or unplugging electronic devises when not need will help reduce wasteful use of landfills<br>3.3.3. I believe that turning off or unplugging electronic devises when not need will help conserve natural resources<br>3.3.4. I feel good about myself when I turn off or unplug electronic devises when not need<br><br>3.4.1. I believe that reducing air conditioning when not in need will help reduce pollution<br>3.4.2. I believe that reducing air conditioning when not in need will help reduce wasteful use of landfills<br>3.4.3. I believe that reducing air conditioning when not in need will help conserve natural resources<br>3.4.4. I feel good about myself when I reduce air conditioning that's not in need<br><br>3.5.1. I believe that reducing driving, and instead walking, biking or using public transportation will help reduce pollution<br>3.5.2. I believe reducing driving, and instead walking, biking or using public transportation will help reduce wasteful use of landfills<br>3.5.3. I believe that reducing driving, and instead walking, biking or using public transportation will help conserve natural resources<br>3.5.4. I feel good about myself when I reduce driving, and instead walk, bike or use public transportation<br><br>3.6.1. I believe that eating less meat and more vegetables will help reduce pollution<br>3.6.2. I believe that eating less meat and more vegetables will help reduce wasteful use of landfills<br>3.6.3. I believe that eating less meat and more vegetables will help conserve natural resources<br>3.6.4. I feel good about myself when I eat less meat and more vegetables<br><br>3.7.1. I believe that buying local products or locally produced foods will help reduce pollution<br>3.7.2. I believe that buying local products or locally produced foods will help reduce wasteful use of landfills<br>3.7.3. I believe that buying local products or locally produced foods will help conserve natural resources<br>3.7.4. I feel good about myself when I buy local products or locally produced foods<br><br>3.8.1. I believe that buying energy efficient appliances will help reduce pollution<br>3.8.2. I believe that buying energy efficient appliances will help reduce wasteful use of landfills

	3.8.3. I believe that buying energy efficient appliances will help conserve natural resources<br>3.8.4. I feel good about myself when I buy energy efficient appliances<br><br>3.9.1. I believe that reducing the use of plastic bags, or using own bag when shopping will help reduce pollution<br>3.9.2. I believe that reducing the use plastic bags, or using own bag when shopping will help reduce wasteful use of landfills<br>3.9.3. I believe that reducing the use of plastic bags, or using own bag when shopping will help conserve natural resources<br>3.9.4. I feel good about myself when I reduce the use of plastic bags, or use own bag when shopping<br><br>3.10.1. I believe that bringing own utensils when eating out will help reduce pollution<br>3.10.2. I believe bringing own utensils when eating out will help reduce wasteful use of landfills<br>3.10.3. I believe that bringing own utensils when eating out will help conserve natural resources<br>3.10.4. I feel good about myself when I bring own utensils when eating out
Ramayah, T., Lee, J. W. C., & Lim, S. (2012). Sustaining the environment through recycling: An empirical study. Journal of environmental management, 102, 141-147.	
**b. Attention check 2**	**Item**
[Open answer]	Two times two equals what? Please type the answer in letters (not numbers)
**4. Scale: Subjective Norm**	**Item**
Likert: 1 (SD) – 7 (SA)	Please indicate the extent to which you agree with the following statements:<br><br>4.1.1. My friends expect me to recycle recyclables<br>4.1.2. My classmates/colleagues expect me to recycle recyclables<br>4.1.3. Media influences me to recycle recyclables<br>4.1.4. Environmental groups influence me to recycle recyclables<br><br>4.2.1. My friends expect me to compost kitchen waste<br>4.2.2. My classmates/colleagues expect me to compost kitchen waste items<br>4.2.3. Media influences me to compost compostable items<br>4.2.4. Environmental groups influence me to compost compostable items<br><br>4.3.1. My friends expect me to turn off or unplug electronic devices when not in need<br>4.3.2. My classmates/colleagues expect me to turn off or unplug electronic devices when not in need<br>4.3.3. Media influences me to turn off or unplug electronic devices when not in need<br>4.3.4. Environmental groups influence me to turn off or unplug electronic devices when not in need<br><br>4.4.1. My friends expect me to reduce air conditioning when not in need

	4.4.2. My classmates/colleagues expect me to reduce air conditioning when not in need<br>4.4.3. Media influences me to reduce air conditioning when not in need<br>4.4.4. Environmental groups influence me to reduce air conditioning when not in need<br><br>4.5.1. My friends expect me to reduce driving, and walk, bike or use public transportation<br>4.5.2. My classmates/colleagues expect me to reduce driving, and walk, bike or use public transportation<br>4.5.3. Media influences me to reduce driving, and walk, bike or use public transportation<br>4.5.4. Environmental groups influence me to reduce driving, and walk, bike or use public transportation<br><br>4.6.1. My friends expect me to eat less meat and more vegetables<br>4.6.2. My classmates/colleagues expect me to eat less meat and more vegetables<br>4.6.3. Media influences me to eat less meat and more vegetables<br>4.6.4. Environmental groups influence me to eat less meat and more vegetables<br><br>4.7.1. My friends expect me to buy local products or locally produced foods<br>4.7.2. My classmates/colleagues expect me to buy local products or locally produced foods<br>4.7.3. Media influences me to buy local products or locally produced foods<br>4.7.4. Environmental groups influence me to buy local products or locally produced foods<br><br>4.8.1. My friends expect me to buy energy efficient appliances<br>4.8.2. My classmates/colleagues expect me to buy energy efficient appliances<br>4.8.3. Media influences me to buy energy efficient appliances<br>4.8.4. Environmental groups influence me to buy energy efficient appliances<br><br>4.9.1. My friends expect me to reduce using plastic bags, or use own bag when shopping<br>4.9.2. My classmates/colleagues expect me to reduce using plastic bags, or use own bag when shopping<br>4.9.3. Media influences me to reduce using plastic bags, or use own bag when shopping<br>4.9.4. Environmental groups influence me to reduce using plastic bags, or use own bag when shopping<br><br>4.10.1. My friends expect me to bring own utensils when eating out<br>4.10.2. My classmates/colleagues expect me to bring own utensils when eating out<br>4.10.3. Media influences me to bring own utensils when eating out<br>4.10.4. Environmental groups influence me to bring own utensils when eating out

Wan, C., Cheung, R., & Shen, G. Q. (2012). Recycling attitude and behaviour in university campus: A case study in Hong Kong. Facilities.	
**c. Attention check 3**	**Item**
[Multiple choice]	Review the given list and type the object that is typically yellow: (1) snow, (2) sun, (3) grass
**5. Perceived Behaviour Control**	**Scenario**
Likert: 1 (SD) – 7 (SA)	Please indicate the extent to which you agree with the following statements:<br><br>5.1.1. I know what items can be recycled<br>5.1.2. I know where I can recycle newspapers, plastics, cans and glass<br>5.1.3. I know how to recycle my recyclables<br>5.1.4. I know I would recycle more if I had more information on recycling<br><br>5.2.1. I know what kitchen waste items can be composted<br>5.2.2. I know I can compost kitchen waste<br>5.2.3. I know how to compost my compostable items<br>5.2.4. I know I would compost more if I had more information on composting<br><br>5.3.1. I know what electronic items can be turned off or unplugged when not in need<br>5.3.2. I know where I can turn off or unplug all of my electronic devices when not in need<br>5.3.3. I know how to turn off or unplug all of my electronic devices when not in need<br>5.3.4. I know I would turn off or unplug electronic devices when not in need more often if I had more information on the consumption of electricity<br><br>5.4.1. I know what air conditioning systems can be reduced when not in need<br>5.4.2. I know where I can reduce air conditioning when not in need<br>5.4.3. I know how to reduce air conditioning when not in need<br>5.4.4. I know I would reduce air conditioning when not in need more often if I had more information on the consumption of electricity related to this air conditioning system<br><br>5.5.1. I know what route I can take in an attempt to reduce driving and instead walk, bike, or take public transportation<br>5.5.2. I know where I can reasonably travel to if I choose to reduce driving and instead walk, bike, or take public transportation<br>5.5.3. I know how to reduce driving and instead walk, bike, or take public transportation<br>5.5.4. I know I would reduce driving and instead walk, bike, or take public transportation more often if I had more information on routes to take<br><br>5.6.1. I know what to do to eat less meat and more vegetables<br>6.6.2. I know where I can eat less meat and more vegetables<br>5.6.3. I know how to eat less meat and more vegetables<br>5.6.4. I know I would to eat less meat and more vegetables if I had more information on the consumption of meat and vegetables

	5.7.1. I know what I can do to be able to buy local products or locally produced foods<br>5.7.2. I know where I can buy local products or locally produced foods<br>5.7.3. I know how to buy local products or locally produced foods<br>5.7.4. I know I would buy local products or locally produced foods more if I had more information on local production<br><br>5.8.1. I know what I can do to be able to buy energy efficient appliances<br>5.8.2. I know where I can buy energy efficient appliances<br>5.8.3. I know how to buy energy efficient appliances<br>5.8.4. I know I would buy energy efficient appliances more often if I had more information on energy consumption<br><br>5.9.1. I know what I can do to reduce using plastic bags, or use own bag when shopping<br>5.9.2. I know where I can reduce using plastic bags, or use own bag when shopping<br>5.9.3. I know how to reduce using plastic bags, or use own bag when shopping<br>5.9.4. I know I would reduce using plastic bags, or use own bag when shopping more if I had more often if I had more information on the use of plastic bags and own bags<br><br>5.10.1. I know what own utensils I can bring with me when eating out<br>5.10.2. I know where I can bring my own utensils when eating out<br>5.10.3. I know how to bring my own utensils when eating out<br>5.10.4. I know I would bring my own utensils more often when eating out if I had more information on the use of own utensils in such cases
Wan, C., Cheung, R., & Shen, G. Q. (2012). Recycling attitude and behaviour in university campus: A case study in Hong Kong. Facilities.	
**d. Attention check 4**	**Item**
[Multiple choice]	Please choose the answer which says 'two':<br>• 55<br>• 1<br>• 89<br>• 46<br>• 2
**6. Moral Norms**	**Item**
Likert: 1 (SD) – 7 (SA)	Please indicate the extent to which you agree with the following statements:<br><br>6.1.1. It would be wrong of me **not** to recycle my recyclables<br>6.1.2. I would feel guilty if I did **not** recycle my recyclables<br>6.1.3. **Not** recycling goes against my principles<br>6.1.4. Everybody should share the responsibility to recycle recyclables<br><br>6.2.1. It would be wrong of me **not** to compost my compostable items<br>6.2.2. I would feel guilty if I did **not** compost my compostable items<br>6.2.3. **Not** composting goes against my principles<br>6.2.4. Everybody should share the responsibility to compost compostable items

	6.3.1. It would be wrong of me **not** to turn off or unplug electronic devices when not in need<br>6.3.2. I would feel guilty if I did **not** turn off or unplug electronic devices when not in need<br>6.3.3. **Not** turning off or unplugging electronic devices when not in need go against my principles<br>6.3.4. Everybody should share the responsibility to turn off or unplug electronic devices when not in need<br><br>6.4.1. It would be wrong of me **not** to reduce air conditioning when not in need<br>6.4.2. I would feel guilty if I did **not** recycle my recyclables reduce air conditioning when not in need<br>6.4.3. **Not** reducing air conditioning when not in need goes against my principles<br>6.4.4. Everybody should share the responsibility to reduce air conditioning when not in need<br><br>6.5.1. It would be wrong of me **not** to reduce driving, and instead walk, bike or use public transportation<br>6.5.2. I would feel guilty if I did **not** reduce driving, and instead walk, bike or use public transportation<br>6.5.3. **Not** reducing driving, and instead walk, bike or use public transportation goes against my principles<br>6.5.4. Everybody should share the responsibility to reduce driving, and instead walk, bike or use public transportation<br><br>6.6.1. It would be wrong of me **not** to eat less meat and more vegetables<br>6.6.2. I would feel guilty if I did **not** eat less meat and more vegetables<br>6.6.3. **Not** eating less meat and more vegetables goes against my principles<br>6.6.4. Everybody should share the responsibility to eat less meat and more vegetables<br><br>6.7.1. It would be wrong of me **not** to buy local products or locally produced foods<br>6.7.2. I would feel guilty if I did **not** buy local products or locally produced foods<br>6.7.3. **Not** buying local products or locally produced foods goes against my principles<br>6.7.4. Everybody should share the responsibility to buy local products or locally produced foods<br><br>6.8.1. It would be wrong of me **not** to buy energy efficient appliances<br>6.8.2. I would feel guilty if I did **not** buy energy efficient appliances<br>6.8.3. **Not** buying energy efficient appliances goes against my principles<br>6.8.4. Everybody should share the responsibility to buy energy efficient appliances<br><br>6.9.1 It would be wrong of me **not** to reduce using plastic bags, or **not** to use own bag when shopping<br>6.9.2. I would feel guilty if I did **not** reduce using plastic bags, or use own bag when shopping

	6.9.3. **Not** reducing the use of plastic bags, or **not** using own bag when shopping goes against my principles<br>6.9.4. Everybody should share the responsibility to reduce using plastic bags, or use own bag when shopping<br><br>6.10.1 It would be wrong of me **not** to bring own utensils when eating out<br>6.10.2. I would feel guilty if I did **not** bring own utensils when eating out<br>6.10.3. **Not** bringing own utensils when eating out goes against my principles<br>6.10.4. Everybody should share the responsibility to bring own utensils when eating out
Wan, C., Cheung, R., & Shen, G. Q. (2012). Recycling attitude and behaviour in university campus: A case study in Hong Kong. Facilities.	
**7.1. Scale: EPQ - Relativism**	**Item**
Likert: 1 (SD) – 7 (SA)	Please indicate the extent to which you disagree or agree with the following statements:<br><br>7.1.1.There are no ethical principles that are so important that they should be a part of any code of ethics<br>7.1.2.What is ethical varies from one situation and society to another<br>7.1.3.Moral standards should be seen as being individualistic; what one person considers to be moral may be judged to be immoral by another person<br>7.1.4.Different types of moralities cannot be compared as to "rightness"<br>7.1.5.Questions of what is ethical for everyone can never be resolved since what is moral or immoral is up to the individual<br>7.1.6.Moral standards are simply personal rules that indicate how a person should behave, which should not be applied in making judgments of others<br>7.1.7.Ethical considerations in interpersonal relations are so complex that individuals should be allowed to formulate their own individual codes<br>7.1.8.Rigidly codifying an ethical position that prevents certain types of actions could stand in the way of better human relations and adjustment<br>7.1.9.No rule concerning lying can be formulated; whether a lie is permissible or not permissible totally depends upon the situation<br>7.1.10.Whether a lie is judged to be moral or immoral depends upon the circumstances surrounding the action
Forsyth, D. R. (1980). A taxonomy of ethical ideologies. Journal of Personality and Social psychology, 39(1), 175.	
**7.2. Scale: Scale: EPQ - Idealism**	**Item**
Likert: 1 (SD) – 7 (SA)	Please indicate the extent to which you disagree or agree with the following statements:<br><br>7.2.1. A person should make certain that their actions never intentionally harm another even to a small degree<br>7.2.2. Risks to another should never be tolerated, irrespective of how small the risks might be<br>7.2.3. The existence of potential harm to others is always wrong, irrespective of the benefits to be gained<br>7.2.4. One should never psychologically or physically harm another person<br>7.2.5. One should not perform an action which might in any way threaten the dignity and welfare of another individual

	7.2.6. If an action could harm an innocent other, then it should not be done<br>7.2.7. Deciding whether or not to perform an act by balancing the positive consequences of the act against the negative consequences of the act is immoral<br>7.2.8. The dignity and welfare of the people should be the most important concern in any society<br>7.2.9. It is never necessary to sacrifice the welfare of others<br>7.2.10 Moral actions are those which closely match ideals of the most "perfect" action
Forsyth, D. R. (1980). A taxonomy of ethical ideologies. Journal of Personality and Social psychology, 39(1), 175.	
**e. Attention check 5**	**Item**
[Open answer]	Please read the following scenario:<br>It's lunchtime and Cindy leaves the office and heads to the café with her colleagues. The café provides throwaway plastic cutlery (forks, knives, etc.), but Cindy normally brings her own metal, washable cutlery instead. Once she places her order, Cindy realized that she has left her cutlery at an office. The café is only a few minutes walk from the office, but it has started raining and she hasn't brought an umbrella.<br><br>[Next page]<br>Please write 2-3 sentences summarising the key details of the scenario that has been presented on the previous page
**8. Demographics**	**Item**
1. [Open answer]	Please type your current country of residence
2. [Open answer]	Please type your country of origin
3. Multiple choice	Please indicate your gender assigned at birth:<br>1. Male<br>2. Female<br>3. Prefer not to say
4. Multiple choice	Please indicate your age range:<br>1. 18-24<br>2. 25-34<br>3. 35-49<br>4. 50-64<br>5. 65 and over
5. Multiple choice	Please indicate your marital status:<br>1. Single (never married)<br>2. Married (no children)<br>3. Married (with children)<br>4. Domestic partnership<br>5. Divorced<br>6. Widowed<br>7. Separated
6. Multiple choice	Please indicate your level of education:

	1. High school or less<br>2. Some college<br>3. Undergraduate<br>4. College graduate<br>5. Post collegiate<br>6. None of the above
7. Multiple choice	Please indicate your net monthly household income per annum:<br><br>1. Less than $9,999<br>2. $10,000 - $19,999<br>3. $20,000 - $29,999<br>4. $30,000 – 39,999<br>5. $40,000 - $49,999<br>6. $50,000 – 74,999<br>7. $75,000 or more
8. Multiple choice	Please indicate your employment status:<br><br>1. Full time<br>2. Part time<br>3. Self employed<br>4. Unemployed<br>5. Retired<br>6. Student<br>7. Other
