# Supplementary material for: The Theory of Planned Behaviour doesn’t reveal ’attitude-behaviour’ gap? Contrasting the effects of moral norms vs. idealism and relativism in predicting pro-environmental behaviours
Source: PLoS One. 2023 Nov 27;18(11):e0290818. doi: 10.1371/journal.pone.0290818 (PMC10681191; doi:10.1371/journal.pone.0290818)
Supplement: Attachment — Submitted filename: Response to Reviewers.docx [file pone.0290818.s018.docx]

PONE-D-22-33487

The Theory of Planned Behaviour Doesn't Reveal 'Attitude-Behaviour' Gap? Contrasting the Effects of Moral Norms vs. Idealism and Relativism in Predicting Pro-Environmental Behaviours

**Response to reviewers**

| **Reviewer #1:** | |
| --- | --- |
| **Reviewer’s comment** | **Author’s response** |
| No comments | No comments |
|  | |
| **Reviewer #2:** | |
| **Reviewer’s comment** | **Author’s response** |
| (1) Some updated and classic/related literature about TPB & Pro-environmental behavior were not mentioned/cited yet. Having symbolic and impactful literature review support will much enhance your arguments in no matter hypotheses development or theoretical/practical contribution. I suggest the author(s) to consider reading and citing the following updated literature; sure the authors can cite other similar papers not limit to listed ones.  -Mo. Z., Liu M., Liu, Y. (2018). Effects of functional green advertising on self and others, Psychology & Marketing. 35(5), 368-382.  -Wang, S., Liu, M.T. and Pérez, A. (2022), "A bibliometric analysis of green marketing in marketing and related fields: From 1991 to 2021", Asia Pacific Journal of Marketing and Logistics, Vol. ahead-of-print No. ahead-of- print. <https://doi.org/10.1108/APJML-07-2022-0651>   -Liu, M., Liu, Y., Mo, Z. (2020). Moral norm Is the key: An extension of the theory of planned behaviour (TPB) on Chinese consumers’ green purchase intentions, Asia Pacific Journal of Marketing and Logistics, 32(8), 1823-1841.   - Liu, Y., Liu, M., Pérez, A., Chan, W., Collado, J., Mo, Z. (2021). The importance of knowledge and trust for ethical fashion consumption, Asia Pacific Journal of Marketing and Logistics,33(5), 1175-1194. | The focus of this article includes the review of TPB when (i) both intention and behaviour were measured using the TPB model, and (ii) either plain TPB, moral norms or EPQ were included. Hence, the suggested literature falls out of scope of this article:  Does not include TPB  Does not include TPB  Does not include both intention and behaviour (model explores intention only)  Does not include TPB |
| (2) About Format, Tables & Figures demonstration: Please revise the format based on publication standard/requirements set by Plos One before resubmission so it can save publisher's time to proceed your publicaiton. | Thank you. We have edited the in-text statistical reporting, tables, and figures in the main body to adhere to Vancouver referencing. |
|  | |
